# Bayesian inference of multi-point macromolecular architecture mixtures at nanometre resolution

**DOI:** 10.1371/journal.pcbi.1010765

**Published:** 2022-12-27

**Authors:** Peter A. Embacher, Tsvetelina E. Germanova, Emanuele Roscioli, Andrew D. McAinsh, Nigel J. Burroughs

**Affiliations:** 1 Department of Medical Physics & Biomedical Engineering, University College London, London, United Kingdom; 2 Centre for Mechanochemical Cell Biology and Division of Biomedical Sciences, Warwick Medical School, University of Warwick, Coventry, United Kingdom; 3 Mathematics Institute and Zeeman Institute, University of Warwick, Coventry, United Kingdom; Georgia Institute of Technology and Emory University, UNITED STATES

## Abstract

Gaussian spot fitting methods have significantly extended the spatial range where fluorescent microscopy can be used, with recent techniques approaching nanometre (nm) resolutions. However, small inter-fluorophore distances are systematically over-estimated for typical molecular scales. This bias can be corrected computationally, but current algorithms are limited to correcting distances between pairs of fluorophores. Here we present a flexible Bayesian computational approach that infers the distances and angles between multiple fluorophores and has several advantages over these previous methods. Specifically it improves confidence intervals for small lengths, estimates measurement errors of each fluorophore individually and infers the correlations between polygon lengths. The latter is essential for determining the full multi-fluorophore 3D architecture. We further developed the algorithm to infer the mixture composition of a heterogeneous population of multiple polygon states. We use our algorithm to analyse the 3D architecture of the human kinetochore, a macro-molecular complex that is essential for high fidelity chromosome segregation during cell division. Using triple fluorophore image data we unravel the mixture of kinetochore states during human mitosis, inferring the conformation of microtubule attached and unattached kinetochores and their proportions across mitosis. We demonstrate that the attachment conformation correlates with intersister tension and sister alignment to the metaphase plate.

## Author summary

Light microscopy is an exceptionally powerful tool for observing and measuring biological structures and dynamics. However, spatial resolution is limited by the wavelength of the utilised light, 400–700 nanometres in the visible spectrum, traditional imaging being limited to essentially the wavelength itself. This is because point source objects appear under the microscope as a spot with dimensions the order of the wavelength. For sufficiently well spaced point source like objects spot fitting can be used. The spot centres can be localised with nanometre accuracy, and thus the distance between centres of spots in different colours can in turn be determined with nanometre spatial resolution. By labelling different parts of a molecular structure in different colours, the structure can be mapped in 3D with high spatial resolution. However, these distances are in fact systematically over estimated because of spot centre measurement error. We present an algorithm that corrects this overestimation for multi-fluorophore images, inferring the full geometry of the labelled structure, giving greater accuracy over previous methods, particularly for small distances. We also present an algorithm that can unravel mixtures of different conformational (geometric) states. We demonstrate our algorithm on the kinetochore macro-molecular complex and its change in structure during cell division.

## Introduction

The classical Rayleigh criterion limits the resolution of light microscopy to about 200nm for typical wavelengths and numerical apertures. However, this limit can in principle be pushed arbitrarily close to zero by fitting the point spread function (PSF) to diffraction limited objects [1–3]. In this case the localisation accuracy is primarily limited by the finite signal to noise (S/N) ratio, a consequence of the finite photon count [4, 5]. By using multiple fluorophores to label diffraction limited objects, inter-object distances can thus be measured, achieving a localisation accuracy of typically tens of nm with standard fluorescence microscopes and fluorophores.

**Fig 1:**
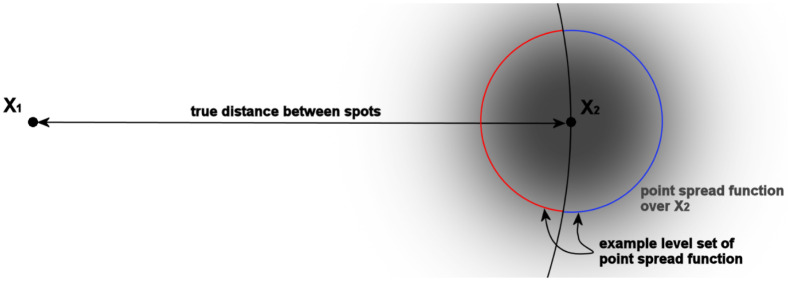
Schematic of the inflation of distances with diffraction limited spot measurements. The blue and red arcs together form a level set of the point spread function of spot 2 at true position *X*_2_ (grey). Thus the measured position of spot 2 is equally likely on any position on these arcs. The blue arc being longer than the red arc implies that spot 2 is more likely to be measured further away from spot 1 than their true distance. On average the measured distance is thus larger than the true distance.

Pooling multiple measurements can address the limitations of low signal to noise ratio, but a more fundamental problem remains for small inter-fluorophore distances: if the distance between two fluorescent spots is of the order of spot centre accuracy, the measured (Euclidean) distance systematically over-estimates the true distance, [6]. This over-estimation, or inflation, is a consequence of distances being convex functions (see Jensen’s inequality, [7, Thm 3.1.3]) and can be understood as a consequence of Euclidean distances having spherical level sets, Fig 1. The Euclidean distance is thus an inconsistent biased estimator. This bias also impacts polygon shape, for instance measured triangles become more equilateral (internal angles are biased towards 60°).

**Fig 2:**
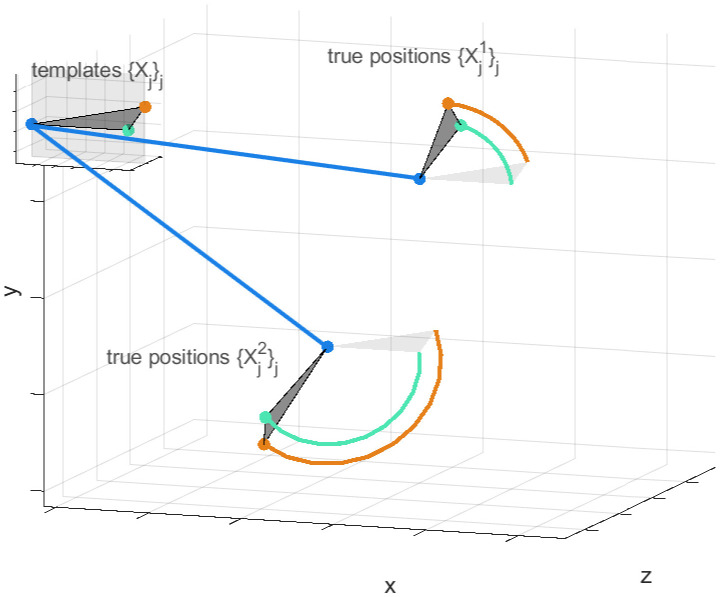
Template and perspectives. Inferred true polygon positions ${\left\{X_{j}^{n}\right\}}_{j=1..J,n=1...N}$ and template positions {*X*_*j*_}_*j* = 1,2, ..*J*_ for *J* = 3 fluorophores and *N* = 2 measurements are shown. Each true polygon is generated from the template by a translation (straight blue lines, positioning the first nodes ${\left\{X_{1}^{n}\right\}}_{n}$) and rotation (circular lines). The measured positions ${\left\{{\tilde{X}}_{j}^{n}\right\}}_{j,n}$ deviate from the true positions due to measurement error, but are not shown for simplicity.

The over-estimation of pair-wise distances is well understood, with computational correction methods being available, [6, 8, 9]. In particular, in the case of isotropic Gaussian measurement errors the likelihood is analytically tractable in 1–3 dimensions, [6, 8], and has been used across a variety of applications, [10, 11]. In 3D, however, typically PSFs are anisotropic, with the resolution along the optical axis usually reduced relative to that in the focal plane. For anisotropic measurement errors no analytical solution is known; however a Bayesian approach was developed for pair-wise distance correction [12]. Here we extend this methodology to multiple fluorophores/arbitrary polygons. Specifically we develop a Bayesian sampling algorithm (using a Markov Chain Monte Carlo (MCMC) framework) to infer a fixed polygon (referred to as the template) from measured polygons, the polygon nodes being marked with distinct fluorophores. Each measured polygon is rotated and translated relative to the template, Fig 2. This polygon based method has several benefits over pair-wise methods. Firstly, the geometric constraints are automatically satisfied in our model, e.g. for three fluorophores the triangle inequality holds. This contrasts to inferring the three lengths independently in a pair-wise fashion, where the inferred lengths do not necessarily make a triangle. Secondly, geometric correlations between the lengths can improve individual length confidence (reducing posterior variances). Thirdly, individual localisation errors of each fluorophore are inferred; this contrasts to pair-wise analyses where only the error of the displacement can be inferred.

Our method can achieve localisation accuracies down to the single nanometre range on spinning disk confocal microscope images. It is an image post-processing methodology and is thus distinct from super-resolution techniques like Photo-Activated Localization Microscopy (PALM, [13]), Super-Resolution Structured Illumination Microscopy (SR-SIM, [14]) or Super-Resolution Radial Fluctuations (SRRF, [15]) with resolutions below the diffraction limit. Our method could in fact be used to post-process images from such microscopes, to infer protein architectures with higher resolution. Our statistical method requires a number of assumptions: we assume spot-like localisation of fluorophores and that there is a common polygonal architecture between the fluorophores shared among all measurements (or multiple architectures in heterogeneous mixture analysis, see below). In this regard our method is related to averaging techniques ([16, 17]), that overlay multiple measurements of the same object to improve resolution. However, instead of minimising a cost-function to align multiple measurements we use a Bayesian inference approach. This difference is vital, because within the Bayesian framework we can correct the systematic distortions described above for lengths and polygonal shape. There is a variety of co-localisation methods, based on Spearman or Manders coefficients (e.g. [18, 19]), however these do not directly estimate geometric parameters such as lengths or angles. Auto- and cross-correlation based methods do get length scales ([20]), but require assumptions on the larger-scale distribution of the measured objects and are limited to pair-wise measurements.

To address data heterogeneity, we extended our algorithm to infer multiple polygons across a group of dataset(s). This allows us to study conformation changes of macro-molecular complexes *in situ* and correlate conformation with other (macroscopic) measurements. Our method is distinct from, but complements methods based on proximity sensors such as Förster Resonance Energy Transfer (FRET, [21]). We apply a two-state version of our algorithm to human kinetochores, macro-molecular complexes that play a vital role during cell division. Kinetochores control attachments to microtubules that are critical for the correct segregation of chromosomes to each daughter cell. Microtubule attachment in fact induces a conformational change within the kinetochore macro-molecular complex, [12]. By using our two-state model on triple-labelled kinetochore data from metaphase and prometaphase cells, we demonstrate that in early prometaphase the majority of kinetochores is similar to, but distinct from unattached kinetochores observed in depolymerised microtubule conditions (nocodazole) [12]. In late prometaphase and metaphase this state becomes increasingly infrequent. We demonstrate that these unattached early prometaphase kinetochores are under lower mechanical force than attached kinetochores.

This paper is organised as follows. Section Materials and methods presents the model and Bayesian inference methodology. Section Sampling based inference of model parameters: A Markov Chain Monte Carlo algorithm gives an outline of the inference algorithm. Full details of the algorithms can be found in the Supplementary Data Markov Chain Monte Carlo samplers for parameter inference in S1 Text. We demonstrate the accuracy of our algorithm on simulated/synthetic data in section Algorithm performance on simulated data, accurately inferring polygon side lengths, internal angles and the fluorophore measurement errors, thereby demonstrating the algorithm’s advantages over existing pair-wise analyses. For heterogeneous mixture analysis, we demonstrate accurate inference of the states and the state proportions in the mixture. In section Analysis of the human kinetochore: A structured macro-molecular complex we use our algorithm to analyse the architecture of the human kinetochore from experimental 3D fluorescence imaging data, [12]. In subsection Experimental dataset analysis with multi-state model algorithm we analyse heterogeneity in the kinetochore conformation in prometaphase and metaphase cells, demonstrating that a jack-knife like Ndc80 conformation [12] dominates in early prometaphase, whilst a straightened Ndc80 conformation dominates in metaphase. In Discussion we outline improvements and the limitations of our current algorithm.

## Materials and methods

To highlight the model’s generality, the model is presented for an arbitrary number *J* of distinct fluorophores. We later restrict our analysis to examples with three fluorophores (*J* = 3, referred to as *triangle correction*). We initially assume that there is a single polygonal state, and then extend this model to mixtures of states in subsection Mixture of multiple states.

We consider fixed three-dimensional fluorescent images labelled with a number *J* of distinct fluorophores, located at different sites within a macro-molecular structure. Here a site refers to part of the structure where the respective fluorescent labels cluster, *i.e*. each fluorophore occurs in multiple copies within the complex. In practice, each fluorescent spot should be sufficiently tightly localised for the Gaussian spot approximation to be valid, with a width similar to a diffraction limited object. Fluorescent labels can be fluorescently labelled antibodies, labelled DNA (Fluorescence in situ hybridisation, FISH), or a genetically encoded fluorophore such as green fluorescent protein (GFP). The objective is to infer the underlying polygon (size, edge lengths, angles between edges) from a set of measured fluorophore positions, where for each measurement the *J* sites are located at the nodes of the polygon. The true polygon positions are unknown because of measurement noise.

### Model assumptions

We assume there are *N* measurements of the locations of the *J* nodes of *J*-sided polygons in 3D, where the nodes are distinguished by specific fluorophore colours. These measurements are assumed to arise from the same underlying polygon template by a rotation and translation in space (specific to each measurement; see Fig 2) but subject to measurement error; the measured polygons thus deviate in size and shape from the template polygon. For each measurement, the combination of the 3D rotation and translation are referred to as its *perspective*. We assume space is uniform, so the (true) polygons are uniformly distributed throughout space and undergo an isotropic rotation.

We assume that the measurement errors are anisotropic Gaussians with covariance matrix $\left(\begin{array}{ccc} \sigma_{j;xy}^{2} & 0 & 0\\ 0 & \sigma_{j;xy}^{2} & 0\\ 0 & 0 & \sigma_{j;z}^{2} \end{array}\right)$ in the given coordinate system (*z* along the optical axis) for fluorophore type *j* ∈ {1, …, *J*}; previous pair-wise methods also assume Gaussian errors, [6] (isotropic) and [9] (anisotropic). Note, that only the shape of the measurement error is assumed; *σ*_*j*;*xy*_, *σ*_*j*;*z*_ are to be inferred. Errors are fluorophore specific because quantum efficiency differs between fluorophores (photon count emission), and may also depend on the imaging conditions and the number of labelled fluorophores at a site. Data typically consists of spot centre locations, as for instance determined by fitting 3D Gaussian profiles to spots in the image (either individually or using a mixture of Gaussians model).

### Polygon parametrisation and inference

The underlying polygon is defined by a standardised template, with node *j* ∈ {1, …, *J*} at (column) vector position $X_{j}\in {\mathbb{R}}^{3}$. In the following we refer to this as the template, and the nodes as the template positions. We write {*X*_*j*_}_*j*_ to denote the set of all template positions ({*X*_*j*_}_*j*∈{1,…,*J*}_). We use a similar notation for other parameters. We choose the template such that *X*_1_ is at the origin, *X*_2_ is on the non-negative *x*-axis and *X*_3_ is in the *x*-*y*-plane with non-negative *y*. This standardisation of the template is without loss of generality, as the template is otherwise only defined up to the perspectives. A perspective is associated with each measurement *n* ∈ {1, …, *N*}, defining the true polygon positions ${\left\{X_{j}^{n}\right\}}_{j}$ of that measurement relative to the template positions. The perspective for a particular measurement *n* has six parameters in total: the first three define a translation (column) vector $T^{n}\in {\mathbb{R}}^{3}$ of the true position $X_{1}^{n}$ of polygon node *j* = 1 relative to *X*_1_; the next three are the Euler angles of the three-dimensional rotation matrix $R^{n}\in {\mathbb{R}}^{3\times 3}$ (the matrix representation of *SO* (3)) of the true polygon positions relative to the template, with centre of rotation at polygon node *j* = 1, Fig 2. Thus, the true position of each polygon node *j*, measurement *n* is given by:

$$\begin{array}{c} X_{j}^{n}=R^{n}\cdot (X_{j}-X_{1})+X_{1}+T^{n}. \end{array}$$ (1)

The measured fluorophore position (column) vector ${\tilde{X}}_{j}^{n}\in {\mathbb{R}}^{3}$ incorporates (Gaussian) measurement error ${\tilde{X}}_{j}^{n}=X_{j}^{n}+\gamma_{j}^{n}$, $\gamma_{j}^{n}∼N\left(0;\tau_{j}^{-1}\right)$ independently for all *j* and *n*, with (3 × 3) precision matrix $\tau_{j}=\left(\begin{array}{ccc} \sigma_{j;xy}^{-2} & 0 & 0\\ 0 & \sigma_{j;xy}^{-2} & 0\\ 0 & 0 & \sigma_{j;z}^{-2} \end{array}\right)$. The likelihood for the model parameters {*θ*_*j*_}_*j*_ ≔ {{*X*_*j*_}_*j*_, {*τ*_*j*_}_*j*_}, *j* ∈ {1, …, *J*}, and perspectives {*ϑ*^*n*^}_*n*_ ≔ {{*T*^*n*^}_*n*_, {*R*^*n*^}_*n*_}, *n* over the set of measurements {1, …, *N*}, reads:

$$\begin{array}{c} L[{\left\{\theta_{j}\right\}}_{j},{\left\{\vartheta^{n}\right\}}_{n}|{\left\{{\tilde{X}}_{j}^{n}\right\}}_{j,n}]=(\prod_{j,n}\sqrt{{\left(2\pi \right)}^{-3}\text{det}(\tau_{j})}\;e^{-\frac{1}{2}{∥X_{j}^{n}-{\tilde{X}}_{j}^{n}∥}_{\tau_{j}}^{2}}), \end{array}$$ (2)

where index ranges are suppressed for simplicity, and ${\left\|u\right\|}_{\tau_{j}}^{2}≔u^{T}\cdot \tau_{j}\cdot u$ denotes the squared Euclidean norm of (column) vector $u\in {\mathbb{R}}^{3}$, weighted with *τ*_*j*_ (here *T* denotes the transpose). The predicted true positions $X_{j}^{n}$ for node *j*, measurement *n*, is given in Eq (1). The likelihood thus has a dependence on the hidden perspective variables *T*^*n*^, *R*^*n*^.

The posterior *dπ* is given, up to proportionality, by multiplying the likelihood from Eq (2) with the prior, denoted *dπ*_0_[{*θ*_*j*_}_*j*_, {*ϑ*^*n*^}_*n*_]. We use uninformative priors, namely translations are homogeneously distributed in space, rotations are isotropic, measurement errors are flat on the positive half-space and the prior on the template positions is (approximately) flat on the marginal of each polygon length |*X*_*j*_ − *X*_*i*_|. For details, see section Uninformative priors in S1 Text.

### Mixture of multiple states

The above model assumes population homogeneity, *i.e*. there is only a single underlying polygon state, positions {*X*_*j*_}_*j*_, from which all measured polygons, ${\left\{{\tilde{X}}_{j}^{n}\right\}}_{j,n}$, arise (up to measurement noise and perspective). This is a strong assumption, and may be violated in applications. Specifically there may be a mixture of underlying polygon states with distinct polygon templates and measurement errors for each state. Here we extend the model to allow for heterogeneity of the (polygon) state; i.e. measurements originate from one of *Z* polygon templates ${\left\{X_{j}^{\left(\zeta \right)}\right\}}_{j,\zeta }$ for *ζ* ∈ {1, …, *Z*}. In addition, the corresponding measurement errors may vary between the states, *i.e*. we have

$$\begin{array}{c} \tau_{j}^{\left(\zeta \right)}=\left(\begin{array}{ccc} {\left(\sigma_{j;xy}^{\left(\zeta \right)}\right)}^{-2} & 0 & 0\\ 0 & {\left(\sigma_{j;xy}^{\left(\zeta \right)}\right)}^{-2} & 0\\ 0 & 0 & {\left(\sigma_{j;z}^{\left(\zeta \right)}\right)}^{-2} \end{array}\right) \end{array}$$ (3)

extending the notation above. In this case we not only want to infer for each state *ζ* the template positions ${\left\{X_{j}^{\left(\zeta \right)}\right\}}_{j}$ and measurement errors ${\left\{\sigma_{j;d}^{\left(\zeta \right)}\right\}}_{j,d}$, and for each measurement *n* the perspectives, but also the proportion *p*^(*ζ*)^ ∈ [0, 1] of each state in the mixture.

Analogous to Eq (1) the predicted true positions are in this case:

$$\begin{array}{c} X_{j}^{n}=R^{n}\cdot \left(X_{j}^{\left(\zeta^{n}\right)}-X_{1}^{\left(\zeta^{n}\right)}\right)+X_{1}^{\left(\zeta^{n}\right)}+T^{n}. \end{array}$$ (4)

Note that all the *J* fluorophore positions of the *n*^*th*^ measurement derive from state *ζ*^*n*^. These state-affiliations {*ζ*^*n*^}_*n*_, as well as the proportions $P≔{\left\{p^{\left(\zeta \right)}\right\}}_{\zeta }$ with which each state enters into the mixture are hidden variables, which need to be inferred. For the state proportions, {*p*^(*ζ*)^}_*ζ*_, there is no prior preference for any state, i.e. we use a flat Dirichlet distributed (hyper-) prior (see Eq (W) in S1 Text for the distribution):

$$\begin{array}{c} P∼\text{Dir}\left(1,\ldots ,1\right). \end{array}$$ (5)

For the state affiliations, {*ζ*^*n*^}_*n*_, we use a generalised Bernoulli-distributed prior (also known as the multinoulli or categorical distribution) on *Z* categories for the states, *i.e*. $\zeta^{n}∼Cat(P)$ independently for each *n*, where Cat(P) denotes the categorical distribution with parameters P, Eq (5).

Thus, the likelihood function of the extended model reads:

$$\begin{array}{c} \left(\prod_{j,n}\text{det}{\left(\tau_{j}^{\left(\zeta^{n}\right)}\right)}^{\frac{1}{2}}\cdot e^{-\frac{1}{2}{∥X_{j}^{n}-{\tilde{X}}_{j}^{n}∥}_{\tau_{j}^{\left(\zeta^{n}\right)}}^{2}}\right), \end{array}$$ (6)

where $X_{j}^{n}$ is the state-dependent Eq (4), while the prior of the extended model is:

$$\begin{array}{c} \left(\prod_{\zeta \in \{1,\ldots ,Z\}}d\pi_{0}\left({\left\{\theta_{j}^{\left(\zeta \right)}\right\}}_{j},{\left\{\vartheta^{n}\right\}}_{n}\right)\right)\left(\prod_{n\in \{1,\ldots ,N\}}p^{\left(\zeta^{n}\right)}\right)\times \\ \delta \left(\left(\sum_{\zeta \in \{1,\ldots ,Z\}}p^{\left(\zeta \right)}\right)-1\right)\cdot \left(\prod_{\zeta \in \{1,\ldots ,Z\}}dp^{\left(\zeta \right)}\right), \end{array}$$ (7)

where *p*^(*ζ*)^ are valued in [0, 1], and ${\left\{\theta_{j}^{\left(\zeta \right)}\right\}}_{j}=\left\{{\left\{X_{j}^{\left(\zeta \right)}\right\}}_{j},{\left\{\tau_{j}^{\left(\zeta \right)}\right\}}_{j}\right\}$. Note, that this reduces to the basic single-state version described before when *Z* = 1.

In practice, we found for mixture models that uninformative priors, Eq (7), can result in poor convergence. There were two issues. Firstly, if a state is “lost” during a step in the Markov chain, (i.e. state *ζ*′ does not occur, ∀*n* : *ζ*^*n*^ ≠ *ζ*′), then its reappearance can require a substantial number of steps. For that reason, we impose that each state has to occur at least three times (i.e. |{*n* ∈ {1, …, *N*}|*ζ*^*n*^ = *ζ*′}| ≥ 3 for all *ζ*′ ∈ {1, …, *Z*}). In all examples considered in the main text, the posterior was far away from this boundary, so the constraint did not affect the posterior. The second issue is when a polygon state is rare, then both the posterior shape and size of the rare state polygon, and its proportion *p*^(2)^ have low confidence (the rare state is *ζ* = 2). To address this, we assume the data consists of several subsets that comprise the same states, *i.e*. the templates and measurement errors are common across the subsets, ${\left\{\theta_{j}^{\left(\zeta \right)}\right\}}_{j,\zeta }$, but the subsets have independent state proportions ${\left\{p_{\text{subset}\;s}^{\left(\zeta \right)}\right\}}_{\zeta ,s}$. This gives the flexibility to jointly analyse datasets from different biological conditions, thereby determining their similarities and differences. If each state is prevalent in one of these subsets, and these are sufficiently informative, the posterior will distinguish the states. A homogeneity constraint can be imposed on a dataset (i.e. only one of the states is present) if justified. In this case the dataset is called a *state-informing dataset*. The likelihood for joint inference is the product of the likelihoods for each dataset, either the multi-state likelihood for mixed datasets or the single-state likelihood for the pure datasets.

Since the perspectives are inferred per measurement, there are a large number of inferred parameters, see formulae in supplementary section Number of parameters in the model in S1 Text. For instance, in the single-state Example 1.1 there are 2002 and 2409 inferred parameters for the pair-wise (one single length) and triangle correction, respectively. For two-state Example 2.1 we have 7819 inferred parameters for the triangle correction. Throughout this text, examples are named by the table number/letter and a unique identifier within the table: (table number/letter).(example number within the table).

### Sampling based inference of model parameters: A Markov Chain Monte Carlo algorithm

To infer the model parameters—the template and measurement errors {*θ*_*j*_}_*j*_ and the measurement specific perspectives {*ϑ*^*n*^}_*n*_ (and for the multi-state model, the state affiliations {*ζ*^*n*^}_*n*_ and proportions {*p*^*ζ*^}_*ζ*_ as well)—we use a Bayesian computational method (see e.g. [22, Part 1]). Specifically, for the single-state model the (so-called posterior) probability of the parameters given the data is, up to proportionality,

$$\begin{array}{c} d\pi [{\left\{\theta_{j}\right\}}_{j},{\left\{\vartheta^{n}\right\}}_{n}|{\left\{{\tilde{X}}_{j}^{n}\right\}}_{j,n}]\propto L[{\left\{\theta_{j}\right\}}_{j},{\left\{\vartheta^{n}\right\}}_{n}|{\left\{{\tilde{X}}_{j}^{n}\right\}}_{j,n}]\cdot d\pi_{0}[{\left\{\theta_{j}\right\}}_{j},{\left\{\vartheta^{n}\right\}}_{n}], \end{array}$$ (8)

with the likelihood from Eq (2) and prior *π*_0_ from Eq (A) in S1 Text. There are a range of algorithms that can be used to sample from this posterior. We use a Markov Chain Monte Carlo (MCMC) methodology, [23, Ch 1], [24, Ch 1], whereby a Markov chain is constructed that has a stationary distribution equal to the posterior distribution. Once converged, the chain can be used to sample from the posterior. MCMC is a powerful and flexible method for simulating from the posterior Eq (8), propagating measurement errors through to inferred parameters. It is thus ideal for this problem.

In the single-state model we sequentially update each of the parameters separately: the perspectives, {*R*^*n*^}_*n*_, {*T*^*n*^}_*n*_, for each polygon measurement *n*, the template positions, {*X*_*j*_}_*j*_, and the precisions, {*τ*_*j*_}_*j*_ for each node *j*. For {*X*_*j*_}_*j*_, {*R*^*n*^}_*n*_ and {*T*^*n*^}_*n*_, random walk samplers are used, while for {*T*^*n*^}_*n*_ and {*τ*_*j*_}_*j*_ we use Gibbs samplers.

In the multi-state model we sequentially update the perspectives just as in the single-state model, while each of the template positions ${\left\{X_{j}^{\left(\zeta \right)}\right\}}_{j,\zeta }$ and precisions ${\left\{\tau_{j}^{\left(\zeta \right)}\right\}}_{j,\zeta }$ is updated sequentially. The state proportions {*p*^(*ζ*)^}_*ζ*_ are updated jointly to always satisfy their normalisation condition. For each of the ${\left\{X_{j}^{\left(\zeta \right)}\right\}}_{j,\zeta }$, the {*R*^*n*^}_*n*_, the {*T*^*n*^}_*n*_ and the {*ζ*^*n*^}_*n*_ random walk samplers are used, while for {*T*^*n*^}_*n*_, ${\left\{\tau_{j}^{\left(\zeta \right)}\right\}}_{j,\zeta }$ and {*p*^(*ζ*)^}_*ζ*_ we use Gibbs samplers. See subsection Markov Chain Monte Carlo samplers for parameter inference in S1 Text for algorithm details.

## Results

### Algorithm performance on simulated data

We test our polygon inference algorithm on simulated data for both the single-state and the multi-state models, confirming that our algorithms reproduce the true original parameters of the simulations. For the single-state model there are existing methods for length correction: the pair-wise Bayesian Euclidean Distance Correction Algorithm (BEDCA, [9]), and the analytic length correction based on [6]. The latter is only applicable to isotropic measurement errors and comes in two variations, a maximum likelihood estimate developed in [6] (“MLE” below) and a full posterior probability version (“means” below; see Implementation of pair-wise correction methods in S1 Text). We compare our triangle algorithm to these methods (where applicable). Note we use a flat prior on the measurement errors in all methods for fair comparison. We confine ourselves to *J* = 3 fluorophores, referring to our method as the triangle correction (method); a four-fluorophore example is presented in supplementary section Four-fluorophore simulated example in S1 Text. For the multi-state model we use *Z* = 2 states.

#### Testing the single-state model on simulated data

**Fig 3:**
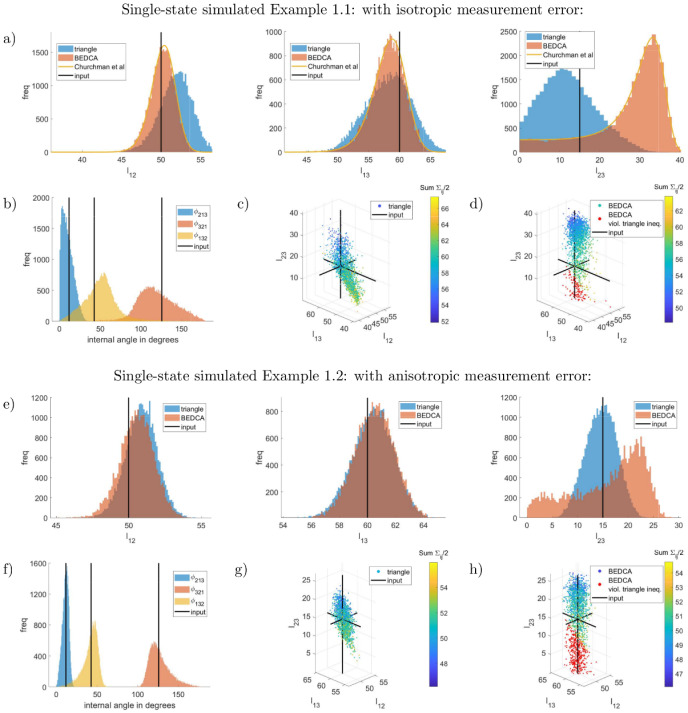
Posterior distributions for single-state simulated Examples 1.1, 1.2. Marginal inferred posteriors for lengths (in nm) and internal angles. For each example the top row (a,e) shows the marginal posteriors of the three triangle lengths as inferred by each of the correction methods. The bottom left images (b,f) show the inferred internal angles using the triangle correction presented here. The bottom centre images (c,g) show a scatter plot of the joint distribution as obtained with the triangle correction. The bottom right images (d,h) show the naive attempt to achieve a joint distribution of the three triangle lengths from the BEDCA method [9] by assuming independence. Samples that violate the triangle inequality are shown in red (about 7% of the samples in Example 1.1, 22% in Example 1.2), otherwise the colour represents the sum of all measurement errors, specifically $\sqrt{\frac{1}{2}\left(\sum_{i\neq j;d\in \left\{x,y,z\right\}}\sigma_{ij;d}^{2}\right)}$, to indicate the correlation between inferred lengths and errors. The plots show random sub-samples, to improve visibility. See Table 1 for the posterior means and standard deviations. See section Additional supplementary images in S1 Text for the corresponding Markov chain evolution of the parameters of the triangle inference.

We illustrate our algorithm on simulated data with (true) triangle lengths and measurement errors similar to those measured in biological complexes, [12]. We simulate data with *J* = 3 fluorophores and *N* = 400 independent measurements, using the core model described in subsection Polygon parametrisation and inference, see supplementary section Simulated data in S1 Text for details. True parameters and the corresponding inferred values are shown in Table 1 for six examples; see Fig 3 for the posterior distributions of the inferred lengths and internal angles. Our correction algorithm typically gives inferred values that are in good agreement with the true values. These examples clearly confirm that the Euclidean distance estimator $\frac{1}{N}\left(\sum_{n=1}^{N}\left|{\tilde{X}}_{j}^{n}-{\tilde{X}}_{i}^{n}\right|\right)$ is subject to significant length inflation.

**Table 1:** Single-state simulated examples (*N* = 400).

(lengths and errors in nm)	|*X*_2_ − *X*_1_|	|*X*_3_ − *X*_1_|	|*X*_3_ − *X*_2_|	*σ*_12;*xy*_	*σ*_12;*z*_	*σ*_13;*xy*_	*σ*_13;*z*_	*σ*_23;*xy*_	*σ*_23;*z*_
Example 1.1 with isotropic measurement error:									
true value	50	60	15	21.2	21.2	29.2	29.2	29.2	29.2
direct measurement	59.1±1.0	73.2±1.3	49.2±1.0	/	/	/	/	/	/
Churchman MLE, [6]	50.5±1.5	58.9±2.5	33.2±3.5	20.9±0.9		29.3±1.5		23.7±1.6	
Churchman means, [6]	50.2±1.5	58.2±2.7	27.0±9.3	21.1±1.0		29.7±1.6		25.6±2.6	
BEDCA, [9]	50.2±1.6	58.0±2.8	27.1±9.2	21.2±1.2	21.0±1.5	30.2±1.9	29.2±2.1	25.4±2.6	26.0±2.9
triangle, uninformative prior	51.8±2.1	58.2±3.6	12.3±6.7	20.3±1.5	23.0±2.2	31.3±2.6	29.2±2.7	30.5±1.6	31.9±1.9
Example 1.2 with anisotropic measurement error:									
true value	50	60	15	14.1	28.3	18.0	36.1	18.0	36.1
direct measurement	58.2±0.9	70.0±1.2	43.3±1.0	/	/	/	/	/	/
BEDCA, [9]	50.6±1.2	60.5±1.4	16.1±7.0	14.0±0.9	28.7±1.6	17.7±1.2	36.1±2.0	17.2±1.9	36.7±1.7
triangle, uninformative prior	50.9±1.1	60.4±1.5	14.7±3.0	13.6±0.8	29.3±1.6	18.3±1.2	34.7±1.9	18.1±0.9	37.2±1.4
Example 1.3 with anisotropic measurement error:									
true value	45	60	15	14.1	28.3	18.0	36.1	18.0	36.1
BEDCA, [9]	45.9±1.2	59.9±1.4	12.2±6.4	14.4±0.9	27.8±1.6	17.5±1.0	34.3±2.0	17.3±1.5	35.5±1.5
triangle, uninformative prior	46.2±1.1	59.8±1.4	15.4±1.8	14.2±0.8	27.7±1.5	17.4±1.0	34.9±1.9	16.9±0.6	35.3±1.3
Example 1.4 with anisotropic measurement error:									
true value	60	60	15	14.1	28.3	18.0	36.1	18.0	36.1
BEDCA, [9]	58.2±1.0	57.9±1.4	13.4±6.7	13.0±0.8	27.2±1.6	16.5±1.1	38.6±2.1	17.1±1.7	38.6±1.6
triangle, uninformative prior	58.4±1.0	57.4±1.4	15.1±3.9	12.8±0.8	27.5±1.5	17.2±1.1	38.8±1.8	16.9±1.1	38.7±1.5
Example 1.5 with anisotropic measurement error:									
true value	15	15	15	14.1	28.3	18.0	36.1	18.0	36.1
BEDCA, [9]	16.4±5.3	18.8±6.8	9.08±5.42	13.6±1.7	29.0±1.4	16.5±2.1	35.7±1.7	19.4±1.1	37.3±1.4
triangle, uninformative prior	17.5±4.4	17.8±4.4	7.77±5.15	13.4±1.5	28.8±1.4	17.2±1.3	36.1±1.5	19.7±1.0	37.5±1.4
Example 1.6 with anisotropic measurement error:									
true value	60	60	0	14.1	28.3	18.0	36.1	18.0	36.1
BEDCA, [9]	60.5±1.1	60.9±1.3	10.1±5.8	14.0±0.9	28.1±1.7	17.0±1.0	34.8±1.9	16.9±1.3	35.8±1.4
triangle, uninformative prior	60.6±1.0	60.9±1.1	3.5±2.9	13.9±0.8	28.2±1.6	17.6±0.9	33.1±1.7	18.1±0.5	36.4±1.3

Rows are the original (true) value, and the posterior means (or MLE) ± standard deviations in subsequent rows for stated method. ‘Direct measurement’ refers to the (uncorrected) Euclidean distance $\frac{1}{N}\left(\sum_{n=1}^{N}\left|{\tilde{X}}_{j}^{n}-{\tilde{X}}_{i}^{n}\right|\right)$, which is known to overestimate distances [8]. “Churchman means” refers to the posterior mean using a flat prior on the length and measurement error, *dl*_*ij*_*dσ*_*ij*_. We quote the pair-wise variances to compare to the pair-wise algorithm (for triangle we have individual fluorophore measurement errors to give pair-wise variance using $\sigma_{ij;d}≔\sqrt{\sigma_{j;d}^{2}+\sigma_{i;d}^{2}}$).

For inferred posterior distributions of the lengths see Fig 3.

All correction methods have similar performance for sufficiently large lengths (relative to the measurement error) and isotropic (Gaussian) measurement errors, Example 1.1, sides |*X*_2_ − *X*_1_|, |*X*_3_ − *X*_1_|. For small lengths, less or similar to the measurement error, all correction methods suffer from relatively large inference errors, length |*X*_3_ − *X*_2_| in our example with true value 15nm, (see Small lengths are increasingly difficult to infer in S1 Text for an explanation). Problems with the maximum likelihood estimate from [6] (“MLE” in Table 1) were reported previously, [10], with results becoming error-prone for lengths similar to, or smaller than the measurement error. For these short lengths we find this method tends to report misleading results with overconfident error-estimates (Example 1.1). For pair-wise inferences the likelihood with isotropic measurement errors is analytically tractable (Eq (6) in [6]); we can thus determine the posterior (assuming the same flat priors we use for the pair-wise method) and therefore calculate the posterior mean (“means” in Table 1). This estimate, and the pair-wise based Bayesian inference method [9] give near identical results (as expected as they share the same assumptions apart from the extra degree of freedom of [9] for anisotropic measurement errors). For the small length |*X*_3_ − *X*_2_|, the pair-wise based posterior has a long thick tail towards zero, see Fig 3a and 3c, indicating large uncertainty. The triangle correction has a posterior mean much closer to the true value and has substantially smaller variance, Example 1.1. This is because information is essentially shared between the three lengths, allowing inference of the smaller length to be improved. There is an associated increase in the uncertainty of the other two lengths relative to the pair-wise correction, Example 1.1.

Examples 1.2–1.6 in Table 1 are for anisotropic measurement errors. There are only two methods available for anisotropic errors: our triangle correction and the pair-wise Bayesian method [9]. These two methods give consistent results, but the triangle method typically has lower posterior variance, particularly for the short lengths. For a true length of zero, Example 1.6, the triangle method is substantially better (provided the other two lengths are large). The triangle correction improves on pair-wise based inference in the following ways:

- It can infer small lengths with higher confidence, Examples 1.1–1.6. A triplet of fluorophores can thus be utilised to improve inference of a small distance by placing a third fluorophore distant from the two fluorophores of interest (50–200nm). This is because correlations between the lengths confer information on the smallest length. This is demonstrated for the small length |*X*_3_ − *X*_2_| in Examples 1.2–1.6, where the examples with the more distant auxiliary fluorophore *j* = 1 and the more co-linear triangle geometries exhibit the largest benefits.
- It infers the triangle (more generally the polygon) and therefore reconstructs the entire geometry of the labelled structure, including the internal angles. Reconstructing polygons from pair-wise estimates, assuming independence of the inferred lengths, can lead to violations of the triangle inequality (see scatterplots in Fig 3), and more generally polygon based constraints.
- Using three or more fluorophores allows measurement errors to be inferred individually for each fluorophore. For pair-wise methods these are not accessible, as only the error of the difference *X*_*j*_ − *X*_*i*_ can be estimated. This follows since the system of equations $\sigma_{ij;d}^{2}≔\sigma_{j;d}^{2}+\sigma_{i;d}^{2}$ for *j* ≠ *i* ∈ {1, …, *J*} can be solved uniquely for each $\sigma_{i;d}^{2}$ for *J* = 3, but not for *J* = 2 (there are *J* unknowns for $\frac{1}{2}J\left(J-1\right)$ constraints). See Table D in S1 Text parameters of Examples 1.1, 1.2.

#### Testing the two-state model on simulated data

To test the multi-state mixture algorithm, we determined inference accuracy of the states and composition of a mixture of *Z* = 2 triangle states on simulated triangle data (*J* = 3). We demonstrate joint inference from a mixed dataset and a pure dataset. The first has a mixture of the two states, *N*_mix_ = 600 measurements (*Z* = 2 states, *J* = 3 fluorophores), with proportions *p*^(1)^ and *p*^(2)^ = 1 − *p*^(1)^ for the states *ζ* = 1, *ζ* = 2, where state *ζ* = 2 is the minor population (*p*^(2)^ ≤ *p*^(1)^). This data is simulated from the multi-state model of subsection Mixture of multiple states (see supplementary section Simulated data in S1 Text for simulation details). The second dataset is a pure population comprising only state *ζ* = 2. We simulate *N*_inform_ = *p*^(1)^ ⋅ *N*_mix_ from the single state model for the pure dataset. Thus, there are the same number of measurements in the pure dataset as measurements of the dominant *ζ* = 1 state in the mixed dataset.

We impose an additional prior on the *ζ* = 1 state to limit exploration of the parameter space during burnin; this reduces the convergence time. Specifically, we use the prior from Eq (AF) in S1 Text. The parameters $l_{ij;0}^{\left(1\right)}$ and $\sigma_{j;d;0}^{\left(1\right)}$ are chosen so the means of the priors are equal to the true values of the simulated data. We confirmed that the dimensions of the support of the prior are sufficiently large so that the prior has negligible impact on the posterior. Specifically, the bulk of the marginal length posteriors are well within the boundaries of the prior support (in fact, in all our examples, the weight of the Gaussian distribution fitted to the joint posterior of the three lengths that was outside the prior support never exceeded 5%). However, the priors on the measurement errors are not weak and can increase confidence on less informative datasets.

**Fig 4:**
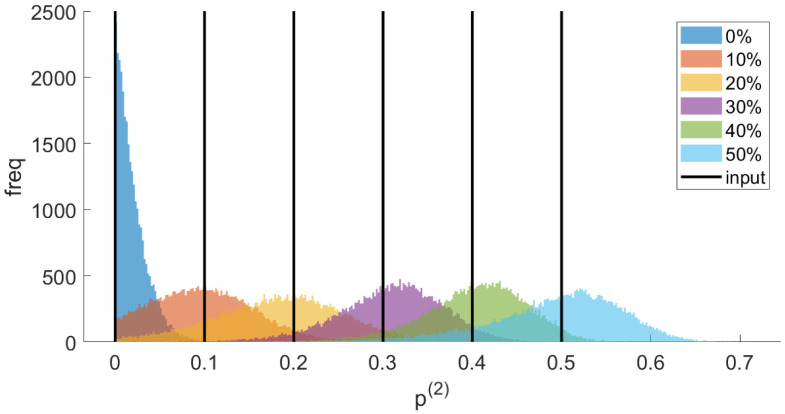
Two-state simulated examples. Posterior state proportions for the simulated two-state model with varying state proportions *p*^(2)^. Original (true) values are marked with black vertical lines, posteriors of each simulated dataset indicated in legend. The prior on the state proportions is flat in [0, 1] in each case.

Simulated examples and the corresponding inferred parameters (triangle lengths and state proportions) are summarised in Table 2. The (marginal) posteriors for the state proportion *p*^(2)^ are shown in Fig 4, demonstrating clear localisation of the posterior around the true value with posterior standard deviations of the order of 6% (for this number of measurements and these parameter values). Thus, the state proportion posteriors are only well separated from zero for true proportions above 10%; for 20% and above there is clear evidence for the presence of the minor population. All lengths are inferred correctly, except the true values of two lengths in Example 2.2 lie in the posterior tail. Since we are inferring 42 parameters in Table 2, it is expected that some deviations will occur. Reruns of Example 2.2 on new datasets gave posteriors consistent with the true values. Inferred measurement errors (omitted for brevity) were all consistent with the true values (for both *ζ* ∈ {1, 2}: $\sigma_{1;xy}^{\left(\zeta \right)}=\sigma_{2;xy}^{\left(\zeta \right)}=10\text{nm}$, $\sigma_{1;z}^{\left(\zeta \right)}=\sigma_{2;z}^{\left(\zeta \right)}=20\text{nm}$ and $\sigma_{3;xy}^{\left(\zeta \right)}=15\text{nm}$, $\sigma_{3;z}^{\left(\zeta \right)}=30\text{nm}$).

**Table 2:** Two-state simulated examples (anisotropic measurement error).

(lengths and errors in nm)	$\left|X_{2}^{\left(1\right)}-X_{1}^{\left(1\right)}\right|$	$\left|X_{3}^{\left(1\right)}-X_{1}^{\left(1\right)}\right|$	$\left|X_{3}^{\left(1\right)}-X_{2}^{\left(1\right)}\right|$	$\left|X_{2}^{\left(2\right)}-X_{1}^{\left(2\right)}\right|$	$\left|X_{3}^{\left(2\right)}-X_{1}^{\left(2\right)}\right|$	$\left|X_{3}^{\left(2\right)}-X_{2}^{\left(2\right)}\right|$	*p* ^(2)^
Example 2.1:							
true value	45	85	45	50	60	15	0%
triangle, Eq (AF) in S1 Text prior	45.7±0.9	85.3±1.1	45.1±1.3	50.7±0.9	60.6±1.0	17.1±2.1	(1.99±1.74)%
Example 2.2:							
true value	45	85	45	50	60	15	10%
triangle, Eq (AF) in S1 Text prior	46.1±1.0	85.6±1.7	44.8±2.1	52.0±0.8*	63.5±1.1*	18.9±2.1	(9.95±5.45)%
Example 2.3:							
true value	45	85	45	50	60	15	20%
triangle, Eq (AF) in S1 Text prior	46.0±1.1	83.3±2.3	41.7±3.3	51.0±0.9	61.3±1.2	18.1±2.1	(18.4±7.1)%
Example 2.4:							
true value	45	85	45	50	60	15	30%
triangle, Eq (AF) in S1 Text prior	43.7±1.4	88.0±2.6	48.0±3.3	50.0±1.0	59.8±1.3	14.7±2.6	(30.8±6.0)%
Example 2.5:							
true value	45	85	45	50	60	15	40%
triangle, Eq (AF) in S1 Text prior	44.4±1.4	89.1±2.9	49.5±3.4	49.8±1.1	59.6±1.3	14.1±2.7	(40.3±6.0)%
Example 2.6:							
true value	45	85	45	50	60	15	50%
triangle, Eq (AF) in S1 Text prior	45.0±1.6	88.5±3.9	48.0±4.8	49.7±1.1	59.8±1.4	14.3±2.7	(49.5±7.5)%

Rows are the true values and posterior means and standard deviations for respective examples, columns the two triangle states (lengths) and the state proportion *p*^(2)^ in the mixture. Note, Example 2.2 has posterior means significantly different from the true value by more than two standard deviations, denoted by * (*p* = 0.8% and *p* = 0.2% for the inferred versus the true values of $\left|X_{2}^{\left(2\right)}-X_{1}^{\left(2\right)}\right|$ and $\left|X_{3}^{\left(2\right)}-X_{1}^{\left(2\right)}\right|$, respectively).

### Analysis of the human kinetochore: A structured macro-molecular complex

#### The experimental system: Human cell division (mitosis)

**Fig 5:**
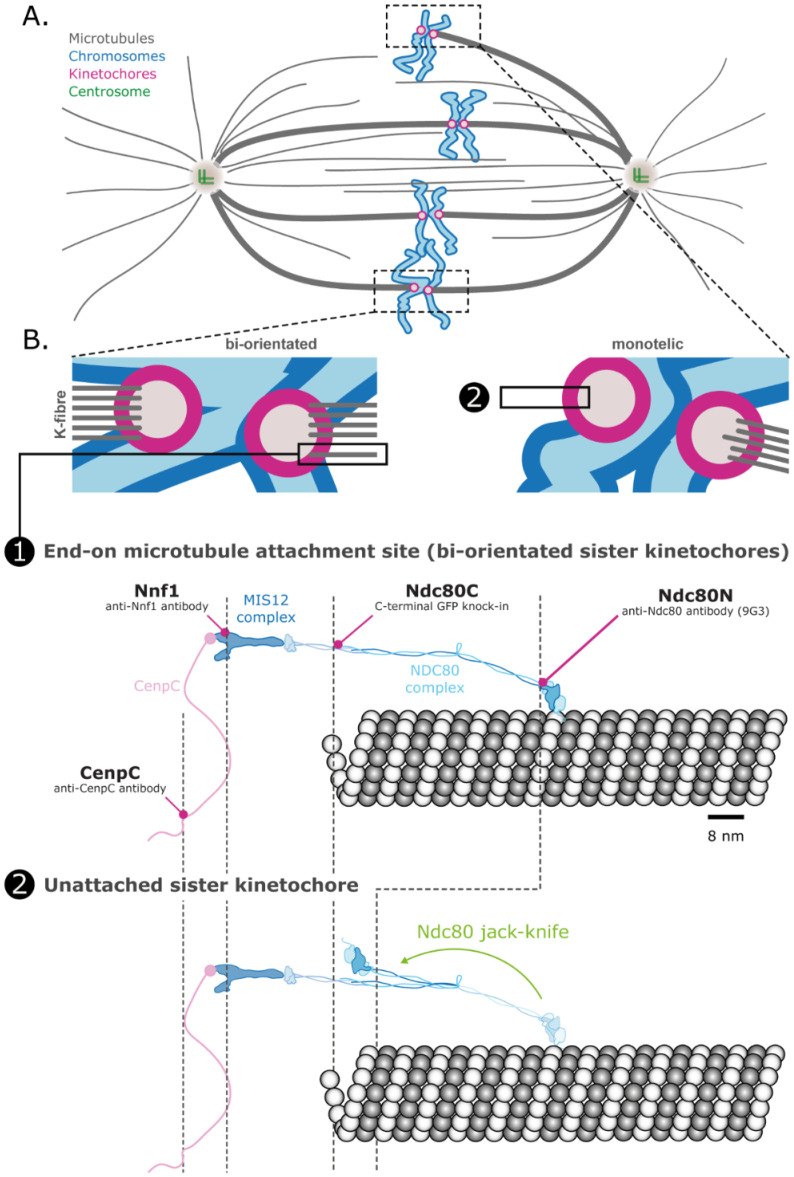
Spindle and kinetochore organisation. **A**. Schematic of the spindle and chromosome attachment to the spindle poles. Chromatids are bi-orientated with microtubule attachments to both spindle poles, except the top chromatid pair where only one kinetochore is attached to a spindle pole (termed monotelic). **B**. Detail of monotelic and bi-orientated sister pairs. The kinetochores are in states 1. (attached) or 2. (unattached), shown below. State 1 (attached). Molecular detail of the Ndc80 complex showing its attachment to the side of a microtubule at its N-terminal binding site. The Ndc80 is attached to the Mis12 complex at its C-terminus. Nnf1, a component of the Mis12 complex, binds CenpC through an unstructured linkage. State 2 (unattached). The Ndc80 complex is hinged—in the unattached state the Ndc80 complex jack-knifes bringing the N and C termini closer together. A kinetochore has about 200–250 Ndc80 complexes and is bound to around 20 microtubules in mature attachment. The approximate positions of the four fluorophores discussed in this paper are shown in purple. Analysis of the geometry of the three triplets CenpC–CenpC–CenpC, CenpC–Ndc80C–Ndc80N and Nnf1–Ndc80C–Ndc80N is given in Tables 3 and 4. Molecules (Ndc80, Mis12 and tubulin heterodimer) are shown approximately to scale from structural biology considerations. Scale bar is 8nm.

To demonstrate our algorithms on experimental data we analysed three-fluorophore, 3D images of human kinetochores from hTERT-immortalised retinal pigment epithelial (RPE) cells, imaged during prometaphase and metaphase with a confocal spinning-disk microscope, [12]. Kinetochores are macro-molecular complexes that orchestrate chromosome movements during cell division, playing a vital role in congression and segregation dynamics [25]. Kinetochores interface between the microtubule-based mitotic spindle and chromosomes, binding to both DNA (through the histone CenpA) and the tips of multiple microtubules (≈ 20 per kinetochore in human cells, [26]), and thus connecting chromosomes to the spindle machinery. They are multi-functional, being both force generators but also sensing erroneously connected microtubules [27]. During S-phase of cell division, chromosomes are replicated, producing two sister chromatids with each assembling a single kinetochore. The sisters are held together, attached near the kinetochores, giving the familiar ‘X’ shape of mitotic chromosomes. At the start of mitosis, after nuclear envelope breakdown, kinetochores are not bound to microtubules. As mitosis progresses, kinetochores attach to microtubules emanating from the spindle poles, and ideally sister kinetochores attach to opposite poles to form a bi-orientated state, Fig 5. Bi-orientated chromosomes congress to the equatorial plane of the mitotic spindle, which is in essence a holding configuration, whilst any remaining chromosomes are captured, bi-oriented and congressed, Fig 5. Any unattached kinetochores activate the spindle assembly checkpoint (SAC) that generates a “wait” signal that delays the onset of chromosome segregation. Only when sufficient microtubule occupancy is achieved will the SAC be silenced, allowing cells to enter anaphase and sister chromatids segregate to separate daughter cells, [28].

A key constituent of kinetochores is the Ndc80 complex that binds to microtubules, Fig 5B. The Ndc80 complex has a hinge about 16nm from the N terminus, which allows the complex to be either fully straightened or folded back (jack-knifed). This conformational change was measured upon microtubule binding, [12]; specifically, the microtubule binding site (N terminus, denoted Ndc80N) moved ≈ 25nm within the kinetochore between microtubule binding conditions (attached and bi-oriented, metaphase cells) and depolymerised microtubule conditions (unattached, nocodazole treatment), Fig 5B, states 1. and 2. The Ndc80 complex thus goes from a jack-knifed, or folded configuration, when unattached, to a straightened state when attached, [12]. Relative to a 3rd fluorophore, we thus have two distinct triangular states. These triangles were reconstructed from an analysis using BEDCA in [12]. By analysing three-fluorophore data with our triangle correction algorithm, the triangle states associated with attached and unattached can be determined directly.

The kinetochore comprises multiple copies of constituent proteins; there are an estimated 200–250 Ndc80 molecules ([29]) and it attaches a bundle of around 20 microtubules [26]. As discussed in [12], our results refer to this ensemble, which is a diffraction-limited spot in each channel. However, the kinetochore is highly structured, with high order when attached. This high ordering within the kinetochore means that the changes in the triangular state reflect a conformational change of the Ndc80 molecule itself, although the unattached conformation is less ordered. The jack-knifed state therefore cannot be mapped quantitatively to a structural closed conformation model. If this ensemble can be resolved, more detailed statements might be possible, [30].

As mitosis progresses and each sister chromatid pair achieves bi-orientation, the cell’s 46 kinetochores change from unattached (in early prometaphase) to all attached at the end of metaphase, immediately before anaphase. Hence, prometaphase cells have a mixture of attached and unattached kinetochores, whilst there could be a minor proportion of kinetochores in metaphase that are still in the unattached state, [31]. However, there is no direct evidence of this conformation change occurring during cell division, or confirmation that the kinetochore population is conformationally heterogeneous. The presence of unattached kinetochores can be inferred by measuring if checkpoint proteins (such as Mad, Bub) are present on individual kinetochores, [32]. However, the checkpoint is downstream of attachment, [33], so is only an indirect indicator of attachment state. Here, we use our mixture model to determine if we can detect conformational heterogeneity in early and late prometaphase and metaphase, and estimate the mixture proportions.

#### Experimental dataset analysis with single-state model algorithm

We consider five examples of triangular fluorescence data from [12] for marked human kinetochore proteins, analysing three fluorophore triplets amongst the four fluorophores shown schematically in Fig 5. These include fluorophores near the N and C termini of the Ndc80 complex, a fluorophore on Nnf1, a sub-unit of the Mis12 complex, which binds Ndc80, extending its rigid arm, and CenpC. There are two conditions—standard growth conditions (DMSO) and under nocodazole treatment where microtubules are fully depolymerised; kinetochores are thus in an unattached configuration. We applied an additional quality control step on the data over that employed in [12], requiring that for both sister kinetochores all three fluorophores were measured and that there were at least ten sister pairs in each cell. This was found to reduce the biological variation within an experiment. The same experimental datasets were analysed in [12] with BEDCA; results are similar to the pair-wise results presented here—differences arise because of the extra quality-control filter and the weaker priors used on the measurement errors in this paper (see supplement Implementation of pair-wise correction methods in S1 Text).

**Fig 6:**
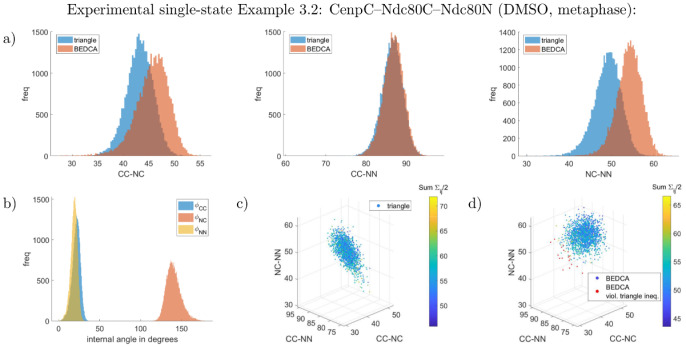
Marginal posteriors of the CenpC–Ndc80C–Ndc80N experiment in DMSO treatment (Example 3.2). Panels are the same as in Fig 3. All lengths are given in nm and have flat priors. See supplement Uninformative priors in S1 Text for the joint priors. Constructing a joint distribution from the three pair-wisely inferred lengths assuming independence yields 1% violations of the triangle inequality (red dots in panel d)).

**Fig 7:**
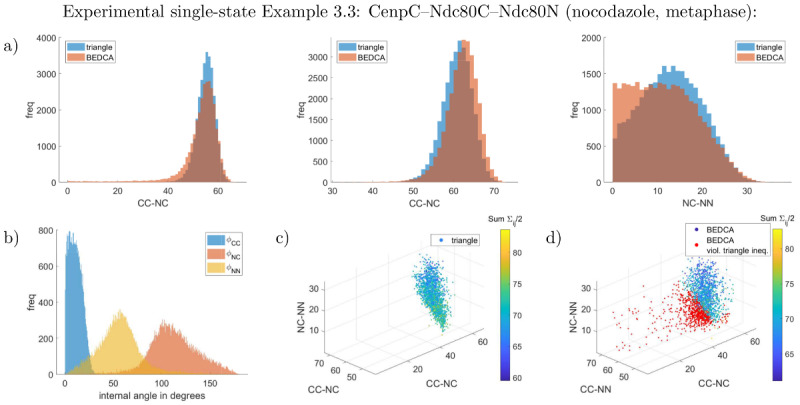
Marginal inferred posteriors of the CenpC–Ndc80C–Ndc80N experiment in nocodazole treatment (Example 3.3). Panels are the same as in Fig 3. All lengths are given in nm and have flat priors. See supplement Uninformative priors in S1 Text for the joint priors. Constructing a joint distribution from the three pair-wisely inferred lengths assuming independence yields 39% violations of the triangle inequality (red dots in panel d)).

**Fig 8:**
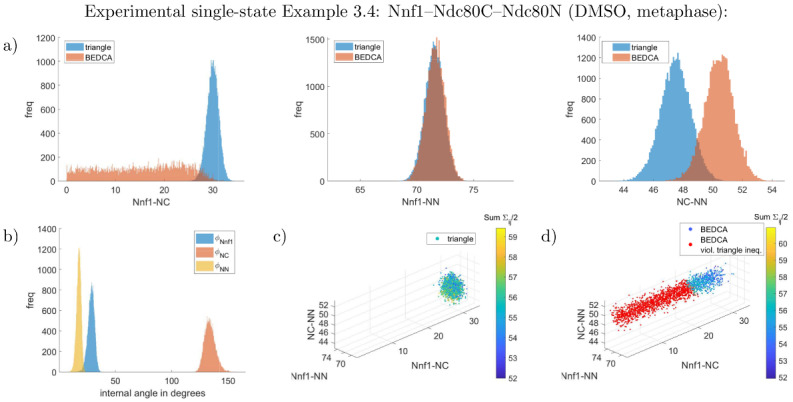
Marginal inferred posteriors of the Nnf1–Ndc80C–Ndc80N experiment in DMSO treatment (Example 3.4). Panels are the same as in Fig 3. All lengths are given in nm and have flat priors. See supplement Uninformative priors in S1 Text for the joint priors. Constructing a joint distribution from the three pair-wisely inferred lengths assuming independence yields 71% violations of the triangle inequality (red dots in panel d)).

We tested our method on experimental data in three ways: i. confirming zero length is inferred for proteins that are triple labelled (the CenpC–CenpC distance, Example 3.1), ii. comparing consistency for the posterior length that is common to different triplets (specifically Ndc80C–Ndc80N), iii. comparing with the pair-wise method of [9], see Table 3 as well as Figs 6–8, L and M in S1 Text. Firstly, for the triple-labelled CenpC experiment, Example 3.1, all three lengths of the triangle have posterior marginals that are against the boundary (zero), practically identical to the pair-wise algorithm (see Fig L in S1 Text), giving a resolution of 2–3nm (although this depends on the number of measurements). Since all three lengths are small here, there is no benefit in using three fluorophores. Secondly, the distance between Ndc80C and Ndc80N is labelled in two triangles with both DMSO and nocodazole treatments, allowing two comparisons. The Ndc80N–Ndc80C agree in both DMSO (Examples 3.2, 3.4, *p* = 30%, posteriors are given in Figs 6 and 8), and nocodazole (Examples 3.3, 3.5, *p* = 48%), posteriors in Fig 7, M in S1 Text); see supplement Computation of p values in S1 Text for definition of *p*. Thirdly, the results from the triangle correction are typically consistent with the pair-wise correction method of [9], Table 3. Exceptions are the Nnf1–Ndc80C length (*p* = 1.0%) and the Ndc80C–Ndc80N length (*p* = 1.9%) in the DMSO-treated Nnf1–Ndc80C–Ndc80N triple, showing a weakly significant difference, see Example 3.4. Examining the posteriors, Fig 8, reveals that the short Nnf1–Ndc80C length from the pair-wise algorithm is approximately flat between 0nm and 27nm, giving distance estimate (16.2 ± 8.4) nm. In contrast, the triangle correction’s length posterior is approximately Gaussian, mean (30.0 ± 1.2)nm, with substantially smaller posterior variance. The tighter inference of the Nnf1–Ndc80C distance by the triangle correction is a consequence of length correlations within the inferred triangle. Generating triangles from the pair-wise marginal length posteriors, assuming independence, results in substantial violation of the triangle inequality, Fig 8d. In fact, even the pair-wise posterior means violate the triangle inequality in this case, indicating it is not possible to construct a joint triangle distribution that preserves the pair-wise length marginals (see Triangle length means never violate the triangle inequality in S1 Text for why no triangle distribution exists that has (marginals with) means violating the triangle inequality). This improvement for smaller lengths is also seen for Ndc80N–Ndc80C in nocodazole, the posterior under the triangle correction has a substantial peak whilst the pair-wise correction has a thick tail towards zero, Fig 7. This is analogous to the thick tails seen in simulated data for small length inference.

**Table 3:** Single-state analysis of experimental datasets.

Example 3.1, CenpC–CenpC–CenpC, *N* = 1336, *C* = 34:			
(lengths and errors in nm)	CenpC–CenpC	CenpC–CenpC	CenpC–CenpC
BEDCA, [9]	2.9±1.9	4.3±2.8	4.5±2.9
triangle, uninformative prior	3.1±2.0	4.1±2.7	4.5±2.7
Example 3.2, CenpC–Ndc80C–Ndc80N (DMSO), *N* = 72, *C* = 3:			
(lengths and errors in nm)	CenpC–Ndc80C	CenpC–Ndc80N	Ndc80C–Ndc80N
BEDCA, [9]	45.7±3.2	86.7±2.5	54.2±2.9
triangle, uninformative prior	43.2±2.6	86.5±2.6	49.0±3.2
Example 3.3, CenpC–Ndc80C–Ndc80N (nocodazole), *N* = 118, *C* = 5:			
(lengths and errors in nm)	CenpC–Ndc80C	CenpC–Ndc80N	Ndc80C–Ndc80N
BEDCA, [9]	53.1±8.1	62.1±3.9	11.6±7.2
triangle, uninformative prior	55.1±3.5	60.9±3.6	13.1±6.7
Example 3.4, Nnf1–Ndc80C–Ndc80N (DMSO), *N* = 570, *C* = 18:			
(lengths and errors in nm)	Nnf1–Ndc80C	Nnf1–Ndc80N	Ndc80C–Ndc80N
BEDCA, [9]	15.2±8.3	71.6±0.8	50.4±1.0
triangle, uninformative prior	30.0±1.2	71.5±0.8	47.5±1.0
Example 3.5, Nnf1–Ndc80C–Ndc80N (nocodazole), *N* = 238, *C* = 8:			
(lengths and errors in nm)	Nnf1–Ndc80C	Nnf1–Ndc80N	Ndc80C–Ndc80N
BEDCA, [9]	15.3±9.3	21.0±11.4	12.7±7.7
triangle, uninformative prior	16.2±8.4	16.8±9.5	12.6±7.4

**Fig 9:**
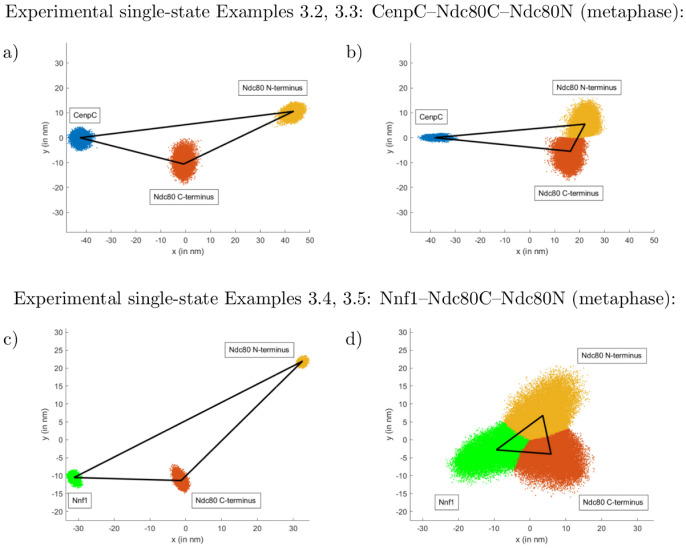
“Average” inferred triangles (Examples 3.2–3.5). The left panels (a,c) are DMSO, the right panels (b,d) nocodazole treated, respectively. The conformational change under nocodazole treatment is clearly visible. The “average” triangles (black lines) are calculated by choosing the perspectives and lengths of the “average” triangle to minimise the Euclidean distance to all triangle templates {*X*_*j*_}_*j*_ sampled via our MCMC. The point-clouds show the nodes of the triangle template samples for these optimised perspectives (relative to the fixed “average” triangle in the *x*-*y*-plane as depicted). The lines connecting these nodes are omitted for better visibility. This is an alternative representation of panels c) in Figs 6–8, M in S1 Text.

Examples 3.1–3.5 with fluorophore labels, number of kinetochores (*N*) and number of cells (*C*). All datasets are from cells in metaphase. Means and standard deviations of the inferred length posteriors are given, comparing the triangle inference presented in this paper with the pair-wise method of [9]. The pair-wise and triangular algorithms were run on the same dataset; the pair-wise results differ slightly to those reported in [12] because of differences in the priors. For the number of kinetochores and cells, see Table E in S1 Text. For a graphical depiction of these kinetochore conformations in DMSO and nocodazole treatment see Fig 9.

The higher confidence of the triangle correction on small lengths reveals that the Nnf1–Ndc80C distance likely increases to twice its length in DMSO compared to nocodazole (Examples 3.4, 3.5). This suggests a possible gain of alignment/order, i.e. the ensemble of Ndc80-Nnf1 complexes increase their alignment with each other upon kinetochore attachment to microtubules (see [12] for further discussions).

Triangle correction allows analysis of the measurement errors of each fluorophore individually—see supplementary Table F in S1 Text for the means and standard deviations of the inferred marginal posteriors. Results are consistent between the examples, if we look at the same fluorophores, protein sites and treatments. In this data, the CenpC fluorophore (in Examples 3.2, 3.3) exhibits the smallest error, having about half the measurement error of some of the other fluorophores. Treatment can also have an effect on the measurement errors. This might be due to variation in fluorophore density, e.g. via changes in antibody accessibility or flexibility of the antibody linker.

#### Experimental dataset analysis with multi-state model algorithm

Here we analyse conformational state heterogeneity of RPE1 experimental datasets. We examine a dataset (generated for this study; see supplementary section Experimental methods: prometaphase-metaphase dataset in S1 Text) with both prometaphase and metaphase cells. We also reexamine the datasets of Table 3 to determine if there is evidence of kinetochore heterogeneity within each dataset.

We generated a triple fluorophore dataset, CenpC–Ndc80C–Ndc80N, that comprises a mixture of metaphase (*N*_meta_ = 570), late prometaphase (*N*_late prometa_ = 134) and early prometaphase cells (*N*_early prometa_ = 302), Example 4.1, Table 4. The cells were classified as early prometaphase, late prometaphase or metaphase from the degree to which the metaphase plate has formed, supplementary section Experimental methods: prometaphase-metaphase dataset in S1 Text). Consistent with previous reports, [34] we observe increased sister-sister distance *l*_*ss*_ (here the CenpC–CenpC distance between sister kinetochores) through prometaphase to metaphase, and a decreasing metaphase plate width from late prometaphase to metaphase, supplementary Table H in S1 Text. From prometaphase to metaphase, the alignment of the sister-sister axis with the metaphase plate normal increases and intrakinetochore swivel decreases, as observed in HeLa cells [35]. Intrakinetochore swivel, *ϑ*, refers to the angle between the Ndc80N–CenpC axis and the CenpC–CenpC sister-sister axis.

**Table 4:** Two-state experimental examples.

Example 4.1, CenpC–Ndc80C–Ndc80N (DMSO, prometaphase-metaphase), *N*_meta_ = 570, *C*_meta_ = 17, *N*_late prometa_ = 134, *C*_late prometa_ = 4, *N*_early prometa_ = 302, *C*_early prometa_ = 10:									
	state 1:			state 2:					
(lengths and errors in nm)	CC–NC	CC–NN	NC–NN	CC–NC	CC–NN	NC–NN	$p_{\text{meta}}^{\left(2\right)}$	$p_{\text{late prometa}}^{\left(2\right)}$	$p_{\text{early prometa}}^{\left(2\right)}$
triangle, uninformative prior	45.4±1.8	86.7±1.3	45.2±1.4	15.8±9.2	21.9±13.8	13.6±8.2	(2.25±2.12)%	(12.5±8.6)%	(88.7±8.2)%
Example 4.2, CenpC–CenpC–CenpC (metaphase), *N* = 1336, *C* = 34:									
	state 1:			state 2:					
(lengths and errors in nm)	CC–CC	CC–CC	CC–CC	CC–CC	CC–CC	CC–CC	*p* ^(2)^		
triangle, uninformative prior	5.4±3.2	6.1±3.7	5.1±3.2	7.3±4.7	9.8±6.6	9.4±5.8	(17.7±4.4)%		
Example 4.3, Nnf1–Ndc80C–Ndc80N (DMSO, metaphase), *N* = 570, *C* = 18:									
	state 1:			state 2:					
(lengths and errors in nm)	Nnf1–NC	Nnf1–NN	NC–NN	Nnf1–NC	Nnf1–NN	NC–NN	*p* ^(2)^		
triangle, uninformative prior	29.9±1.4	69.6±1.0	47.2±1.3	40.3±6.8	84.7±5.6	52.1±6.0	(20.3±8.8)%		

**Fig 10:**
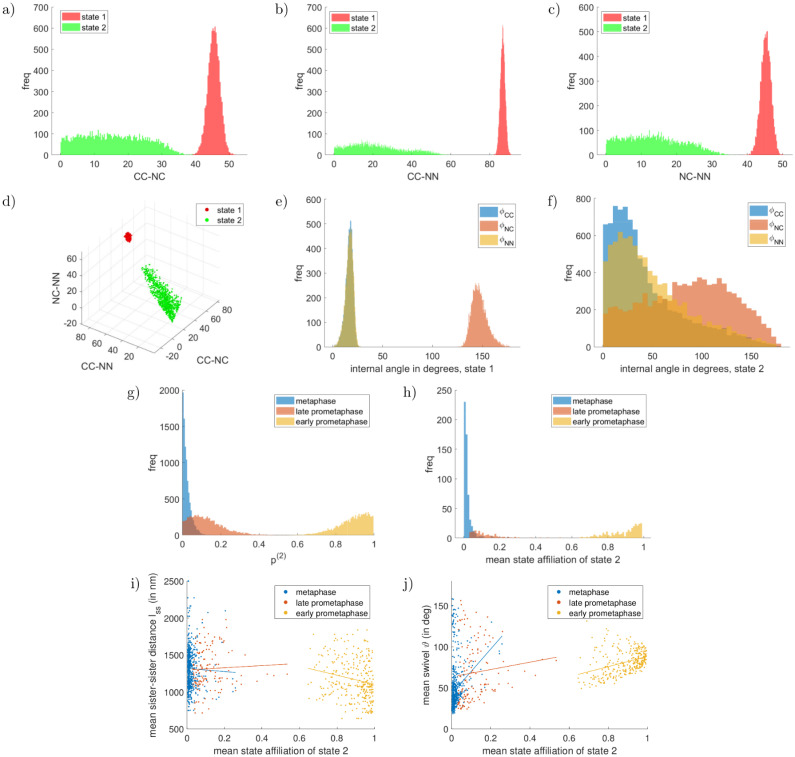
Two-state example for experimental prometaphase-metaphase dataset, Example 4.1. Marginal lengths (in nm) in panels a)–d), the majority state in metaphase is depicted in red, the minority state in green. See supplement Uninformative priors in S1 Text for the joint prior on the lengths. Marginals of the internal angles of the two states are shown in panels e), f). The marginal state proportions of the metaphase-minority state is depicted for each mitotic phase in panel g). The prior on each state proportion is flat in [0, 1]. The mean state affiliations ${\left\{{\bar{\zeta }}^{n}\right\}}_{n}$ for each mitotic phase is shown in panel h), exhibiting clearly separated preferences for kinetochores in metaphase, late prometaphase and early prometaphase. The shown mean state affiliations are with respect to the natural unattached state, i.e. kinetochores with a value close to one are in the natural unattached state. The bottom row compares the inferred mean state affiliations of each kinetochore with the tension parameters: Panel i) shows the mean sister-sister distance *l*_ss_, panel j) the mean swivel *ϑ*, where the mitotic phase is colour-coded and the straight lines show the best fit linear model for each phase. For significance tests, see Table K in S1 Text.

Inference of two states in a mixed state population using experimental data. Inferred posterior means and standard deviations of the triangle lengths of the two states are shown, as well as the state proportions. Abbreviations are CC for CenpC, NC for Ndc80C and NN for Ndc80N. Example 4.1 contains both prometaphase and metaphase cells (a-priori designated), where state 2 is prevalent in prometaphase and shows a jack-knifed conformation distinct from the nocodazole-treated triangle (Example 3.3). Examples 4.2, 4.3 are the same datasets as Examples 3.1, 3.4 analysed with the two-state algorithm. For a graphical depiction see Figs 10 and 13. For the corresponding measurement errors ${\left\{\sigma_{j;d}^{\left(\zeta \right)}\right\}}_{j,d,\zeta }$, see supplementary Table G in S1 Text. For number of kinetochores and cells, see supplementary Table E in S1 Text.

**Fig 11:**
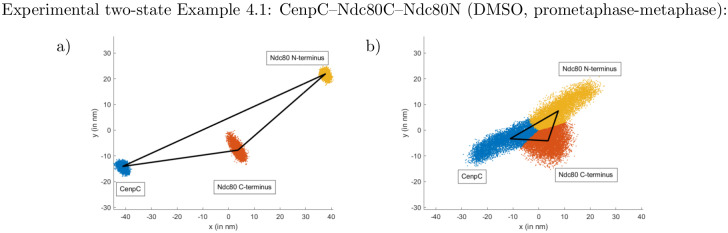
“Average” inferred triangle conformations of Example 4.1. The left panel is the attached state 1, the right panel the natural unattached state 2. Plots are an alternative representation of panel d) in Fig 10 and generated as Fig 9. One can see the clear conformational change between attached and naturally unattached state, whilst the latter is clearly distinct from the nocodazole state in Fig 9, panel b.

We split the data into three subsets based on this mitotic phase designation and jointly analysed these phase subsets using our multi-state algorithm ((mitotic) *phase-based* analysis). The two-state model was run with separate, independent state proportions for the three subsets, ${\left\{p_{\text{meta}}^{\left(\zeta \right)}\right\}}_{\zeta }$, ${\left\{p_{\text{late prometa}}^{\left(\zeta \right)}\right\}}_{\zeta }$, ${\left\{p_{\text{early prometa}}^{\left(\zeta \right)}\right\}}_{\zeta }$, while the other state-dependent parameters (template positions and measurement errors) remain shared across all subsets. The priors on the state-proportions are flat, *i.e*. there is no prior preference imposed as to which conformation is dominant in any subset. Two triangle states are inferred with high confidence: a dominant conformation in metaphase ($p_{\text{meta}}^{\left(1\right)}=\left(97.7\pm 2.1\right)\%$) and late prometaphase ($p_{\text{late prometa}}^{\left(1\right)}=\left(87.5\pm 8.6\right)\%$) that we refer to as the *attached state*, corresponding to attached kinetochores (similar to the conformational state inferred in DMSO, single-state model Example 3.2), and a distinct second conformational state that dominates in early prometaphase cells ($p_{\text{early prometa}}^{\left(2\right)}=\left(88.7\pm 8.2\right)\%$) that has significantly smaller triangle lengths, see Example 4.1 and Fig 10a–10d. This is similar to the jack-knifed conformational state seen in unattached kinetochores in nocodazole (single-state Example 3.3, see supplementary Table C in S1 Text for a summary.). However, it is distinct from this jack-knifed state, with both Ndc80 termini located closer to CenpC. We refer to this as the *natural unattached state*. The posterior lengths of this unattached state have heavy tails towards zero, Fig 10a–10d; the fact these lengths are small (<40 nm for CenpC–Ndc80C and Ndc80C–Ndc80N) is likely a contributing factor as seen in the behaviour of the single-state model. For a graphical depiction of the attached and natural unattached states, see Fig 11.

The inferred state proportions are tightly localised around a pure state, in each subset touching 0 or 1 (Fig 10g), which is reflected in the preference for the single-state model under a model comparison (see supplementary section Model comparison: Two-state vs single-state model in S1 Text), *p*_two-states;meta_ = (11.6 ± 0.1)%, *p*_two-states;late prometa_ = (23.1 ± 1.5)%, *p*_two-states;early prometa_ = (16.3 ± 1.3)%. A separate run with three possible conformational states, did not detect a third conformational state (see supplementary Fig N in S1 Text).

We next examined if individual kinetochores have clear preference for one state. For each kinetochore *n* we compute the mean state affiliation ${\bar{\zeta }}^{n}$, *i.e*. the proportion of MCMC samples that kinetochore *n* is in a given conformational state. The histogram of the mean state affiliations has clear modes close to zero and one, corresponding to the attached state 1 and the natural unattached state 2, respectively, Fig 10h. In fact the subsets split according to these assignments, with both metaphase and late prometaphase kinetochores having a high probability of being in the attached state, whilst most early prometaphase kinetochores have a high probability of being in the natural unattached state. While the state of some kinetochores remains undecided (ca. 4.5% of all kinetochores), the majority show substantial evidence for the attached or natural unattached states, respectively (i.e. ${\bar{\zeta }}^{n}\leq 0.24$ for metaphase and late prometaphase or ${\bar{\zeta }}^{n}\geq 0.76$ for early prometaphase, using substantial evidence thresholds [36]).

We examined how the inferred state affiliations of individual kinetochores are correlated with macroscopic quantities, such as sister-sister distance *l*_ss_ and the intrakinetochore swivel angle *ϑ*. There is a clear correlation: the natural unattached state has a shorter mean sister-sister distance (correlation −0.245 with *p* = 3.6 × 10^−15^) and larger mean swivel (correlation + 0.488 with *p* = 2.8 × 10^−61^), Fig 10i and 10j. A closer examination reveals that these correlations prevail within each cell phase, when there is enough data to allow a significant statement, indicating our method can provide biologically meaningful state affiliations beyond the cell phase assignments, see Table K in S1 Text. This is consistent with microtubule attachments generating a force across the kinetochore sisters, thereby increasing the sister-sister distance, whilst these forces likely create torque that increases the alignment between the microtubule axis and sister-sister axis. We expect the CenpC–Ndc80N axis to correspond to the microtubule axis as seen in HeLa cells [35].

Since *a-posteriori* the single-state model is preferred separately in each mitotic phase, we can also run the single-state algorithm on each mitotic phase individually and compare inferred distances. The inferred distances are essentially identical between the two-state model on the full dataset, and the single state model restricted to each phase, supplementary Table I in S1 Text. A single-state pair-wise analysis (BEDCA) on each mitotic phase also gives near identical results except for mild deviations in late prometaphase; the two-state algorithm identified this as the most heterogeneous phase.

We also examined if a two-state pair-wise analysis is sufficient for heterogeneity analysis on this dataset. We thus extended the pair-wise algorithm to incorporate two-states. This gave consistent results as regards distances (see supplementary Table J in S1 Text), although the CenpC–Ndc80N distance in the natural unattached state was nearly doubled (but not significantly so). Consistent with the two-state triangle results, the pair-wise model also does not show substantial evidence for a second state in any mitotic phase in any of the lengths. The state proportions are also consistent with those inferred by the triangle algorithm, except for the state proportion inferred on the CenpC–Ndc80C pair in late prometaphase (*p* = 0.2%). Note, however, that in the pair-wise analysis we assume that the state proportions are independent between each of the three individual lengths, which is a principle discrepancy to the triangle model that a priori contradicts the idea of underlying triangle architectures. Thus, combining the pair-wise inferences to a joint architecture has two problems: Firstly, as detailed in the single-state sections, the correlations between the lengths are not known and assuming independence can lead to violations of hard geometric constraints such as the triangle inequality. On this data, when doing so there were 78% violations of the triangle inequality for the naturally unattached state. Secondly, the assumption of independent state proportions is a priori incompatible with the idea that the lengths jointly arise from a common architecture. Therefore, while pair-wise analysis gives consistent results on individual lengths, for multi-fluorophore data analysis a comprehensive model incorporating the entire geometry is advised to avoid inconsistencies in architecture and state proportions. We note that the CenpC–Ndc80N distance seems to be the most informative as regards inferred state as the two-state triangle model state proportions are very similar to those of the two-state pair-wise analysis on CenpC–Ndc80N.

**Fig 12:**
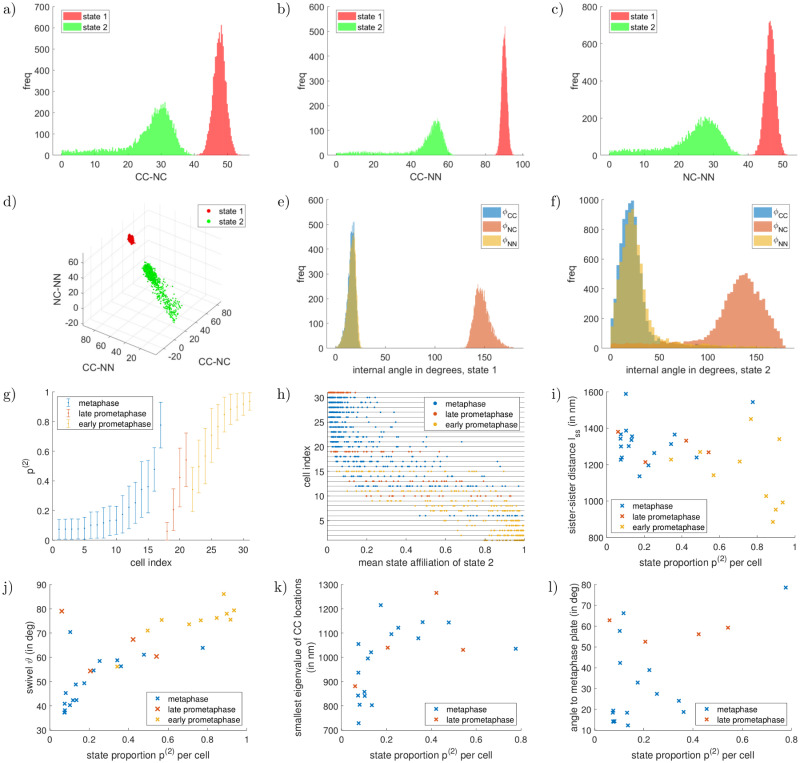
Two-state example for experimental prometaphase-metaphase dataset with cell-based subset analysis, Example C.5. Marginal lengths (in nm) in panels a)–d), the attached state is depicted in red, the natural unattached state in green. See supplement Uninformative priors in S1 Text for the joint prior on the lengths. Marginals of the internal angles of the two states are shown in panels e), f). Panel g) directly shows the means and standard deviations of the inferred state proportions individually for each cell (ordering within the mitotic phase based on mean of *p*^(2)^) and in panel h) each dot denotes the mean state affiliation of a kinetochore in the cell denoted on the vertical axis (ordering based on the mean of *p*^(2)^). Remaining panels compare the inferred state proportion of each cell with tension parameters—each datapoint shows averages of one cell: Shown are the i) sister-sister distance *l*_ss_, j) swivel *ϑ*; k) metaphase plate width (smallest eigenvalue of the CenpC locations); l) angle of the sister-sister axis to the normal of the metaphase plate (eigenvector of the smallest eigenvalue of the CenpC locations), each plotted against the state proportion of the natural unattached state. Mitotic phase is colour-coded. Error bars are suppressed for visibility. Kinetochore-based correlation statistics are shown in Table K in S1 Text.

The analysis with the data split by mitotic phase (early prometaphase, late prometaphase and metaphase) assumes all kinetochores in a subset have the same probability of being in the attached versus unattached conformational state. Since these cells are in various stages of congression, with the degree of attached kinetochores increasing as congression progresses, this may be too strong an assumption. We therefore re-analysed the same data under a different assumption: Instead of splitting the data by mitotic phase, we split the data into 31 subsets, each subset a cell with an independent state proportion, and used the multi-state algorithm to infer two conformational states (*cell-based* analysis). This model thus allows cells to vary, whilst cell phase information is not used in the inference. Investigating both, the phase- and the cell-based analysis, is not intrinsically necessary for our method, but is done to accomodate both modelling ideas. The two inferred states of the cell-based analysis are similar to those inferred with mitotic phase subsets, although in the cell-based inference the natural unattached state is inferred with greater confidence; the intrakinetochore distance posteriors have thinner tails towards zero and a clear mode, Fig 12a–12d, Fig G in S1 Text, and internal angles have lower variance, Fig 12f. However, the posterior variance of the lengths for the triangular states is similar in both analyses, Table C in S1 Text. Cells show a range of state proportions Fig 12g, consistent with the number of attached states increasing from early prometaphase to metaphase, [31, 37]. The state proportions are broader in this cell-based analysis, as expected since they are based on smaller numbers of kinetochore (on average 33.5, 33.5, 30.2 kinetochores per cell for metaphase, late prometaphase and early prometaphase, respectively). The single-state model is preferred in 12 (out of 31) cells across all three mitotic phases, Table L in S1 Text, *i.e*. there is substantial evidence that in these cells the kinetochores are all in the same state. In contrast, 8 cells prefer the two-state model and thus kinetochores in these cells can be in different states, Table L in S1 Text, Fig I in S1 Text. The evidence is not substantial either way in the remaining cells. In cells with intermediate state proportions kinetochores have intermediate mean state affiliations, Fig 12f and Fig J in S1 Text.

The inferred mean state affiliations ${\left\{{\bar{\zeta }}^{n}\right\}}_{n}$ again exhibit clear trends with the tension parameters: kinetochores with high probabilities of being in the natural unattached conformation tend to have smaller sister-sister distances, *l*_ss_ (correlation −0.234 with *p* = 5.3 × 10^−14^), and larger swivels, *ϑ* (correlation + 0.403 with *p* = 1.8 × 10^−40^). See Table K in S1 Text for results for each mitotic phase. The means per cell of the tension parameters show strong correlations with the state proportions *p*^(2)^, correlation coefficient −0.448, *p* = 1.1 × 10^−2^ for *l*_ss_, and +0.794, *p* = 1.0 × 10^−7^ for swivel *ϑ*, Fig 12i and 12j. For the cell-based analysis we can also analyse cell-based statistics for metaphase and late prometaphase cells: The correlation with the metaphase plate width is +0.538, *p* = 1.2 × 10^−2^ and with the angle to the metaphase plate normal, +0.425, *p* = 5.5 × 10^−2^, Fig 12k and 12l. See Table H in S1 Text for definitions. This demonstrates that the conformational state, as inferred by our algorithm, changes with the degree of congression, as evaluated both by eye with the mitotic phase assignment and metaphase plate statistics, as well as local kinetochore forces as measured by tension related statistics.

A second (smaller) experiment also split into early, late prometaphase and metaphase cell subsets gave qualitatively similar results, although in this case, the late prometaphase subset was identified as heterogeneous, i.e. the two-state model was preferred. See supplementary section Experimental dataset analysis with multi-state model algorithm, second experiment in S1 Text.

**Fig 13:**
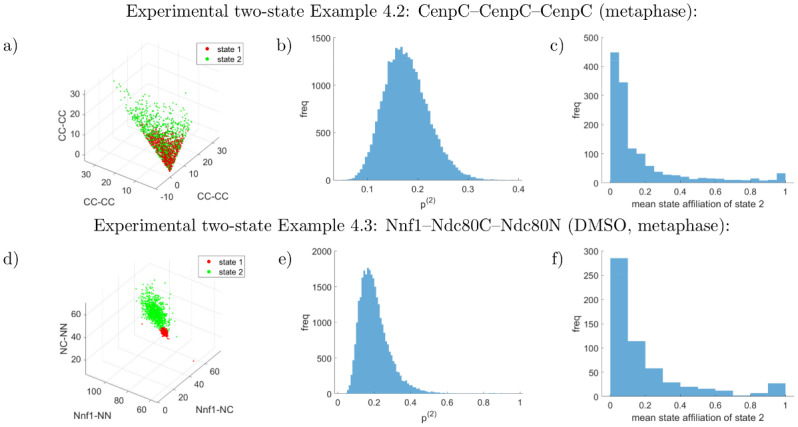
Two-state examples for experimental datasets, Examples 4.2, 4.3. Marginal lengths (in nm) in first column (a,d), the majority state is depicted in red, the minority state in green. See supplement Uninformative priors in S1 Text for the joint prior on the lengths. Marginal state proportions of the minority state are depicted in the second column (b,e). The prior on each state proportion is flat in [0, 1]. The third column (c) shows the mean state affiliations.

We also examined our previous datasets from subsection Experimental dataset analysis with single-state model algorithm with the two-state algorithm, to determine if a second state can be identified. For the CenpC–CenpC–CenpC dataset, Example 3.1, two states are detected but their lengths are not significantly different, Example 4.2. State 2, with a proportion *p*^(2)^ = (18 ± 4)% of the kinetochores, has higher measurement errors, see supplementary Table G in S1 Text. It is not clear what causes this different measurement error. For the DMSO-treated Nnf1–Ndc80C–Ndc80N dataset of Example 3.4, we also detect a second state, Example 4.3. The dominant state is similar to the single-state result, while the minority state with a state proportion *p*^(2)^ = (20 ± 9)% has a significantly larger triangle conformation. In fact the Nnf1–Ndc80C length exceeds the structural constraint of ≲ 30nm between the labelled positions of the Mis12, Ndc80 complexes discussed in [12]. However, Ndc80 : Mis12 stoichiometry is not 1 : 1 which allows the structural constraint to be violated. Kinetochore affiliations again showed bimodality, Fig 13f. An analysis of macroscale measurements did not detect significant correlations. The other datasets from Table 3 did not reveal a second conformation—see supplementary Fig N in S1 Text.

## Discussion

Here we have presented a Bayesian methodology to infer the 3D polygon geometry of *J* ≥ 2 fluorophores within a macro-molecular complex, for fluorophores that localise to distinct regions within the complex and are spot-like when imaged. The algorithm corrects for length and angle bias due to measurement noise, biases that become substantial when polygon sides are of the order of, or less than, the measurement error. Previous correction methods ([6, 8, 9]) are pair-wise methods. Our new method improves on these in four principal ways:

- The full information in multi-fluorophore images is used to infer the polygon, allowing internal angles between the polygon edges and correlations between edge lengths to be inferred. Failure to incorporate the geometry into the inference model results in violations of geometric constraints, such as the triangle inequality, in a number of examples.
- Lengths can be inferred with higher confidence if more than two fluorophores are used. This is particularly beneficial for short lengths (relative to the measurement error), and can overcome inference problems previously reported by [10] (in a single-molecule context). The largest benefits occur when the third fluorophore is located far away and the three fluorophores are approximately co-linear.
- Using three or more fluorophores allows measurement errors to be inferred individually for each fluorophore. This could for example be used for quality control of fluorophores or for quantifying spot expansions (provided the measurement errors remain approximately Gaussian), such expansions potentially being of biological relevance.
- The method is highly flexible. Our algorithm allows for anisotropic measurement errors, typical for 3D imaging, and extends to heterogeneous datasets, inferring both the state conformations and the composition of a mixture of multiple states.

Our algorithm models the full system geometry, which gives it great flexibility, allowing for further generalisations: For instance non-Gaussian measurement errors or anisotropic orientations would be easy to implement. Anisotropy of orientations is likely common in 3D imaging; for example cells may be selected with spindle poles approximately within a focal plane—since kinetochores have a preference to lie along the spindle axis when bi-orientated they are then not likely to be orientated isotropically as a population. Correction of chromatic aberration within the Bayesian inference model would also be possible and allow the variance associated with this effect to be estimated. This flexible approach does however come with longer run-times compared to the pair-wise methods ([6, 9]). The run-time of the single-state simulated Example 1.1 (*N* = 400 measurements and 100, 000 Markov chain iterations) was about 2 days on a standard desktop computer (with enough threads to run five independent chains in parallel). This could be reduced by using state-of-the-art samplers, *e.g*. Hamiltonian Monte Carlo, [38, Ch 9], a re-parametrisation that captures system structural correlations (e.g. utilises the relation in Small lengths are increasingly difficult to infer in S1 Text), or parallelised algorithms [39] (potentially of great benefit since the perspectives, {*T*^*n*^}_*n*_, {*R*^*n*^}_*n*_, are updated independently).

The two-state algorithm enabled us to analyse heterogeneities in the architecture of human kinetochores during mitosis from early prometaphase to metaphase, identifying a natural unattached (jack-knifed) conformation in prometaphase, Figs 10 and 12, Table C in S1 Text. This jack-knifed conformation is distinct from that observed when microtubules are depolymerised (nocodazole treatment); thus a newly assembled kinetochore is in a different state than a kinetochore where microtubules are removed which possibly reflects the change in composition of a kinetochore from prometaphase to metaphase, [40]. The most prominent difference of the prometaphase unattached state is that the Ndc80 is closer to CenpC, Table C and Fig G in S1 Text, possibly reflecting a more collapsed kinetochore, or a result of higher disorder in the kinetochore, [12]. This state corresponds to unattached kinetochores, the dominant state in prometaphase, but since it is an average inferred from a population, there could be minor populations of kinetochores that are partly end-on attached or laterally attached. Further work is required to resolve conformations of these various attachment states.

We demonstrate that kinetochores with Ndc80 straightened out (attached conformation, state 1 in Table C in S1 Text) are under higher tension relative to the natural unattached state, as quantified by the sister-sister distance, Table K in S1 Text, Figs 10i and 12i, consistent with attachment generating forces. The cell-based analysis suggests that congression may be composed of various mechanical phases: tension across sisters is established whilst the attached state proportion is still low, whilst high attached state proportion is associated with both high mean sister-sister tension and reduced mean intrakinetochore swivel, Fig 12i–12l. We hypothesis that to establish sister-sister tension only a fraction of the Ndc80 molecules in a kinetochore need to be attached, too few for the kinetochore ensemble [12] to be in attached state conformation.

We analysed the prometaphase-metaphase dataset under two models for kinetochore heterogeneity, firstly splitting into subsets by mitotic phase and secondly into cell-based subsets. The first model assumes that all kinetochores in cells at a particular mitotic phase have the same probability for being in the attached versus the (natural) unattached conformational state. However, congression, and thus the degree of microtubule attachment, is in various stages of completion in prometaphase. This means that grouping all kinetochores from cells in a given mitotic phase is questionable. The two models gave consistent results for the kinetochore architectures of the two state conformations and both demonstrated correlations between mechanical loading of the kinetochores and the balance between attached versus unattached kinetochores, Figs 10i, 10j and 12i–12l, a loading that increases as congression progresses. However, analysis with mitotic phase subsets suggested that the subsets were essentially pure, each subset preferring a single conformation state, i.e. mitotic phases are essentially homogeneous, Fig 10h. In contrast, the cell-based analysis identified cells with intermediate proportions, Fig 12g, intermediate (uncertain) kinetochore state affiliations, Fig 12h, and the two-state model was preferred in some cells. Thus, in the cell-based model there are heterogeneous cells, in contrast to the homogeneity of the mitotic phases we see in the mitotic-phase-based model. The proportion of the natural unattached state in each mitotic phase can be determined within the cell-based model, by randomly sampling kinetochores in each mitotic phase, giving 17.7 ± 3.6%, 24.0 ± 7.4% and 75.0 ± 5.3% in metaphase, late prometaphase and early prometaphase respectively, see supplement S1 Text for method. These proportions reflect the cell variation seen within a mitotic phase in the cell-based model, Fig 12g and 12h.

In cells with intermediate proportions the cell-based analysis did not confidently assign individual kinetochores to a conformation, Fig J in S1 Text. This reflects the high measurement noise relative to the size of the conformation change, *i.e*. the noise is of the order of the difference in triangle lengths between the two states. This behaviour could also be seen in simulations, while for smaller simulated measurement errors the affiliations could be assigned accurately. Thus, to assign states to individual kinetochores in cells with kinetochore heterogeneity, higher spatial accuracy in the data will be required.

We also detected heterogeneity in a large dataset on the Nnf1–Ndc80C–Ndc80N triple (DMSO, metaphase), Example 4.3, where the Nnf1–Ndc80C length exceeds the structural constraint of ≲ 30nm of the Mis12-/Ndc80 complexes discussed in [12]. We hypothesise that this second state corresponds to a shift in the proportion of Ndc80 complexes bound through the CenpC-Mis12, CenpT-Mis12 or CenpT only linkers, [41]. Further work is needed to clarify this.

Our method allows conformations of macro-molecular complexes to be analysed offering a new technique for study of conformational change in vivo, distinct from conformation (proximity) sensitive readout approaches. These include for example Double Electron-Electron Resonance (DEER; e.g. [42]), Förster Resoncance Energy Transfer (FRET; e.g. [21]) and Bimolecular Fluorescence Complementation (BiFC; e.g. [43]). Our method is a post-processing method for multi-fluorophore image data and does not need a specialised experimental setup or specialised fluorophores as these other methods. Our multi-state algorithm requires a large enough number of measurements to allow for the statistical analysis (in our examples tens to hundreds of measurements) and a discrete number *Z* of distinct (polygon) states. This is particularly important for analysis of mixed populations. For our prometaphase-metaphase examples, joint inference was essential for inferring the states, each subset providing sufficient measurements, and thus sufficient information to define its respective dominant state. Inferred minor states from single heterogeneous datasets, such as the early prometaphase dataset alone, have low confidence, in both the polygon and its proportion. It is likely that the decomposition of heterogeneous populations can be improved by using higher-resolution imaging, e.g. Super-Resolution Structured Illumination Microscopy (SR-SIM, [14]), Super-Resolution Radial Fluctuations (SRRF, [15]) microscopy, or by capturing substantially more photons. Note, we only apply our Bayesian algorithm after fitting the PSF, so the procedure does not depend on the particular shape of the PSF (as long as the same-coloured spots are sufficiently separated). On the other hand, our method works at any distance, and while resolution decreases for shorter lengths, on some examples we get confidences as low as 1nm. This range of tens of nanometres is important at the molecular scale. For instance the conformation change of the kinetochore from attached to unattached state involves a reduction of the CenpC–Ndc80N distance from 85nm to 60nm, which is beyond the scope of DEER or FRET. From this perspective our multi-state algorithm complements these experimental methods, as it bridges the gap between their ≲ 10nm range and the length scale of the PSF, where distance corrections become negligible.

## Supporting information

S1 TextSupplementary methods, including details of the inference (MCMC) algorithm and additional analysis, Supplementary Figures. Supplementary Tables.**Fig A. Numerical simulation of the histograms for three possible priors on the triangle template positions** {*X*_*j*_}_*j*_. Marginal densities are shown on the space of the three triangle lengths (top row; frequency is indicated by colour and radius) and the associated marginal distribution of a single length (bottom row). From left to right the prior distributions are (∏_*j*_ *dX*_*j*_), $\frac{1}{l_{12}\cdot l_{13}\cdot l_{23}}\cdot \left(\prod_{j}\;dX_{j}\right)$, *π*_*X*_[{*X*_*j*_}_*j*_] ⋅ (∏_*j*_ *dX*_*j*_). The support is bounded by the triangle inequality, e.g. the front face is the bounding simplex *l*_23_ = *l*_12_ + *l*_13_. A cut-off for each of the lengths was used, forcing them to be within [0, 1]. The symmetry of the prior with respect to re-labelling fluorophores *j* was used when sampling.**Fig B. Numerical simulation of the histograms for the suggested priors on the template positions** {*X*_*j*_}_*j*_. Marginal densities are shown on the space of the polygon lengths (symmetric for all lengths). A cut-off for each of the lengths was used, forcing them to be within [0, 1]—note that this boundary effect becomes more pronounced for higher dimensions. The symmetry of the prior with respect to re-labelling fluorophores *j* was used when sampling.**Fig C. Posterior distribution for measurement error and length for 3D simulated data**. Posterior based on *N* = 2000 samples simulated with an input (true) length of 15nm and input (true) measurement error of $\sqrt{15^{2}+25^{2}}\text{nm}\approx 29\text{nm}$. Plotted is the likelihood function, Eq (6) from [6]. Probability density is colour coded as key. The red line is Eq (AK) in S1 Text with the second moment (left hand side) estimated from the data.**Fig D. Example cells identified as in early prometaphase (top), late prometaphase (middle) or metaphase (bottom), respectively**. Depicted are the three channels of three example cells of the dataset of Example 4.1. Scale bar is 2*μ*m.**Fig E. Model comparison between two-state and single-state model for Example 4.1**. Dependence of the probability of the two-state model on the prior parameter *α*, as given in Eq (AP) in S1 Text. Panel a) shows the analysis for the metaphase subset, panel b) for the late prometaphase subset, panel c) for the early prometaphase subset. The orange line denotes the mean over the five independent runs, while the shaded area is the ±1*σ* range for each *α*. “Substantial” evidence for a model is typically acknowledged for probabilities ≥ 76% (i.e. Bayes factors ≥ 3.2; [36]).**Fig F. Two-state example for experimental prometaphase-metaphase dataset, second dataset, Example C.4**. Marginal lengths (in nm; pooled over metaphase and prometaphase) in panels a)–d), the majority state in metaphase is depicted in red, the minority state in green. See supplement Uninformative priors in S1 Text for the joint prior on the lengths. Marginals of the internal angles of the two states are shown in panels e), f). The marginal state proportions of the metaphase-minority state is depicted for each mitotic phase in panel g). The prior on each state proportion is flat in [0, 1]. The mean state affiliations ${\left\{{\bar{\zeta }}^{n}\right\}}_{n}$ for each mitotic phase is shown in panel h), exhibiting clearly separated preferences for kinetochores in metaphase and early prometaphase, while late prometaphase contains some kinetochores that are likely attached, some likely unattached and some undecided. The shown mean state affiliations are with respect to the natural unattached state, i.e. kinetochores with a value close to one would be most likely in jthe natural unattached state. The bottom row compares the inferred mean state affiliations of each kinetochore with the tension parameters: Panel i) shows the mean sister-sister distance *l*_ss_, panel j) the mean swivel *ϑ*, where the mitotic phase is colour-coded and the straight lines show the best fit linear model for each phase. For significance tests, see Table K in S1 Text.**Fig G. Comparison of lengths marginals of jack-knifed (nocodazole), naturally unattached and attached states of CenpC–Ndc80C–Ndc80N triangle**. Shown are the length marginals of the CenpC–Ndc80C–Ndc80N triangle in the three conformational states inferred in this study (attached, naturally unattached and jack-knifed), combining results of Examples 3.2, 3.3, 4.1, C.4, and C.5. All lengths are given in nm and have flat priors. See supplement Uninformative priors in S1 Text for the joint priors. Abbreviations are CC for CenpC, NC for Ndc80C and NN for Ndc80N. NAS denotes the (natural) attached state 1 (dominant in metaphase, DMSO), NUS the natural unattached state 2 (dominant in early prometaphase, DMSO) and JKS the jack-knifed state (in nocodazole). The single-state results are shown in blue (Example 3.2, 3.3), red is the two-state phase-based analysis (Example 4.1), yellow the second experiment of the two-state phase-based analysis (Example C.4) and purple the two-state cell-based analysis (Example C.5).**Fig H. A-posteriori estimate of state proportions and affiliations for cell-based experimental prometaphase-metaphase dataset, Example C.5**. Panel a) shows the a-posteriori estimated state proportions for each mitotic phase, ${\tilde{p}}^{\left(2\right)}$ (see subsection Cell-based subset analysis inference of mitotic phase variables in S1 Text for definition). The state proportions are pulled away from the boundaries by the prior. Panel b) shows the mean state affiliations pooled within each of the three mitotic phases. The shown mean state affiliations are with respect to the natural unattached state, i.e. kinetochores with a value close to one would be most likely in natural unattached state. Panel c) shows the numerically estimated prior on the a-posteriori estimates of the state proportions ${\tilde{p}}^{\left(2\right)}$ for our cell-numbers and -sizes in each mitotic phase (same for all states). For the cell-based analysis the prior on the state proportions is independent between cells and flat in [0, 1] for each cell. As we have multiple cells in the same mitotic phase, this means that the prior on the a-posteriori estimates of the state proportions ${\tilde{p}}^{\left(2\right)}$ in a mitotic phase are not uniform anymore, but more concentrated around 50%.**Fig I. Cell-based model comparison of single- vs two-state model for experimental prometaphase-metaphase dataset, Example C.5**. The horizontal axis shows the the cells ordered with respect to their mean intrakinetochore swivel *ϑ*. Mitotic phases are indicated by colour. The vertical axis shows the probability of the single-state model in state 1 and 2, respectively. For a computation of the model comparison see supplementary section Model comparison: Two-state vs single-state model in S1 Text, where we chose *α* = 100% for all cells and states here. The grey shading indicates the region, where substantial evidence for the single-state model (i.e. homogeneous cells with all kinetochores in the same state) is found (i.e. Bayes factor ≥3.2).**Fig J. State proportions and mean state affiliations for example cells for cell-based experimental prometaphase-metaphase dataset, Example C.5**. State proportions of natural unattached state 2 (blue) and mean state affiliations per kinetochore (red) for six cells. Top row shows a cell in metaphase (a), late prometaphase (b) and early prometaphase (c), respectively. Examples of cells in each mitotic phase that have mean state proportions close to 0.5 are shown. In this case ca. ^2^/_3_ of the kinetochores of the respective cells are undecided (i.e. mean state affiliation in [0.24, 076]; no substantial evidence according to [36]). Bottom row shows again one cell in metaphase (d), late prometaphase (e) and early prometaphase (f), where the examples were chosen to illustrate cells in each mitotic phase that have a mean state proportion closest to 0 (or 1 in early prometaphase). In this case almost no kinetochores are undecided.**Fig K. Markov chain traces of each of the parameters** *l*_*ij*_ = |*X*_*j*_ − *X*_*i*_|, *σ*_*i*;*d*_ **(in nm) for the single-state simulated Examples 1.1, 1.2**. Traces are plotted post burnin and sub-sampled to give 10000 samples. Five independent chains are overlain in separate colours.**Fig L. Marginal posteriors of the triple labelled CenpC experiment (Example 3.1)**. Panels are the same as in Fig 3. All lengths are given in nm and have flat priors. See supplement Uninformative priors in S1 Text for the joint priors. Constructing a joint distribution from the three pair-wisely inferred lengths assuming independence yields 62% violations of the triangle inequality (red dots in panel d)).**Fig M. Marginal inferred posteriors of the Nnf1–Ndc80C–Ndc80N experiment in nocodazole treatment (Example 3.5)**. Panels are the same as in Fig 3. All lengths are given in nm and have flat priors. See supplement Uninformative priors in S1 Text for the joint priors. Constructing a joint distribution from the three pair-wisely inferred lengths assuming independence yields 57% violations of the triangle inequality (red dots in panel d)).**Fig N. Multi-state results of experimental examples**. Depicted are the lengths-marginals (in nm) of the multi-state model run on the experimental examples of the main text (subsections Experimental dataset analysis with single-state model algorithm, Experimental dataset analysis with multi-state model algorithm), but containing one more state than the results presented in the main text. Specifically the panels show Example 3.2 (a), Example 3.3 (b), Example 3.5 (c) and Example 4.1 (d), Example 4.2 (e), Example 4.3 (f). State labels are assigned based on prevalence in the datasets, i.e. *p*^(1)^ ≥ *p*^(2)^ ≥ *p*^(3)^. It can be seen that the additional state is not well-informed and spreads over several hundred nanometres. Note that due to our prior to have at least three measurements in each state, the lengths stay localised to some extent. See supplement Uninformative priors in S1 Text for the joint priors on the lengths.**Table A. Number of Markov chain iterations in the examples presented in the main part**. In all examples the first 40% of the iterations is burn-in, and the remaining 60% the posterior samples.**Table B. Single-state simulated example for four fluorophores** (*N* = 400). Rows are the simulated input values and the posterior means ± standard deviations of the four-fluorophore version of our algorithm, respectively.**Table C. Inferred conformations of CenpC–Ndc80C–Ndc80N**. Inference of two states in a mixed state population using experimental data. Inferred posterior means and standard deviations of the triangle lengths of the two states are shown, as well as the state proportions. Abbreviations are CC for CenpC, NC for Ndc80C and NN for Ndc80N. Examples C.1–C.3 are duplicates of Examples 3.2, 3.3 and Example 4.1. Example C.4 shows a second, experimentally equivalent example as Example 4.1. Example C.5 shows the same experimental data to Example 4.1, but split by cell into 31 subsets for the state proportions. Here the ${\left\{{\tilde{p}}^{\left(\zeta \right)}\right\}}_{\zeta }$ denote the a-posteriori estimated state proportions, see subsection Cell-based subset analysis inference of mitotic phase variables in S1 Text. The priors for ${\left\{{\tilde{p}}^{\left(\zeta \right)}\right\}}_{\zeta }$ are not flat as is the case for the mitotic-phase analysis. Posterior distributions for these lengths and states are compared in Fig G in S1 Text.**Table D. Inferred measurement errors *σ*_*j*;*d*_ for single-state simulated Examples 1.1, 1.2 for each fluorophore individually**. Due to an indistinguishability for the pair-wise methods, these parameters can only be inferred, if at least three fluorophores *J* ≥ 3 are used.**Table E. Sizes of experimental datasets**. The number of kinetochores and number of cells are given after image processing and quality control.**Table F. Inferred measurement errors *σ*_*j*;*d*_ for single-state experimental Examples 3.1–3.5 for each fluorophore individually**. Here we abbreviate CC for CenpC, NC for Ndc80C and NN for Ndc80N. The same fluorophores were used across all examples to mark the various structures of the kinetochore (apart from the triple-CenpC, Example 3.1, where A568 is the secondary antibody of the CenpC in the other examples). The table shows consistency of the inference results of the same fluorophore between different examples. Here, the fluorophore for CenpC exhibits the smallest measurement error. Slight differences can be seen for the same fluorophores between DMSO- and nocodazole-treated cells (Nnf1, Ndc80N). Due to an indistinguishability for the pair-wise methods, these parameters can only be inferred, if at least three fluorophores *J* ≥ 3 are used.**Table G. Inferred measurement errors *σ*_*j*;*d*_ for two-state experimental Examples 4.1–4.3 for each fluorophore individually**. Inference of two states in a mixed state population using experimental data. Inferred posterior means and standard deviations of the measurement errors of the two states are shown. Abbreviations are CC for CenpC, NC for Ndc80C and NN for Ndc80N. For Example 4.1 we do not observe a significant difference of the measurement errors between the two states, while for Examples 4.2, 4.3 the two states have significantly different errors, with the minority state 2 typically (in all significant cases) exhibiting a higher measurement error. A comparison between the examples is not clear, because it is not clear how the different states relate to each other.**Table H. Mitotic-phase versus tension parameters for two-state experimental Example 4.1**. † The metaphase plate width is defined as the square root of the smallest eigenvalue of the covariance of the CenpC locations. The corresponding eigenvector is used as the normal of the metaphase plate.**Table I. Inferred conformations of CenpC–Ndc80C–Ndc80N in prometaphase-metaphase, single-state results compared with two-state**. Examples I.1–I.3 are duplicates of Examples 3.2, 3.3 and Example 4.1 (omitting state proportions), Examples I.4–I.6 are single-state results on subsets of the prometaphase-metaphase dataset used for Example 4.1, identified by the mitotic phase of the cells. Means and standard deviations of the inferred length posteriors are given, comparing the single-state triangle inference presented in this paper with the single-state pair-wise method of [9].**Table J. Inferred conformations of CenpC–Ndc80C–Ndc80N in prometaphase-metaphase, two-state pair-wise results compared to two-state triangle**. Example J.1 is a duplicate of Example 4.1, while each row of Example C.2 is based on the same dataset with mitotic-phase subsets as Example 4.1 using two-state pair-wise correction. Inferred posterior means and standard deviations of the triangle lengths of the two states are shown, as well as the state proportions. Abbreviations are CC for CenpC, NC for Ndc80C and NN for Ndc80N. Like for the triangle results on the same dataset, a model comparison (see supplemental section S1) gives no substantial evidence for two states in any mitotic phase—see bottom table. Constructing a joint distribution from the three pair-wisely inferred lengths assuming independence yields 1% and 78% violations of the triangle inequality for state 1 and state 2, respectively.**Table K. Correlations per kinetochore of mean state affiliation and tension parameters for two-state experimental examples**. Provided there is enough data for a significant statement (i.e. *p* ≤ 5 × 10^−2^), there is a positive correlation between the mean state affiliation ${\bar{\zeta }}^{n}$ of a kinetochore and its mean swivel *ϑ* and a negative correlation between its mean state affiliation and its mean sister-sister distance *l*_*ss*_. This correlation is also present in the cell-based subset analysis when information on the mitotic phase is not provided to the multi-state inference algorithm. This indicates that the attached state conformation is associated with a more stretched mechanical condition.**Table L. Cell-based model preference, subdivided by mitotic phase: two-state experimental Example C.5**. The two-state and one-state (either state) models are a-priori equiprobable. Substantial evidence was acknowledged, if the estimated probability of the respective model was significantly (in terms of sampling error) above the threshold of 76%. See supplementary subsection Model comparison: Two-state vs single-state model in S1 Text for details on the model comparison.**Algorithm A**. Recursive algorithm for prior on template positions, *π*_*X*_ [{*X*_*j*_}_*j*_].(PDF)
