# Supplementary material for: Bayesian inference of multi-point macromolecular architecture mixtures at nanometre resolution
Source: PLoS Comput Biol. 2022 Dec 27;18(12):e1010765. doi: 10.1371/journal.pcbi.1010765 (PMC9829179; doi:10.1371/journal.pcbi.1010765)
Supplement: S1 Text — Supplementary methods, including details of the inference (MCMC) algorithm and additional analysis, Supplementary Figures. Supplementary Tables. Fig A. Numerical simulation of the histograms for three possible priors on the triangle template positions {Xj}j. Marginal densities are shown on the space of the three triangle lengths (top row; frequency is indicated by colour and radius) and the associated marginal distribution of a single length (bottom row). From left to right the prior distributions are (∏j dXj), 1l12⋅l13⋅l23⋅(∏jdXj), πX[{Xj}j] ⋅ (∏j dXj). The support is bounded by the triangle inequality, e.g. the front face is the bounding simplex l23 = l12 + l13. A cut-off for each of the lengths was used, forcing them to be within [0, 1]. The symmetry of the prior with respect to re-labelling fluorophores j was used when sampling. Fig B. Numerical simulation of the histograms for the suggested priors on the template positions {Xj}j. Marginal densities are shown on the space of the polygon lengths (symmetric for all lengths). A cut-off for each of the lengths was used, forcing them to be within [0, 1]—note that this boundary effect becomes more pronounced for higher dimensions. The symmetry of the prior with respect to re-labelling fluorophores j was used when sampling. Fig C. Posterior distribution for measurement error and length for 3D simulated data. Posterior based on N = 2000 samples simulated with an input (true) length of 15nm and input (true) measurement error of 152+252nm≈29nm. Plotted is the likelihood function, Eq (6) from [6]. Probability density is colour coded as key. The red line is Eq (AK) in S1 Text with the second moment (left hand side) estimated from the data. Fig D. Example cells identified as in early prometaphase (top), late prometaphase (middle) or metaphase (bottom), respectively. Depicted are the three channels of three example cells of the dataset of Example 4.1. Scale bar is 2μm. Fig E. Model comparison between two-state and single-stat [file pcbi.1010765.s001.pdf]

Bayesian inference of multi-point macromolecular architecture mixtures at nanometre resolution

Peter A Embacher[1], Tsvetelina E Germanova[2], Emanuele Roscioli[2,¤], Andrew D McAinsh[2], Nigel J Burroughs[3,*]

1 Department of Medical Physics & Biomedical Engineering, University College London, London, United Kingdom
2 Centre for Mechanochemical Cell Biology and Division of Biomedical Sciences, Warwick Medical School, University of Warwick, Coventry, United Kingdom
3 Mathematics Institute and Zeeman Institute, University of Warwick, Coventry, United Kingdom

¤Current Address: Toscana Life Sciences Foundation, Siena, Tuscany, Italy
* Corresponding Author: n.j.burroughs@warwick.ac.uk

Supporting information

S1 Text. Supplementary methods, including details of the inference (MCMC) algorithm and additional analysis, Supplementary Figures. Supplementary Tables.

Uninformative priors

We use uninformative (improper) priors, namely:

$$d\pi_0\left[\left\{\theta_j\right\}_j,\left\{\vartheta^n\right\}_n\right]:=$$
$$\pi_X\left(\left\{X_j\right\}_j\right)\cdot\left(\prod_j dX_j\cdot\chi_{\sigma_{j;xy}\geq 0}d\sigma_{j;xy}\cdot\chi_{\sigma_{j;z}\geq 0}d\sigma_{j;z}\right)\cdot\left(\prod_n dT^n\cdot d\pi_R\left(R^n\right)\right),\quad\text{(A)}$$

where χ is the characteristic function, $\pi_X\left[\left\{X_j\right\}_j\right]$ is the prior distribution on the template positions and $d\pi_R\left[R^n\right]$ is the probability measure on the rotation matrices $R^n\in\mathbb{R}^{3\times 3}$ as follows.

Uninformative priors are typically chosen to be flat (uniform); however this is not re-parametrisation independent, and there are many choices of polygon properties where a uniform prior can be imposed. Because our interest is in the polygon side lengths, a flat prior on each length $l_{ij}:=|X_j-X_i|$ would appear appropriate, giving the (improper) prior $\propto\left(\prod_{i\neq j}dl_{ij}\right)$. However, the marginal prior for any length would in fact not be flat for $J>2$, because of the constraints on the lengths to form a J-polygon. Priors with approximately flat marginal densities in the lengths can be constructed for the triangle case ($J=3$) using the density (see Flat prior on marginals of lengths in S1 Text for a derivation):

$$\pi_X\left[\left\{X_j\right\}_j\right]\propto\frac{1}{l_{12}\cdot l_{13}\cdot l_{23}\cdot\min\left(\left\{l_{12},l_{13},l_{23}\right\}\right)}.\quad\text{(B)}$$

Three priors on the template positions are compared in Fig A in S1 Text, namely $\left(\prod_j dX_j\right)$, $\frac{\left(\prod_j dX_j\right)}{l_{12}\cdot l_{13}\cdot l_{23}}$ and $\pi_X\left[\left\{X_j\right\}_j\right]\cdot\left(\prod_j dX_j\right)$.

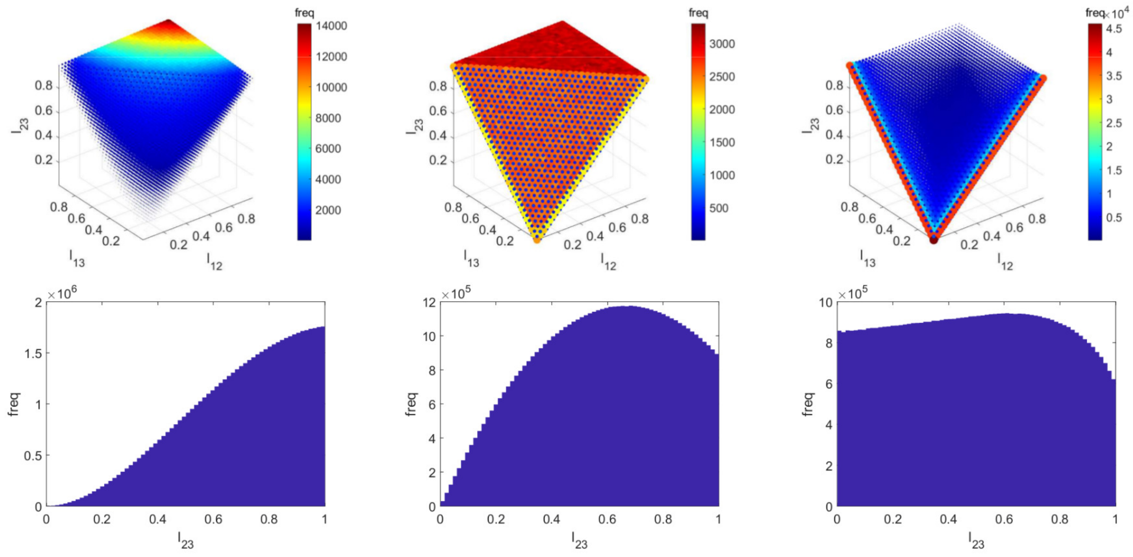

Fig A. Numerical simulation of the histograms for three possible priors on the triangle template positions $\{X_j\}_j$. Marginal densities are shown on the space of the three triangle lengths (top row; frequency is indicated by colour and radius) and the associated marginal distribution of a single length (bottom row). From left to right the prior distributions are $\left(\prod_j dX_j\right)$, $\frac{1}{l_{12}\cdot l_{13}\cdot l_{23}}\cdot\left(\prod_j dX_j\right)$, $\pi_X\left[\{X_j\}_j\right]\cdot\left(\prod_j dX_j\right)$. The support is bounded by the triangle inequality, e.g. the front face is the bounding simplex $l_{23}=l_{12}+l_{13}$. A cut-off for each of the lengths was used, forcing them to be within $[0,1]$. The symmetry of the prior with respect to re-labelling fluorophores j was used when sampling.

The measure $d\pi_R$ on the rotation matrix R^n is defined, such that for an arbitrary vector on the $\mathbb{R}^3$-unit sphere, $v\in\mathbb{S}^3$, the image $R^n\cdot v\cdot d\pi_R\left[R^n\right]$ is uniform on the surface of $\mathbb{S}^3$ (i.e. the Lebesgue measure on that surface, $\sin(\psi)d\psi d\varphi$ in spherical polars).

Flat prior on marginals of lengths

Here we show, that the distribution given in Eq (B) in S1 Text corresponds to a flat prior on the marginals of the lengths in the limit of unconstrained triangle sizes.

Triangles are specified by the three 3D positional vectors X_j, $j\in\{1,2,3\}$, giving the unbounded parameter space $\mathbb{R}^9$. Consider the stripe subspaces,

$$B_{ij;\epsilon}\left(l_0\right):=\left\{\left\{X_{\tilde{j}}\right\}_{\tilde{j}}\middle|\max\left(\left\{l_{\tilde{i}\tilde{j}}\right\}_{\tilde{i}\tilde{j}}\right)<a, l_{ij}\in\left[l_0-\epsilon;l_0+\epsilon\right]\right\}. \tag{C}$$

corresponding to length $l_{ij}:=|X_j-X_i|$ constrained to a small ϵ-neighbourhood of a fixed value $l_0>0$, and all lengths being less than $a>0$. This stripe region is infinite, because of the translational degrees of freedom, but becomes finite once the translations are fixed. We want to show that for the prior from Eq (B) in S1 Text the probability to be in such a domain (and constraining X_1 to a bounded domain) is independent of the

value l_0 in the limit of narrow stripes and unbounded triangles:

$$\begin{aligned}
&\lim_{a\to\infty}\lim_{\epsilon\searrow 0}\left(\frac{\mathbb{P}_X\left[\left\{X_{\tilde{j}}\right\}_{\tilde{j}}\subset B_{ij;\epsilon}\left(l_0^{(1)}\right)\cap\left(A_1\times\mathbb{R}^6\right)\right]}{\mathbb{P}_X\left[\left\{X_{\tilde{j}}\right\}_{\tilde{j}}\subset B_{ij;\epsilon}\left(l_0^{(2)}\right)\cap\left(A_1\times\mathbb{R}^6\right)\right]}\right):=\\
&:=\lim_{a\to\infty}\lim_{\epsilon\searrow 0}\left(\frac{\int_{A_1\times\mathbb{R}^6}\frac{\chi_{\max(l_{12},l_{13},l_{23})<a}\cdot\chi_{l_{ij}\in\left[l_0^{(1)}-\epsilon;l_0^{(1)}+\epsilon\right]}}{l_{12}\cdot l_{13}\cdot l_{23}\cdot\min(l_{12},l_{13},l_{23})}\cdot dX_1dX_2dX_3}{\int_{A_1\times\mathbb{R}^6}\frac{\chi_{\max(l_{12},l_{13},l_{23})<a}\cdot\chi_{l_{ij}\in\left[l_0^{(2)}-\epsilon;l_0^{(2)}+\epsilon\right]}}{l_{12}\cdot l_{13}\cdot l_{23}\cdot\min(l_{12},l_{13},l_{23})}\cdot dX_1dX_2dX_3}\right)=1\\
&\forall ij\in\{12,13,23\},l_0^{(1)},l_0^{(2)}>0,A_1\in\mathbb{A},
\end{aligned}\tag{D}$$

where we used $\mathbb{A}:=\left\{A\subset\mathbb{R}^3\middle|\,0<\int_A dX<\infty\right\}$ to confine node X_1 to a domain of finite volume.

We first show, that

$$d_{flatlenghts}\left[\{X_j\}_j\right]:=\frac{1}{l_{12}\cdot l_{13}\cdot l_{23}}\cdot dX_1dX_2dX_3\tag{E}$$

gives a flat prior on the lengths space (within the boundaries of the triangle inequality), i.e.:

$$\int\limits_A d_{flatlenghts}\left[\{X_j\}_j\right]\propto\int\limits_{\tilde{A}}dl_{12}dl_{13}dl_{23}\;\forall A\in\mathbb{A}_\circ,\tag{F}$$

with $\tilde{A}$ the set of all lengths for which $\{X_j\}_j\in A$ and

$$\mathbb{A}_\circ:=\left\{A_1\times A\subset\mathbb{A}\times\mathbb{R}^6\middle|\,\forall\begin{pmatrix}X_1\\X_2\\X_3\end{pmatrix}\in A_1\times A,R\in SO\left(3\right):\begin{pmatrix}R\cdot X_1\\R\cdot X_2\\R\cdot X_3\end{pmatrix}\in A_1\times A\right\}\tag{G}$$

the set of all rotation symmetric positions with $X_1\in A_1\subset\mathbb{A}$. It is well-known that - for two positions $X_1,X_2\in\mathbb{R}^3$ - we can switch from Cartesian to spherical coordinates via:

$$dX_1dX_2=l_{12}^2\cdot dX_1dl_{12}d\Omega,\tag{H}$$

where $d\Omega=\sin\left(\psi\right)\cdot d\varphi d\psi$ is the differential of the solid angle (for azimuth and inclination angles φ, ψ). On the other hand, we know from the transition from Cartesian to cylinder coordinates (with the X_1–X_2-edge as the axis of the cylinder), that $dX_3=h_3\cdot dh_3dad\theta$, where $h_3\geq 0$ is the distance of X_3 from the cylinder axis, $a\in\mathbb{R}$ is the coordinate along the cylinder axis and $\theta\in[0,2\pi]$ is the rotation angle of X_3 around the cylinder axis (relative to some fixed plane containing that axis). Expressing l_{13}, l_{23} through the cylinder coordinates a, h_3 as well as l_{12} we have:

$$l_{13}^2=a^2+h_3^2\tag{I}$$

$$l_{23}^2=\left(l_{12}-a\right)^2+h_3^2,\tag{J}$$

yielding:

$$\left|\begin{array}{cc}\partial_{h_3}l_{13}&\partial_a l_{13}\\\partial_{h_3}l_{23}&\partial_a l_{23}\end{array}\right|=\left|\begin{array}{cc}\frac{h_3}{l_{13}}&\frac{a}{l_{13}}\\\frac{h_3}{l_{23}}&\frac{a+l_{12}}{l_{23}}\end{array}\right|=\frac{(a+l_{12})\cdot h_3-a\cdot h_3}{l_{13}\cdot l_{23}}=\frac{l_{12}\cdot h_3}{l_{13}\cdot l_{23}}.\tag{K}$$

Combining this with our results for dX_1dX_2 and dX_3 we get:

$$\begin{aligned}dX_1dX_2dX_3&=\left(l_{12}^2\cdot dX_1dl_{12}d\Omega\right)\cdot\left(h_3\cdot dh_3dad\theta\right)=\\&=\left(l_{12}\cdot l_{13}\cdot l_{23}\right)\cdot dl_{12}dl_{13}dl_{23}\cdot\left(dX_1d\Omega d\theta\right),\end{aligned}\tag{L}$$

where the term in the last bracket just gives a constant, thus proving Eq (F) in S1 Text.

Without loss of generality, we show Eq (D) in S1 Text for $ij = 23$, i.e. the marginal over l_{23}. Note, that the additional factor $\min\left(l_{12}, l_{13}, l_{23}\right)^{-1}$ in Eq (B) in S1 Text versus $d_{flatlengths}$ merely comes from the lengths space only being occupied within the boundaries of the triangle inequality (other points are not contained in $\tilde{A}$ in Eq (F) in S1 Text). To see how the extra factor fixes this, we assume an upper cut-off for all lengths $a \gg l_0 > 0$ and divide the marginal integral into different parts depending on which length is shortest:

$$
\begin{aligned}
&\lim_{\epsilon\searrow 0}\left(\frac{1}{2\epsilon}\cdot\int\limits_{A_1\times\mathbb{R}^6}\frac{\chi_{\max(l_{12},l_{13},l_{23})<a}\cdot\chi_{l_{23}\in[l_0-\epsilon;l_0+\epsilon]}}{l_{12}\cdot l_{13}\cdot l_{23}\cdot\min\left(l_{12},l_{13},l_{23}\right)}\cdot dX_1dX_2dX_3\right)\propto\\
&\propto\int\limits_{\left(\mathbb{R}_0^+\right)^2}\frac{\chi_{\max(l_{12},l_{13},l_0)<a}\cdot\chi_{(l_{12}+l_{13}+l_0-2\cdot\max(l_{12},l_{13},l_0))}}{\min\left(l_{12},l_{13},l_0\right)}dl_{12}dl_{13}=\\
&=\int\limits_{l_0}^{a}\int\limits_{l_{12}}^{\min(a,l_0+l_{12})}\frac{1}{l_0}\cdot dl_{13}dl_{12}+\int\limits_{0}^{l_0}\int\limits_{l_0}^{\min(a,l_0+l_{12})}\frac{1}{l_{12}}\cdot dl_{13}dl_{12}+\int\limits_{0}^{l_0}\int\limits_{l_0-l_{12}}^{l_0}\frac{1}{l_{12}}\cdot dl_{13}dl_{12}+\\
&\quad+[\text{same terms with } 2\leftrightarrow 3]=\\
&=2\cdot\left(\int\limits_{l_0}^{a}\min\left(\frac{a-l_{12}}{l_0},1\right)dl_{12}+\int\limits_{0}^{l_0}\min\left(\frac{a-l_0}{l_{12}},1\right)dl_{12}+\int\limits_{0}^{l_0}dl_{12}\right)=2\cdot a+o\left(a\right)
\end{aligned}
\tag{M}
$$

where the terms in the third row correspond to $l_0 \leq l_{12} \leq l_{13}$, $l_{12} \leq l_0 \leq l_{13}$ and $l_{12} \leq l_{13} \leq l_0$ in this order (and then similarly with 2 and 3 swapped). For $a \to \infty$ (i.e. no cut-off of lengths) the l_0-dependent terms $o\left(a\right)$ become negligible, thus proofing our claim from Eq (D) in S1 Text.

Note firstly, this is only an asymptotic argument and for finite cut-offs there are slight variations (see Fig A in S1 Text for a numerical study of the effect of a finite support, $a < \infty$).

Second, the prior on the triangle lengths used here is not unique under the requirement of flat marginals (e.g. a homogeneous weight on the line $l_{12} = l_{13} = l_{23}$ would also satisfy this condition). We also want to highlight that the chosen prior on the lengths is scale invariant, i.e. for a scale parameter $\alpha \in \mathbb{R} \setminus \{0\}$ the ratio $\frac{\pi_X\left[\left\{\alpha\cdot X_j\right\}_j\right]}{\pi_X\left[\left\{X_j\right\}_j\right]} = \alpha^{-4}$ is only a function of α, hence does not depend on the triangle shape.

Markov Chain Monte Carlo samplers for parameter inference

Here we give the details of the Markov Chain Monte Carlo approach taken to sample from the posterior, firstly for the single-state model in Eq (8) and secondly for the multi-state model. A Matlab version (Matlab 2019b) of our code is available. Some of the results presented in the paper were obtained using a different implementation of the MCMC algorithm in Julia (particularly: updating more parameters sequentially rather than jointly), but gives identical posterior distributions.

Samplers of single-state model

We sequentially update the various parameters, $\left\{X_j\right\}_j, \left\{\tau_j\right\}_j, \left\{T^n\right\}_n, \left\{R^n\right\}_n$, using the following samplers:

Random walk samplers Random walk samplers are used for the template positions and perspective parameters, implemented sequentially for each of the parameters X_j, T^n and R^n. We use block updates, blocking together the three coordinates in each of the vectors X_j, and block together the six parameters in T^n and R^n. For the template positions X_j and translations T^n the proposals are uniform balls around the current position. For the rotations R^n the proposal is implemented as follows:

- choose a reference vector $r \in \mathbb{S}^3$ uniformly from the unit-sphere
- draw a sphere location $\tilde{r}$ displaced from r by choosing it uniformly within a spherical cap around r (the radius of the cap is given by the step-size (adjusted during burnin)).
- determine a rotation perturbation $\tilde{R}^n$ as the "geodesic" rotation that maps r onto $\tilde{r}$, i.e. the rotation with invariant axis $r \times \tilde{r}$ around the angle $\arccos(\tilde{r} \cdot r)$
- the proposed rotation is the combined $\tilde{R}^n \cdot R^n$, where R^n is the current rotation.

During burnin the step-sizes of all random walk samplers are adjusted with a target rejection rate of $\rho_\mu := 77.5\%$ (motivated by [1]). The step-size adjustment procedure (given a current step-size of $s > 0$, current adjustment factor of $a > 1$, current deviation $\delta\rho \in \mathbb{R}$ from target acceptance rate ρ_μ and a tolerance of $\rho_\sigma := 7.5\%$):

- compute the rejection rate (of the random walk update of the respective sampler) ρ' since the last step-size evaluation
- compute the new deviation $\delta\rho' := \frac{\rho' - \rho_\mu}{\rho_\sigma}$
- square-root the adjustment factor a, if the deviation $\delta\rho$ has the opposite sign to that of the previous update, i.e. if $\operatorname{sign}(\delta\rho) \neq \operatorname{sign}(\delta\rho')$
- get the new step-size as $s' := s \cdot a^{-\delta\rho'}$
- replace s, $\delta\rho$ for their updated versions s', $\delta\rho'$

We initialise with $a = 2$ and the above five-step procedure was repeated every $\lceil\sqrt{(\#\text{burnin})}\rceil$ Markov chain iterations through burnin, and initial step sizes are chosen heuristically in the range of 10^{-2} and 10 (in nm for lengths, measurement errors).

Gibbs sampler for the translations T^n The translations T^n are also updated using a Gibbs sampler. Here we sample from the target:

$$\mathbb{R}^3 \ni T^n \sim \mathcal{N}\left((\tau')^{-1} \cdot \left(\sum_j \left(\xi_j^n\right)'\right); (\tau')^{-1}\right), \tag{N}$$

where:

$$\mathbb{R}^{3\times 3} \ni \tau' := \left(\sum_j \tau_j\right), \tag{O}$$

$$\mathbb{R}^3 \ni \left(\xi_j^n\right)' := \tau_j \cdot \left(\tilde{X}_j^n - \left(R^n \cdot (X_j - X_1) + X_1\right)\right). \tag{P}$$

Table A. Number of Markov chain iterations in the examples presented in the main part.

Single-state simulated examples (see Table 1 for results):

Example	1.1	1.2	1.3	1.4	1.5	1.6
number of MCMC iterations	100000	100000	500000	500000	500000	500000

Two-state simulated examples (see Table 2 for results):

Example	2.1	2.2	2.3	2.4	2.5	2.6
number of MCMC iterations	50000	50000	50000	50000	50000	50000

Single-state experimental examples (see Table 3 for results):

Example	3.1	3.2	3.3	3.4	3.5
number of MCMC iterations	200000	100000	100000	100000	500000

Two-state experimental examples (see Table 4 for results):

Example	4.1	4.2	4.3
number of MCMC iterations	1000000	200000	1000000

In all examples the first 40% of the iterations is burn-in, and the remaining 60% the posterior samples.

Gibbs sampler for the measurement errors τ_j A Gibbs sampler is used for the precisions $\tau_j = \begin{pmatrix} \sigma_{j;xy}^{-2} & 0 & 0 \\ 0 & \sigma_{j;xy}^{-2} & 0 \\ 0 & 0 & \sigma_{j;z}^{-2} \end{pmatrix}$ of the measurement errors of the three fluorescent spots. Note that the two components xy, z as well as the three fluorophores j are independent from each other, so sequential and joint updates coincide. We have a Γ-distribution for each of the conditional distributions:

$$\mathbb{R}_0^+ \ni \sigma_{j;d}^{-2} \sim \Gamma\left(\frac{-3}{2} + 1 + \frac{N}{2} \cdot (\#d)\,;\left(\sum_{n,d'} \frac{1}{2} \cdot \left(X_{j;d'}^n - \tilde{X}_{j;d'}^n\right)^2\right)\right), \tag{Q}$$

where $d \in \{xy, z\}$ and number of dimensions $(\#d) = 2$, 1, respectively.

Initialisation and convergence monitoring Unless otherwise stated, the variables of all chains are initialised randomly as follows (independent for each j, n):

$$X_j : |X_j - X_i| \sim \mathcal{U}\left([0, 300\text{nm}]\right) \tag{R}$$
$$\sigma_{j;d} : \sigma_{j;d} \sim \mathcal{U}\left([0, 200\text{nm}]\right) \tag{S}$$
$$T^n : T^n \sim \mathcal{U}\left([-20000\text{nm}, +20000\text{nm}]^3\right) \tag{T}$$
$$R^n : R^n \sim \pi_R\left[R^n\right]. \tag{U}$$

These are over-dispersed compared to the anticipated width of the posterior distribution (confirmed after the run). The total number of iterations per Markov chain for each example is reported in Table A in S1 Text; sub-sampling (equally spaced) was used to give a final sample size of no more than 10000 samples (3000 in Example 4.1).

A multi-chain convergence diagnostic was used (5 independent chains), assessing convergence by computing the Gelman-Rubin statistic $\hat{R}$ (uncorrected) separately on parameters of interest, specifically $|X_j - X_i|$, $\sigma_{j;d}$ for $i, j \in \{1, 2, 3\}$, $d \in \{xy, z\}$,

(see [2, Ch 11.6]). We used a threshold of 1.1; if converged, the five chains were then pooled to reconstruct the posterior.

The computation time on an ordinary desktop computer is about 1 day for datasets with $N = 200$ samples (our Matlab implementation may be further improved for speed).

Samplers of multi-state model

All parameters already present in the single-state model from subsection Polygon parametrisation and inference can be inferred using the same updates, if confining the measured data points to the subset in the currently updated state $\zeta^n = \zeta$. The new variables $\{\zeta^n\}_n$ and $\{p^{(\zeta)}\}_\zeta$ are updated sequentially with random walk and Gibbs samplers, respectively.

Random walk sampler for state-affiliations $\{\zeta^n\}_n$ The hidden state-affiliations $\{\zeta^n\}_n$ are sampled with a random walk proposal, equiprobable on all states except the current one (assigned zero probability). To achieve a higher acceptance rate for a new state-affiliation proposal, the translation T^n for measurement n are altered such that the centre of mass of the triangle (with equal weights for all nodes j) of the proposed true positions $\{X_j^n\}_j$ remain unchanged.

Gibbs sampler for state proportions $\mathcal{P} = \{p^{(\zeta)}\}_\zeta$ The state proportions $\mathcal{P}$ are sampled via a Gibbs sampler from a Dirichlet distribution:

$$\left\{p^{(\zeta)}\right\}_\zeta \sim \mathrm{Dir}\left((1 + \#1), \ldots, (1 + \#Z)\right), \tag{V}$$

where $\#\zeta' := |\{\, n \in \{1, \ldots, N\} |\, \zeta^n = \zeta'\}|$ are the number of measurements in the respective states. The Dirichlet probability density for the state proportions $\{p^{(\zeta)}\}_\zeta$ is

$$\mathrm{Dir}\left(\alpha_1, \ldots, \alpha_Z\right) \propto \left(\prod_{\zeta \in \{1,\ldots,Z\}} \left(p^{(\zeta)}\right)^{(\alpha_\zeta - 1)}\right), \tag{W}$$

with the state proportions all constrained within $[0; 1]$ and summing to one.

Initialisation and convergence monitoring The parameters were randomly initialised (independent for each j, n; for Eq (AF) in S1 Text priors):

$$X_j^{(1)} : \left|X_j^{(1)} - X_i^{(1)}\right| \sim \mathcal{U}\left(\left[l_{ij;0}^{(1)} - 12\mathrm{nm}, l_{ij;0}^{(1)} + 12\mathrm{nm}\right]\right) \tag{X}$$

$$X_j^{(2)} : \left|X_j^{(2)} - X_i^{(2)}\right| \sim \mathcal{U}\left([0, 300\mathrm{nm}]\right) \tag{Y}$$

$$\sigma_{j;d}^{(1)} : \left(\sigma_{j;d}^{(1)}\right)^{-2} \sim \Gamma\left(5; \tau_{j;d;0}^{(\zeta)}\right) \tag{Z}$$

$$\sigma_{j;d}^{(2)} : \sigma_{j;d}^{(2)} \sim \mathcal{U}\left([0, 200\mathrm{nm}]\right) \tag{AA}$$

$$T^n : T^n \sim \mathcal{U}\left([-20000\mathrm{nm}, +20000\mathrm{nm}]^3\right) \tag{AB}$$

$$R^n : R^n \sim \pi_R\left[R^n\right] \tag{AC}$$

$$\left\{p^{(\zeta)}\right\}_\zeta : \left\{p^{(\zeta)}\right\}_\zeta \sim \mathrm{Dir}\left(\begin{array}{cccc} 1 & 1 & \ldots & 1 \end{array}\right) \tag{AD}$$

$$\{\zeta^n\}_n : \zeta^n \sim Cat\left(\left\{p^{(\zeta)}\right\}_\zeta\right), \tag{AE}$$

where $Cat\left(\left\{p^{(\zeta)}\right\}_{\zeta}\right)$ is the multinoulli distribution, state ζ being drawn with probability $p^{(\zeta)}$. The values $l_{ij;0}^{(1)}$ and $\tau_{j;d;0}^{(1)}$ are identical to the priors in Eq (AF) in S1 Text and given for each example individually in the main text. These are chosen to be overdispersed compared to the expected posteriors (and confirmed a-posteriori), except for the priors for the lengths and measurement errors of the first state.

Convergence is monitored as described above for the single-state model, where additionally each of the $p^{(\zeta)}$ has to stay below the Gelman-Rubin threshold of 1.1. The number of Markov chain iterations for the examples is given in Table A in S1 Text.

A multi-state informing prior: simulations only

In the simulated multi-state examples, Table 2, we utilised more confining priors on one or more of the states to improve convergence. Specifically, the triangle side lengths are independently constrained to a cuboid-shaped domain in the lengths-space and the precisions are Gamma distributed. For instance, to impose this prior on state $\zeta = 1$, the following factors are included in the prior Eq (7),

$$\left(\prod_{i,j} \chi_{\left|X_j^{(1)} - X_i^{(1)}\right| \in \left[l_{ij;0}^{(1)} - L; l_{ij;0}^{(1)} + L\right]}\right) \cdot \left(\prod_{j,d} \Gamma\left(\tau_{j;d}^{(1)};\, 5, \frac{1}{5 \cdot \left(\sigma_{j;d;0}^{(1)}\right)^2}\right)\right), \qquad \text{(AF)}$$

where the parameters $l_{ij;0}^{(1)} \geq 0$ and $\sigma_{j;d;0}^{(1)} > 0$ are specified by prior knowledge (analysis of previous data or structural data), and the size $2L$ is taken as 24nm. The second and third parameter of the Γ distribution are the shape and scale parameters. The values for L and the shape parameter are a particular choice for our examples, to be informative enough to help mixing during burnin (see supplementary section Markov Chain Monte Carlo samplers for parameter inference in S1 Text) with negligible effect on the posterior distribution.

Number of parameters in the model

The total number of inferred parameters can be calculated as follows:

$$(Z-1) + ((3 \cdot 2 + 2 \cdot 1) - 6) \cdot Z + (5 + \chi_{Z>1}) \cdot N \qquad \text{for } J = 2 \qquad \text{(AG)}$$

$$(Z-1) + ((3+2) \cdot J - 6) \cdot Z + (6 + \chi_{Z>1}) \cdot N \qquad \text{for } J > 2, \qquad \text{(AH)}$$

where we calculated $\chi_{Z>1}$ degrees of freedom for each state affiliation ζ^n (indicator function χ). The first term comes from the state proportions, the second from the model parameters $\left\{\theta_j^{(\zeta)}\right\}_{j,\zeta}$ and the third from the perspectives and state affiliations $\{\theta^n\}_n$, $\{\zeta^n\}_n$ (minus symmetries). The different formula for $J = 2$ fluorophores occurs due to the higher symmetry (in the above model the rotations still have three parameters, but the posterior is invariant with respect to the rotation around the template axis) and the indistinguishability of the measurement errors discussed in subsection Testing the single-state model on simulated data. In case of shared states across multiple datasets, the duplicate parameters have to be subtracted again. The measurements give $3 \cdot J \cdot N$ constraints (3D vectors for J nodes per measurement, N measurements).

Simulated data

To test the algorithm we simulated data based on the model described in section Materials and methods. For the simulations the template positions $\left\{X_j^{(\zeta)}\right\}_{j,\zeta}$,

the measurement errors $\left\{\sigma_{j;d}^{(\zeta)}\right\}_{j,\zeta}$ and the state proportions $\left\{p^{(\zeta)}\right\}_{\zeta}$) are fixed as Tables 1, 2. The state affiliations $\{\zeta^n\}_n$ are chosen to exactly fit the state proportions, i.e. $p^{(\zeta)} = \frac{\#\{n|\zeta^n=\zeta\}}{N_{\mathrm{mix}}}$. The translations $\{T^n\}_n$ are independently sampled from Gaussians:

$$T^n \sim \mathcal{N}\left(0; \begin{pmatrix} 20\mathrm{nm} & 0 & 0 \\ 0 & 1000\mathrm{nm} & 0 \\ 0 & 0 & 1000\mathrm{nm} \end{pmatrix}^2\right), \tag{AI}$$

and the rotations are sampled from converged chains of a Markov Chain Monte Carlo algorithm using the same random walk update as described in subsection Samplers of single-state model in S1 Text. The random variables modelling the measurement error, $\left\{\gamma_j^n\right\}_{j,n}$, are sampled independently from their respective Gaussians:

$$\gamma_j^n \sim \mathcal{N}\left(0; \begin{pmatrix} \sigma_{j;xy}^{(\zeta^n)} & 0 & 0 \\ 0 & \sigma_{j;xy}^{(\zeta^n)} & 0 \\ 0 & 0 & \sigma_{j;z}^{(\zeta^n)} \end{pmatrix}^2\right). \tag{AJ}$$

Four-fluorophore simulated example

We present a simulated example for $J = 4$ fluorophores and $N = 400$ measurements. Original and inferred values for lengths and measurement errors are given in Table B in S1 Text, demonstrating good agreement.

Having more than three markers requires an extension to the prior on the template positions given in supplementary section Flat prior on marginals of lengths in S1 Text. We suggest to generalise that procedure (without proof; see Fig B in S1 Text for a numerical evaluation) by the recursive Algorithm A in S1 Text. As before, this prior is meant to be asymptotically (for unbounded domains) flat on the length-marginals, but is not uniquely defined by this property.

Table B. Single-state simulated example for four fluorophores ($N = 400$).

(lengths and errors in nm)	$\lvert X_2 - X_1\rvert$	$\lvert X_3 - X_1\rvert$	$\lvert X_4 - X_1\rvert$	$\lvert X_3 - X_2\rvert$	$\lvert X_4 - X_2\rvert$	$\lvert X_4 - X_3\rvert$
true value	45	45	35	85	30	70
pyramid, uninformative prior	46.7 ± 1.0	44.3 ± 1.3	35.4 ± 1.1	84.7 ± 1.2	30.1 ± 1.2	69.2 ± 1.3

(lengths and errors in nm)	$\sigma_{1;xy}$	$\sigma_{1;z}$	$\sigma_{2;xy}$	$\sigma_{2;z}$	$\sigma_{3;xy}$	$\sigma_{3;z}$	$\sigma_{4;xy}$	$\sigma_{4;z}$
true value	10	20	10	20	15	30	10	30
pyramid, uninformative prior	9.9 ± 0.6	18.3 ± 1.3	10.0 ± 0.8	21.1 ± 1.5	14.7 ± 0.9	27.8 ± 1.8	11.1 ± 0.7	32.2 ± 1.4

Rows are the simulated input values and the posterior means ± standard deviations of the four-fluorophore version of our algorithm, respectively.

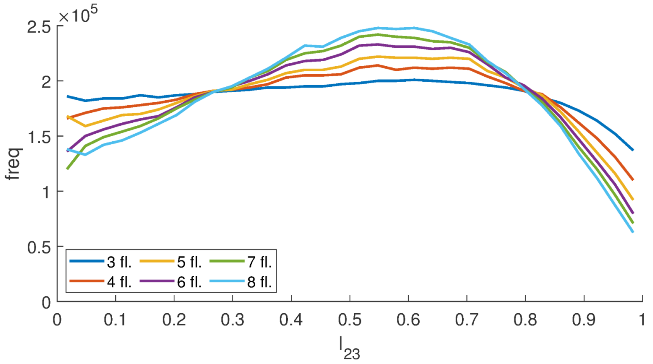

Fig B. Numerical simulation of the histograms for the suggested priors on the template positions $\{X_j\}_j$. Marginal densities are shown on the space of the polygon lengths (symmetric for all lengths). A cut-off for each of the lengths was used, forcing them to be within $[0, 1]$ - note that this boundary effect becomes more pronounced for higher dimensions. The symmetry of the prior with respect to re-labelling fluorophores j was used when sampling.

Algorithm A: Recursive algorithm for prior on template positions, $\pi_X\left[\{X_j\}_j\right]$

Function Tmpltprior$\left(\{X_j\}_{j\in\mathbb{J}}\right)$

$\pi_X\left[\{X_j\}_{j\in\mathbb{J}}\right] = 1$ `// initialise`

$J' = \#\mathbb{J}$ `// number of fluorophores here`

if $J' \geq 2$ **then** `// otherwise recursion stops`

$(j_1, j_2) =$ **Getendpointsofminimallength** $\left(\{X_j\}_{j\in\mathbb{J}}\right)$

$$\pi_X\left[\{X_j\}_{j\in\mathbb{J}}\right] = \frac{\pi_X\left[\{X_j\}_{j\in\mathbb{J}}\right]}{\left|X_{j_1}-X_{j_2}\right|^2} \cdot \sqrt{\mathbf{Tmpltprior}\left(\{X_j\}_{j\in\mathbb{J}\setminus\{j_1\}}\right) \cdot \mathbf{Tmpltprior}\left(\{X_j\}_{j\in\mathbb{J}\setminus\{j_2\}}\right)}$$

return $\pi_X\left[\{X_j\}_{j\in\mathbb{J}}\right]$

Implementation of pair-wise correction methods

We compared our method to existing length inference techniques between two fluorophores, namely by Churchman et al from [3] and BEDCA, [4]. Here we describe our implementation of these methods:

Churchman et al [Isotropic measurement errors only]

Maximum likelihood estimate (MLE): we used the in-built Matlab functions `simulannealbnd` then `fminsearch` on the 3D likelihood as specified in Eq (6) in [3]. For error estimates we calculated the Hessian of the likelihood and used its negative for the inverse covariance matrix of the Gaussian approximation.

Mean and standard deviation of the full posterior: we used the same likelihood function and assumed flat priors on the lengths $|X_j - X_i|$ and measurement errors σ_{ij}. The marginal distributions were computed using numerical integration on a rectangular grid.

BEDCA This is the algorithm used in [4] and [5]; however we used different priors analogous to the ones used in the triangle method (see Eq (A) in S1 Text). Specifically, measurement errors have flat priors on the positive real axis ($d\sigma_{j;d}$) and lengths have almost flat priors on the positive real axis ($\propto \chi_{l_{ij}\geq 0} \cdot \mathcal{N}\left(60; 10^{10}\right) \cdot dl_{ij}$ for length $l_{ij} = |X_j - X_i|$). Note, the latter gives (approximately) flat priors on the lengths (i.e. the length marginals for triangles) but if the three lengths are used to form a triangle under an independence assumption (rejecting those combinations that violate the triangle inequality), the prior would lack the $\min\left(\{l_{12}, l_{13}, l_{23}\}\right)$ term from Eq (B) in S1 Text (see subsection Flat prior on marginals of lengths in S1 Text for details).

Small lengths are increasingly difficult to infer

Here we give an argument, why for a given measurement error, shorter true lengths exhibit a much larger inference error than longer lengths. This phenomenon has been reported in [6] for the length correction based on [3] before.

There are two stochastically independent processes contributing to the distribution of the vectors between two fluorophores, $\tilde{X}_j^n - \tilde{X}_i^n$. First, the isotropic distribution of the displacement vectors between the true positions in space (due to the rotations R^n) and second, the measurement error (a Gaussian measurement error assumption is not required). Computing the second moment of the distance we obtain:

$$\begin{aligned}\mathbb{E}_n\left[\left|\tilde{X}_j^n - \tilde{X}_i^n\right|^2\right] &= \operatorname{trace}\left(\operatorname{Cov}_n\left(\tilde{X}_j^n - \tilde{X}_i^n\right)\right) = \\ &= \operatorname{trace}\left(\Sigma_{ij}\right) + \operatorname{trace}\begin{pmatrix} \frac{|X_j - X_i|^2}{3} & 0 & 0 \\ 0 & \frac{|X_j - X_i|^2}{3} & 0 \\ 0 & 0 & \frac{|X_j - X_i|^2}{3} \end{pmatrix} = \\ &= \operatorname{trace}\left(\Sigma_{ij}\right) + \left|X_j - X_i\right|^2, \end{aligned} \tag{AK}$$

where the expectation and covariance are taken with respect to the fluorophore specific random variables (parametrised with n), i.e. measurement errors γ_j^n, rotations R^n and translations T^n; the template positions X_j and measurement errors $\sigma_{j;d}$ are fixed. In the second line Σ_{ij} is the covariance matrix of the measurement error of the displacement (in the setting described in section Materials and methods this would be $\Sigma_{ij} = \begin{pmatrix} \sigma_{i;xy}^2 & 0 & 0 \\ 0 & \sigma_{i;xy}^2 & 0 \\ 0 & 0 & \sigma_{i;z}^2 \end{pmatrix} + \begin{pmatrix} \sigma_{j;xy}^2 & 0 & 0 \\ 0 & \sigma_{j;xy}^2 & 0 \\ 0 & 0 & \sigma_{j;z}^2 \end{pmatrix}$) and the matrix in the last term is the covariance of the Lebesgue measure on a sphere of radius $|X_j - X_i|$. Expression (AK) in S1 Text is the 3D analogue to Eq (4) in [7]. To quantify how strongly $|X_j - X_i|$ depends on the measurement error, we take the derivative of (AK) in S1 Text:

$$\left|\partial_{\sqrt{\operatorname{trace}(\Sigma_{ij})}} \left|X_j - X_i\right|\right| = \frac{\sqrt{\operatorname{trace}\left(\Sigma_{ij}\right)}}{\left|X_j - X_i\right|} \gg 1, \tag{AL}$$

when the true length is much smaller than the measurement error. Thus, small changes in the inferred measurement error lead to very large changes in the inferred true length, with increasing effect for small lengths. See Fig C in S1 Text for a graphical depiction.

Computation of p values

For comparisons of posteriors, we define the overlap probability

$$p = \min\left(\mathbb{P}\left[l_{ij;\text{method 1}} < l_{ij;\text{method 2}}\right], \mathbb{P}\left[l_{ij;\text{method 1}} > l_{ij;\text{method 2}}\right]\right), \tag{AM}$$

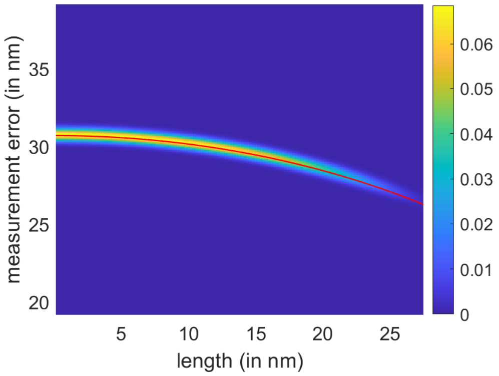

Fig C. Posterior distribution for measurement error and length for 3D simulated data. Posterior based on $N = 2000$ samples simulated with an input (true) length of 15nm and input (true) measurement error of $\sqrt{15^2 + 25^2}$nm $\approx$ 29nm. Plotted is the likelihood function, Eq (6) from [3]. Probability density is colour coded as key. The red line is Eq (AK) in S1 Text with the second moment (left hand side) estimated from the data.

for length l_{ij} inferred by methods 1, 2. If both methods had identical posteriors, we have $p = 50\%$.

Experimental methods: prometaphase-metaphase dataset

For the prometaphase-metaphase data of Example 4.1 we used immortalised (hTERT) diploid human retinal pigment epithelial (RPE1) cells in which the Ndc80 C-terminus was labelled at the endogenous locus with eGFP (MC191). These RPE1 Ndc80-EGFP (MC191) were cultured and imaged for CenpC–Ndc80C–Ndc80N as [5]. In brief, the MC191 cells were cultured in a humidified incubator at 37 degrees, 5% CO2, in DMEM-F12 (Gibco) media supplemented with 10% Foetal Bovine Serum (FBS), 2mM L-Glutamine, 100U/ml penicillin and 100μg/ml streptomycin. MC191 cells were fixed in 10mM EGTA, 1mM MgCl2, 20mM PIPES pH 6.8, 0.2% Triton X-100, and 4% formaldehyde for 10min. Next, the cells were washed 3 times for 5min with PBS and incubated with 3% Bovine Serum Albumine (BSA) in PBS for 30min. After that, the MC191 cells were incubated with Guinea Pig anti-CenpC (1 : 2000; MBL, Cat#PD030) and Mouse anti-Ndc80(N) (9G3, 1 : 1000; Abcam, Cat#ab3613) primary antibodies for 1hr. MC191 cells were washed 3 times for 5min with PBS and incubated with Goat anti-guinea pig Alexa Fluor 568 (1 : 500) and Goat anti-mouse Alexa Fluor 647 (1 : 500) secondary antibodies. MC191 cells were washed 3 times for 5min with PBS and mounted in VectaShield (Vector, Cat#H-1000). Image stacks were acquired on spinning disk microscope (VOX UltraView; PerkinElmer, UK) equipped with a 100X / 1.4 NA oil-immersion objective and a Hamamatsu ORCA-R2 camera, controlled by Volocity 6.0 (PerkinElmer) running on a Windows 7 64-bit (Microsoft, Redmond, WA) PC (IBM, New Castle, NY). The images were acquired using 488nm, 561nm, 647nm, and 405nm wavelength lasers. The sampling was set to z-spacing of 0.2μm and 61 z-slices. Spinning disk images were exported in OME.TIFF format (The Open Microscopy Environment, UK) from Volocity 6.0 and deconvolved using Huygens 4.1. The images were then analysed in KiDv1.0.1 for spot detection, approximate chromatic shift correction, kinetochore sister-sister pairing, and spot quality control as detailed in [4]. Chromatic shift correction samples were prepared and used as detailed in [4].

For assignment of cells as early prometaphase, late prometaphase or metaphase, the

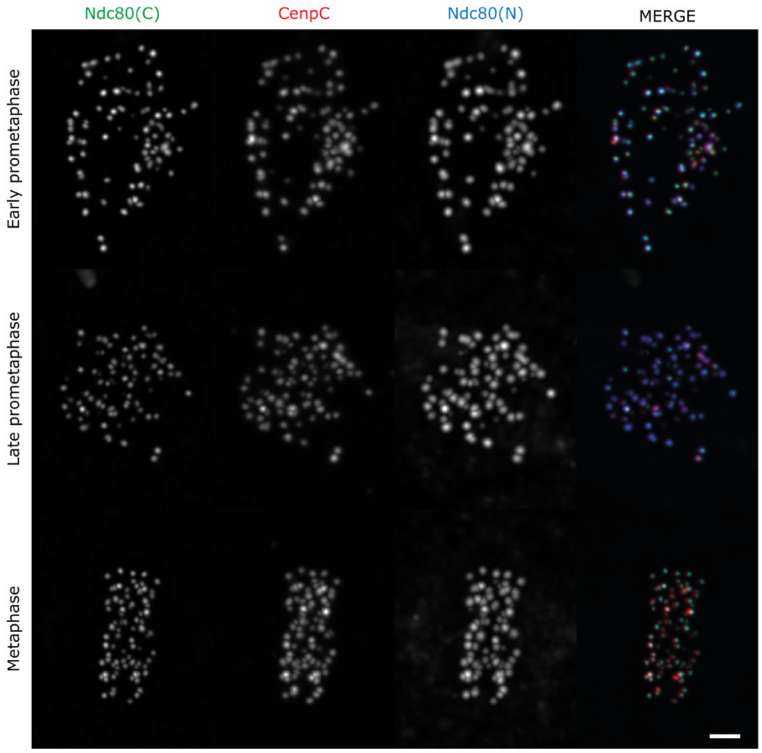

Fig D. Example cells identified as in early prometaphase (top), late prometaphase (middle) or metaphase (bottom), respectively. Depicted are the three channels of three example cells of the dataset of Example 4.1. Scale bar is 2μm.

image stacks were opened in Fiji, and mitotic phase was manually assigned based on kinetochore alignment in a metaphase plate, i.e. cells most of the kinetochores not aligned in a plate were assigned as early prometaphase, cells with few kinetochores not aligned in a plate were assigned as late prometaphase, and cells with all kinetochore aligned in a plate were as assigned as metaphase, see Fig D in S1 Text.

Triangle length means never violate the triangle inequality

For any (proper) probability distribution on the triangle side lengths, $l_{ij} \geq 0$, $i, j \in \{1, 2, 3\}$, the length means necessarily satisfy the triangle inequality (provided they are finite). To prove this, we use Jensen's inequality ([8, Thm 3.1.3]) in combination with convexity of the max function to write:

$$\left(\sum_{j>i} \mathbb{E}\left[l_{ij}\right]\right) - 2\max_{i,j}\left(\mathbb{E}\left[l_{ij}\right]\right) \geq \left(\sum_{j>i} \mathbb{E}\left[l_{ij}\right]\right) - 2\cdot\mathbb{E}\left[\max_{i,j}\left(l_{ij}\right)\right] =$$
$$= \mathbb{E}\left[\left(\sum_{j>i} l_{ij}\right) - 2\max_{i,j}\left(l_{ij}\right)\right] \geq 0 \qquad \text{(AN)}$$

Model comparison: Two-state vs single-state model

In subsection Experimental dataset analysis with multi-state model algorithm we analysed metaphase and prometaphase kinetochores in DMSO with the two-state model Example 4.1). Here we show, how the model comparison with the single-state model

was carried out for the metaphase and late prometaphase subset (for early prometaphase the same procedure was followed upon swapping the state labels $1 \leftrightarrow 2$): Apart from the state proportions $\left\{p^{(\zeta)}\right\}_{\zeta}$, the single-state model is a nested sub-model of the two-state model (i.e. it is absolutely continuous) with all state affiliations $\{\zeta^n\}_n$ equal to the same state ζ. We can therefore compute the Bayes factor (see e.g. [9]) of these two models based on our MCMC samples of the two-state model alone. Let ϕ_{ts} and ϕ_{ss} denote the model parameters of the two- or single-state models, respectively, except for the state affiliations $\{\zeta^n\}_n$. Their corresponding parameter spaces are denoted Φ_{ts}, Φ_{ss}). The Bayes factor is then given by:

$$\begin{aligned}
&\frac{\mathbb{P}\left[\left\{\tilde{X}_j^n\right\}_{j,n}, \phi_{ts} \in \Phi_{ts}, \{\zeta^n\}_n \in \{1,2\}^N \middle| \text{two-state model}\right]}{\mathbb{P}\left[\left\{\tilde{X}_j^n\right\}_{j,n}, \phi_{ss} \in \Phi_{ss} \middle| \text{single-state model}\right]} = \\
&= \frac{\mathbb{P}\left[\left\{\tilde{X}_j^n\right\}_{j,n}, \phi_{ts} \in \Phi_{ts}, \{\zeta^n\}_n \in \{1,2\}^N \middle| \text{two-state model}\right]}{\mathbb{P}\left[\left\{\tilde{X}_j^n\right\}_{j,n}, \phi_{ss} \in \Phi_{ss} \middle| \text{single-state model}\right] \cdot (N+1) \int_0^1 \left(1-p^{(2)}\right)^N dp^{(2)}} = \\
&= \frac{1}{N+1} \cdot \frac{\mathbb{P}\left[\left\{\tilde{X}_j^n\right\}_{j,n}, \phi_{ts} \in \Phi_{ts}, \{\zeta^n\}_n \in \{1,2\}^N \middle| \text{two-state model}\right]}{\mathbb{P}\left[\left\{\tilde{X}_j^n\right\}_{j,n}, \phi_{ts} \in \Phi_{ts}, \{\zeta^n\}_n \in \{1\}^N \middle| \text{two-state model}\right]}, \qquad \text{(AO)}
\end{aligned}$$

where the last identity comes from the fact that when we confine the multi-state model to the case where all measurements are in one state, $\{\zeta^n\}_n \in \{1\}^N$, the likelihood times the prior is the same as its single-state counterpart, apart from the extra factor from the Dirichlet distribution, $\chi_{p^{(2)} \in [0,1]} \cdot \left(1-p^{(2)}\right)^N \cdot dp^{(2)}$ (note we have the same priors on lengths and measurement errors in both models). The right-hand ratio in Eq (AO) in S1 Text can be estimated from our MCMC of the two-state model by counting how often the chain is in the pure state 1. We abbreviate this ratio by ω. Our discussion so far has assumed a flat prior on the state proportion for the two-state model, $\chi_{p^{(2)} \in [0,1]} \cdot dp^{(2)}$. For analysis of a heterogeneous population with a minor sub-population, a flat prior on the smaller interval $[0, \alpha]$ for $\alpha \in [0,1]$ appears more realistic. In this case the Bayes factor in Eq (AO) in S1 Text changes to: $\frac{1-(1-\alpha)^{N+1}}{\alpha \cdot (N+1)} \cdot \omega_\alpha$.

From the Bayes factor we immediately get the probability of the two-state model (assuming a-priori equiprobable models):

$$\begin{aligned}
p &= \frac{\mathbb{P}\left[\text{two-state model}, \phi_{ts} \in \Phi_{ts}, \{\zeta^n\}_n \in \{1,2\}^N \middle| \left\{\tilde{X}_j^n\right\}_{j,n}, \alpha\right]}{\mathbb{P}\left[\text{single-state model}, \phi_{ss} \in \Phi_{ss} \middle| \left\{\tilde{X}_j^n\right\}_{j,n}\right] + \mathbb{P}\left[\text{two-state model}, \phi_{ts} \in \Phi_{ts}, \{\zeta^n\}_n \in \{1,2\}^N \middle| \left\{\tilde{X}_j^n\right\}_{j,n}, \alpha\right]} = \\
&= \frac{\frac{1-(1-\alpha)^{N+1}}{\alpha \cdot (N+1)} \cdot \omega_\alpha}{1 + \frac{1-(1-\alpha)^{N+1}}{\alpha \cdot (N+1)} \cdot \omega_\alpha}. \qquad \text{(AP)}
\end{aligned}$$

This is plotted as a function of α in Fig E in S1 Text (for Example 4.1). The metaphase value $p_{\text{two-states;meta}}$ in the main text is computed for $\alpha = 20\%$, while for the prometaphase values $p_{\text{two-states;late prometa}}$, $p_{\text{two-states;early prometa}}$ we used $\alpha = 100\%$ (i.e. for metaphase we expected the attached state to be dominant, while for prometaphase we were indifferent).

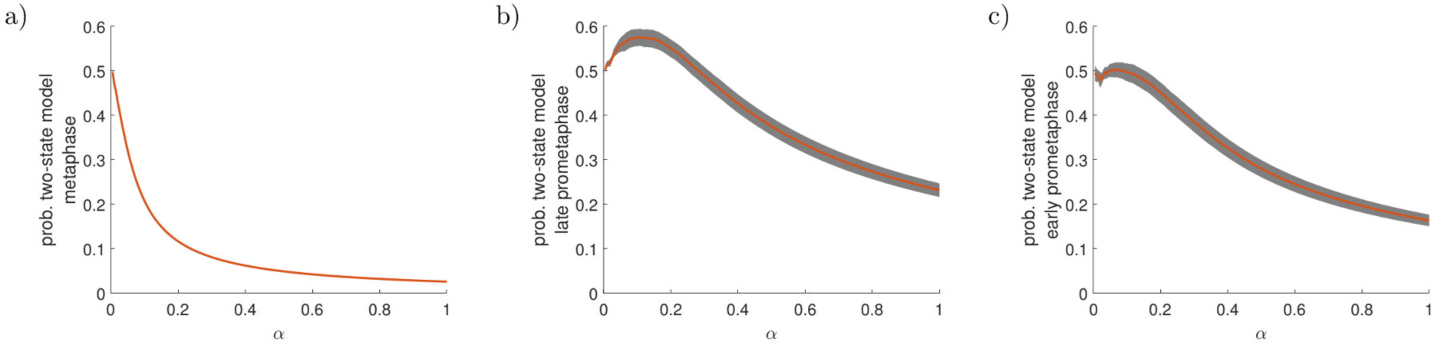

Fig E. Model comparison between two-state and single-state model for Example 4.1. Dependence of the probability of the two-state model on the prior parameter α, as given in Eq (AP) in S1 Text. Panel a) shows the analysis for the metaphase subset, panel b) for the late prometaphase subset, panel c) for the early prometaphase subset. The orange line denotes the mean over the five independent runs, while the shaded area is the $\pm 1\sigma$ range for each α. "Substantial" evidence for a model is typically acknowledged for probabilities $\geq 76\%$ (i.e. Bayes factors ≥ 3.2; [9]).

Experimental dataset analysis with multi-state model algorithm, second experiment

Here we present a second, experimentally equivalent but smaller dataset to the one analysed in subsection Experimental dataset analysis with multi-state model algorithm, Example 4.1. Being split by eye into metaphase, late prometaphase and early prometaphase, it contains $N_{\text{meta}} = 152$, $N_{\text{late prometa}} = 108$ and $N_{\text{early prometa}} = 78$ kinetochores in $C_{\text{meta}} = 5$, $C_{\text{late prometa}} = 3$ and $C_{\text{early prometa}} = 3$ cells, respectively. Results are shown in Example C.4 and Fig F in S1 Text being qualitatively similar to Example 4.1 presented in the main text.

There is evidence for two states being present in late prometaphase with $p_{\text{two-states;late prometa}} = (100.0 \pm 0.0)\,\%$, while for metaphase and early prometaphase the single-state model is preferred, $p_{\text{two-states;meta}} = (17.2 \pm 0.2)\,\%$, $p_{\text{two-states;early prometa}} = (10.7 \pm 0.3)\,\%$. Examining the relation of the inferred mean state affiliations $\left\{\bar{\zeta}^n\right\}_n$ with the tension parameters, exhibits a clear trend of kinetochores that have a high probability of being in the natural unattached conformation towards smaller sister-sister distances, l_{ss} (correlation -0.319% with $p = 2.0 \times 10^{-9}$), and larger swivels, ϑ (correlation $+0.300\%$ with $p = 1.9 \times 10^{-8}$). See Table K in S1 Text for results for each mitotic phase.

We did not pool this with the data presented in the main text, due to the significantly different Ndc80C–Ndc80N length (see Fig G in S1 Textf) and several measurement errors being smaller.

Cell-based subset analysis inference of mitotic phase variables

The dataset of Example 4.1, was analysed with 31 cell-based subsets, each cell having its own independent state proportion; the state conformations and measurement errors are shared across all cells. To infer mitotic phase state proportions, all kinetochores from cells in that phase are sampled using the MCMC runs over respective cells. Namely, the state proportion of a mitotic phase is given by $\left\{\tilde{p}^{(\zeta)}\right\}_\zeta \sim \text{Dir}\left((1 + \#1), \ldots, (1 + \#Z)\right)$, where $\#1, \#2..$ are the number of kinetochores in the respective state and respective cell phase, sampled from the MCMC runs (see subsection Samplers of multi-state model in S1 Text). Although flat priors are used for the state proportion in each cell, the prior for this sampled mitotic phase state proportion is non-uniform, see Fig H in S1 Text. This prior is a result of assuming cells are independent. The prior

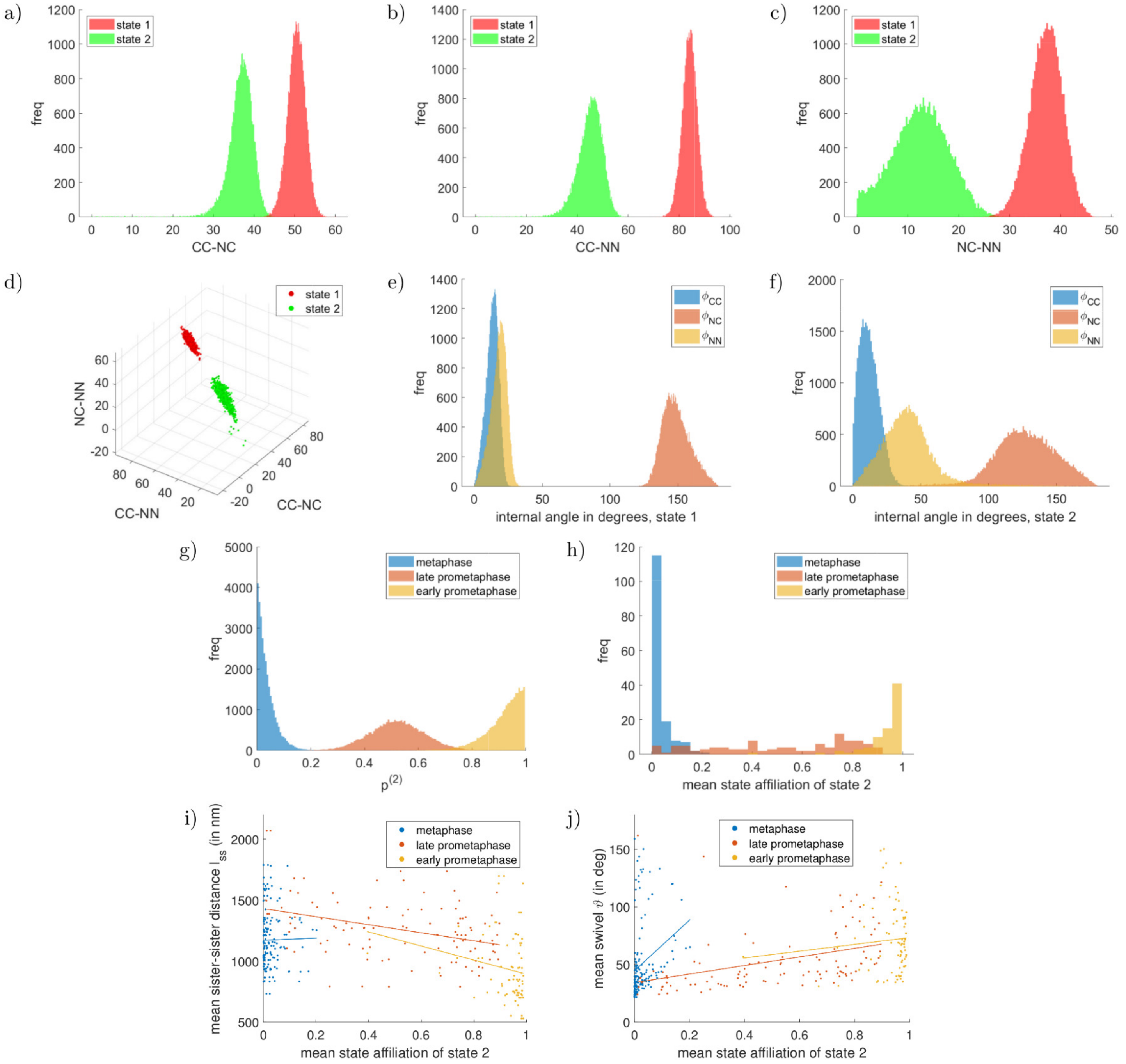

Fig F. Two-state example for experimental prometaphase-metaphase dataset, second dataset, Example C.4. Marginal lengths (in nm; pooled over metaphase and prometaphase) in panels a)–d), the majority state in metaphase is depicted in red, the minority state in green. See supplement Uninformative priors in S1 Text for the joint prior on the lengths. Marginals of the internal angles of the two states are shown in panels e), f). The marginal state proportions of the metaphase-minority state is depicted for each mitotic phase in panel g). The prior on each state proportion is flat in $[0, 1]$. The mean state affiliations $\{\bar{\zeta}^n\}_n$ for each mitotic phase is shown in panel h), exhibiting clearly separated preferences for kinetochores in metaphase and early prometaphase, while late prometaphase contains some kinetochores that are likely attached, some likely unattached and some undecided. The shown mean state affiliations are with respect to the natural unattached state, i.e. kinetochores with a value close to one would be most likely in jthe natural unattached state. The bottom row compares the inferred mean state affiliations of each kinetochore with the tension parameters: Panel i) shows the mean sister-sister distance l_{ss}, panel j) the mean swivel ϑ, where the mitotic phase is colour-coded and the straight lines show the best fit linear model for each phase. For significance tests, see Table K in S1 Text.

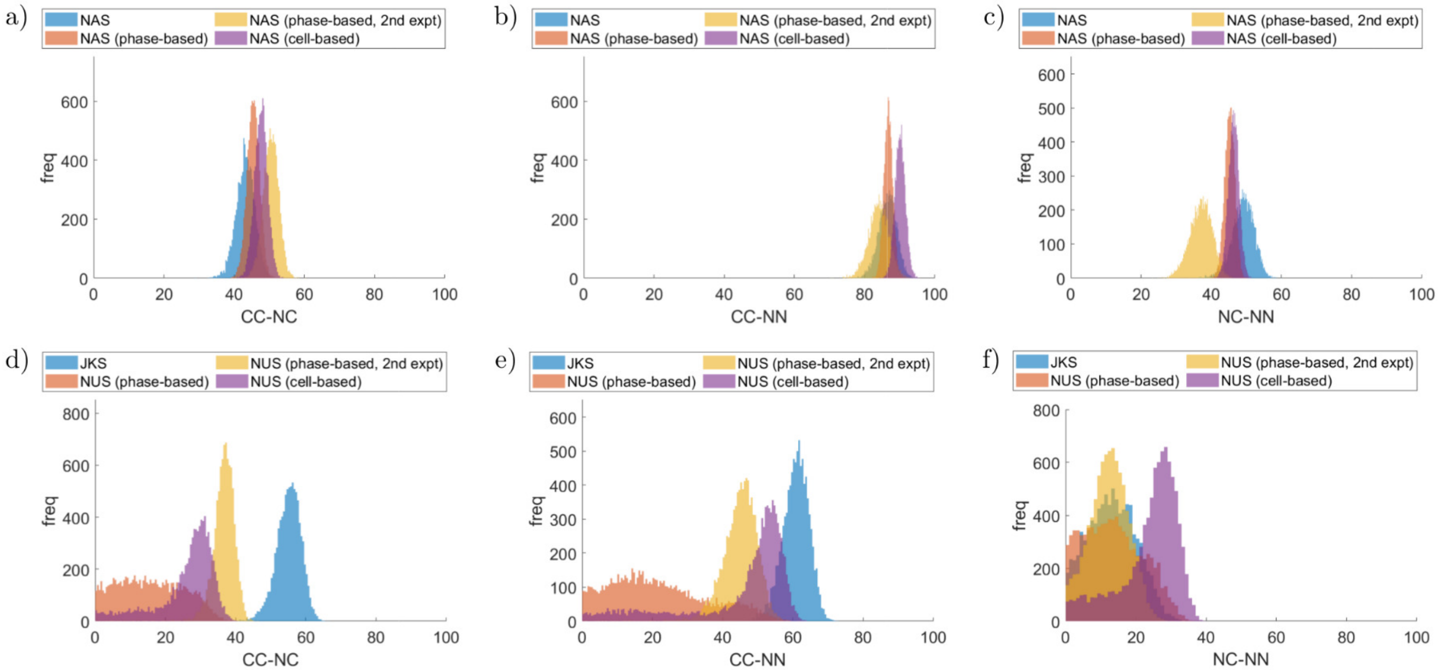

Fig G. Comparison of lengths marginals of jack-knifed (nocodazole), naturally unattached and attached states of CenpC–Ndc80C–Ndc80N triangle. Shown are the length marginals of the CenpC–Ndc80C–Ndc80N triangle in the three conformational states inferred in this study (attached, naturally unattached and jack-knifed), combining results of Examples 3.2, 3.3, 4.1, C.4, and C.5. All lengths are given in nm and have flat priors. See supplement Uninformative priors in S1 Text for the joint priors. Abbreviations are CC for CenpC, NC for Ndc80C and NN for Ndc80N. NAS denotes the (natural) attached state 1 (dominant in metaphase, DMSO), NUS the natural unattached state 2 (dominant in early prometaphase, DMSO) and JKS the jack-knifed state (in nocodazole). The single-state results are shown in blue (Example 3.2, 3.3), red is the two-state phase-based analysis (Example 4.1), yellow the second experiment of the two-state phase-based analysis (Example C.4) and purple the two-state cell-based analysis (Example C.5).

Table C. Inferred conformations of CenpC–Ndc80C–Ndc80N

Example C.1, Attached conformation metaphase. DMSO. $N = 72$, $C = 3$:

(lengths and errors in nm)	CC–NC	CC–NN	NC–NN
triangle, uninformative prior	43.2 ± 2.6	86.5 ± 2.6	49.0 ± 3.2

Example C.2, Jack-knifed conformation metaphase. Nocodazole. $N = 118$, $C = 5$:

(lengths and errors in nm)	CC–NC	CC–NN	NC–NN
triangle, uninformative prior	55.1 ± 3.5	60.9 ± 3.6	13.1 ± 6.7

Example C.3, Prometaphase-metaphase mitotic-phase subsets. DMSO.
$N_{\text{meta}} = 570$, $C_{\text{meta}} = 17$, $N_{\text{late prometa}} = 134$, $C_{\text{late prometa}} = 4$, $N_{\text{early prometa}} = 302$, $C_{\text{early prometa}} = 10$:

	state 1 (attached):			state 2 (natural unattached):					
(lengths and errors in nm)	CC–NC	CC–NN	NC–NN	CC–NC	CC–NN	NC–NN	$p^{(2)}_{\text{meta}}$	$p^{(2)}_{\text{late prometa}}$	$p^{(2)}_{\text{early prometa}}$
triangle, uninformative prior	45.4 ± 1.8	86.7 ± 1.3	45.2 ± 1.4	15.8 ± 9.2	21.9 ± 13.8	13.6 ± 8.2	$(2.25 \pm 2.12)\,\%$	$(12.5 \pm 8.6)\,\%$	$(88.7 \pm 8.2)\,\%$

Example C.4, Prometaphase-metaphase mitotic-phase subsets, second experiment. DMSO.
$N_{\text{meta}} = 152$, $C_{\text{meta}} = 5$, $N_{\text{late prometa}} = 108$, $C_{\text{late prometa}} = 3$, $N_{\text{early prometa}} = 78$, $C_{\text{early prometa}} = 3$:

	state 1 (attached):			state 2 (natural unattached):					
(lengths and errors in nm)	CC–NC	CC–NN	NC–NN	CC–NC	CC–NN	NC–NN	$p^{(2)}_{\text{meta}}$	$p^{(2)}_{\text{late prometa}}$	$p^{(2)}_{\text{early prometa}}$
triangle, uninformative prior	50.5 ± 2.2	84.1 ± 2.9	37.1 ± 3.2	36.5 ± 3.9	44.8 ± 5.6	12.5 ± 5.4	$(3.6 \pm 3.3)\,\%$	$(52.0 \pm 10.1)\,\%$	$(91.9 \pm 6.7)\,\%$

Example C.5, Prometaphase-metaphase cell-based subsets. DMSO.
$N_{\text{meta}} = 570$, $C_{\text{meta}} = 17$, $N_{\text{late prometa}} = 134$, $C_{\text{late prometa}} = 4$, $N_{\text{early prometa}} = 302$, $C_{\text{early prometa}} = 10$:

	state 1 (attached):			state 2 (natural unattached):					
(lengths and errors in nm)	CC–NC	CC–NN	NC–NN	CC–NC	CC–NN	NC–NN	$\tilde{p}^{(2)}_{\text{meta}}$	$\tilde{p}^{(2)}_{\text{late prometa}}$	$\tilde{p}^{(2)}_{\text{early prometa}}$
triangle, uninformative prior	47.6 ± 1.9	90.1 ± 1.5	46.4 ± 1.5	26.0 ± 8.1	45.0 ± 15.1	23.9 ± 8.3	$(17.7 \pm 3.6)\,\%$	$(24.0 \pm 7.4)\,\%$	$(75.0 \pm 5.3)\,\%$

Inference of two states in a mixed state population using experimental data. Inferred posterior means and standard deviations of the triangle lengths of the two states are shown, as well as the state proportions. Abbreviations are CC for CenpC, NC for Ndc80C and NN for Ndc80N. Examples C.1–C.3 are duplicates of Examples 3.2, 3.3 and Example 4.1. Example C.4 shows a second, experimentally equivalent example as Example 4.1. Example C.5 shows the same experimental data to Example 4.1, but split by cell into 31 subsets for the state proportions. Here the $\left\{\tilde{p}^{(\zeta)}\right\}_\zeta$ denote the a-posteriori estimated state proportions, see subsection Cell-based subset analysis inference of mitotic phase variables in S1 Text. The priors for $\left\{\tilde{p}^{(\zeta)}\right\}_\zeta$ are not flat as is the case for the mitotic-phase analysis. Posterior distributions for these lengths and states are compared in Fig G in S1 Text.

could be changed a-posteriori by using an importance weight.

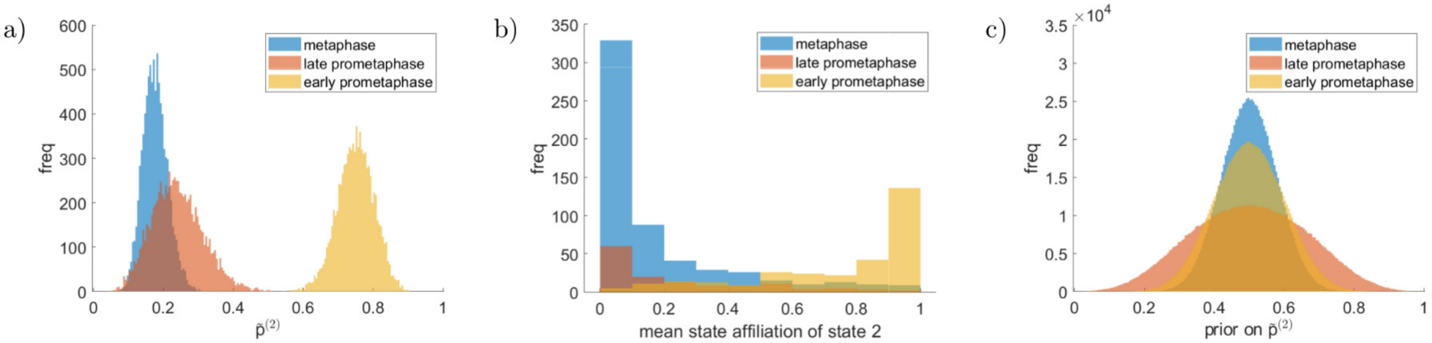

Fig H. A-posteriori estimate of state proportions and affiliations for cell-based experimental prometaphase-metaphase dataset, Example C.5. Panel a) shows the a-posteriori estimated state proportions for each mitotic phase, $\tilde{p}^{(2)}$ (see subsection Cell-based subset analysis inference of mitotic phase variables in S1 Text for definition). The state proportions are pulled away from the boundaries by the prior. Panel b) shows the mean state affiliations pooled within each of the three mitotic phases. The shown mean state affiliations are with respect to the natural unattached state, i.e. kinetochores with a value close to one would be most likely in natural unattached state. Panel c) shows the numerically estimated prior on the a-posteriori estimates of the state proportions $\tilde{p}^{(2)}$ for our cell-numbers and -sizes in each mitotic phase (same for all states). For the cell-based analysis the prior on the state proportions is independent between cells and flat in $[0, 1]$ for each cell. As we have multiple cells in the same mitotic phase, this means that the prior on the a-posteriori estimates of the state proportions $\tilde{p}^{(2)}$ in a mitotic phase are not uniform anymore, but more concentrated around 50%.

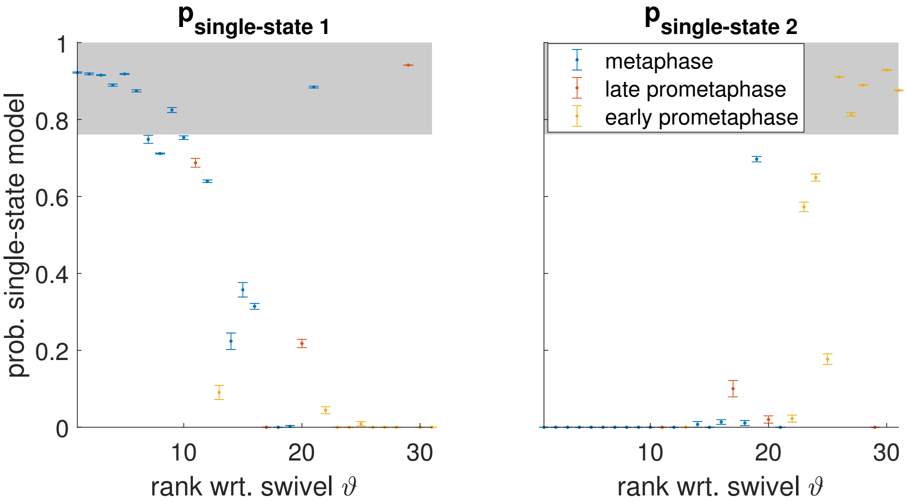

Fig I. Cell-based model comparison of single- vs two-state model for experimental prometaphase-metaphase dataset, Example C.5. The horizontal axis shows the the cells ordered with respect to their mean intrakinetochore swivel ϑ. Mitotic phases are indicated by colour. The vertical axis shows the probability of the single-state model in state 1 and 2, respectively. For a computation of the model comparison see supplementary section Model comparison: Two-state vs single-state model in S1 Text, where we chose $\alpha = 100\%$ for all cells and states here. The grey shading indicates the region, where substantial evidence for the single-state model (i.e. homogeneous cells with all kinetochores in the same state) is found (i.e. Bayes factor ≥ 3.2).

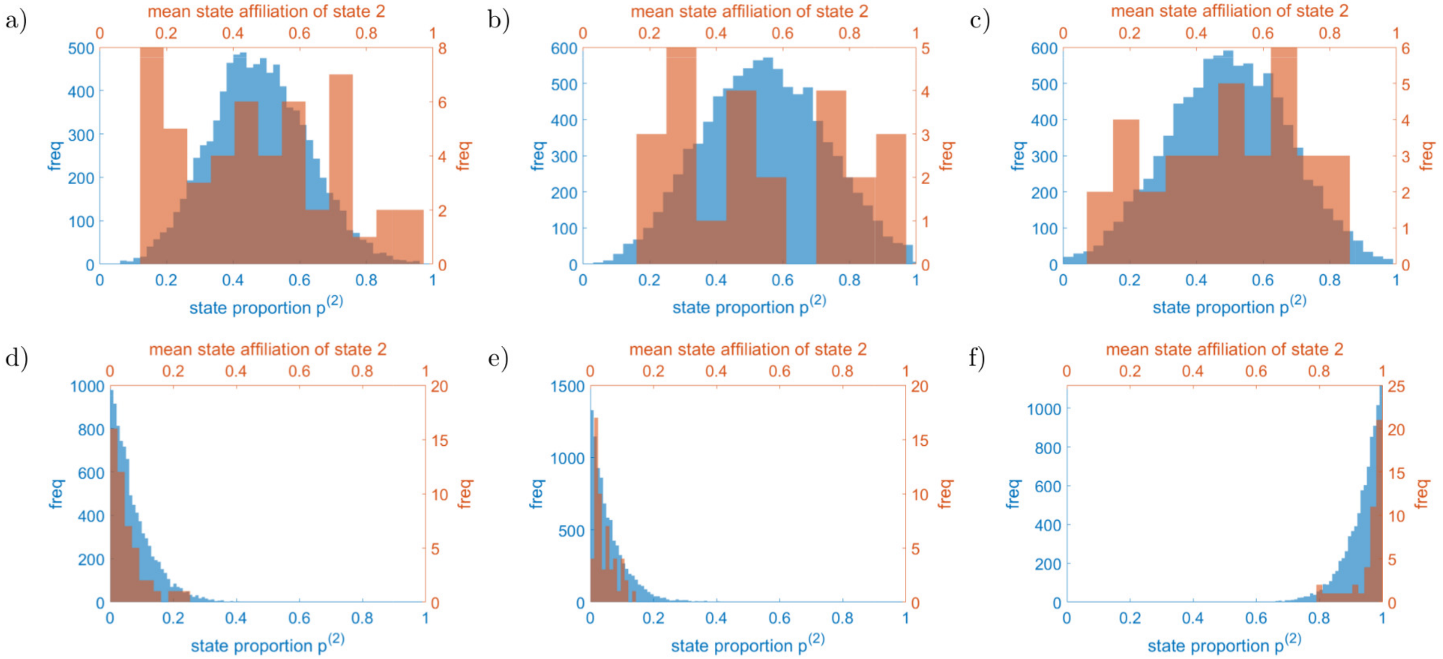

Fig J. State proportions and mean state affiliations for example cells for cell-based experimental prometaphase-metaphase dataset, Example C.5. State proportions of natural unattached state 2 (blue) and mean state affiliations per kinetochore (red) for six cells. Top row shows a cell in metaphase (a), late prometaphase (b) and early prometaphase (c), respectively. Examples of cells in each mitotic phase that have mean state proportions close to 0.5 are shown. In this case ca. 2/3 of the kinetochores of the respective cells are undecided (i.e. mean state affiliation in $[0.24, 076]$; no substantial evidence according to [9]). Bottom row shows again one cell in metaphase (d), late prometaphase (e) and early prometaphase (f), where the examples were chosen to illustrate cells in each mitotic phase that have a mean state proportion closest to 0 (or 1 in early prometaphase). In this case almost no kinetochores are undecided.

Additional supplementary images

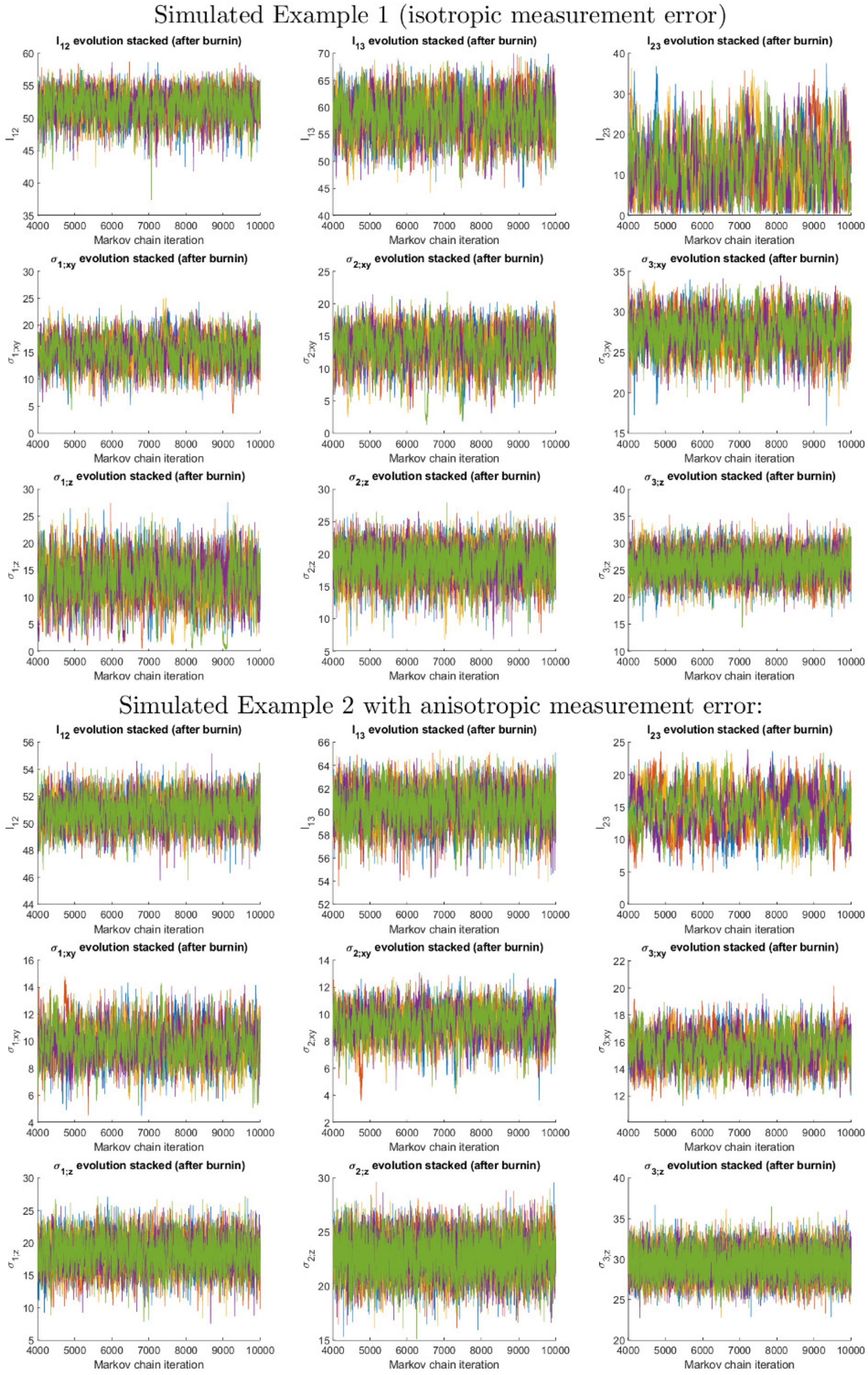

Fig K. Markov chain traces of each of the parameters $l_{ij} = |X_j - X_i|$, $\sigma_{i;d}$ (in nm) for the single-state simulated Examples 1.1, 1.2. Traces are plotted post burnin and sub-sampled to give 10000 samples. Five independent chains are overlain in separate colours.

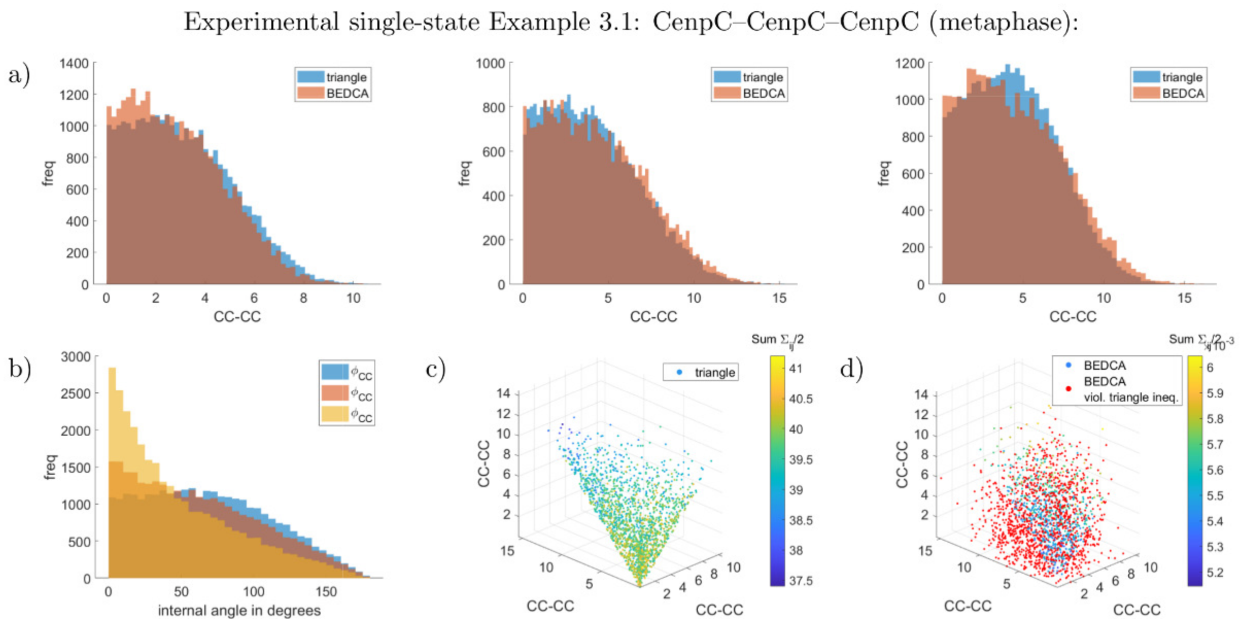

Fig L. Marginal posteriors of the triple labelled CenpC experiment (Example 3.1). Panels are the same as in Fig 3. All lengths are given in nm and have flat priors. See supplement Uninformative priors in S1 Text for the joint priors. Constructing a joint distribution from the three pair-wisely inferred lengths assuming independence yields 62% violations of the triangle inequality (red dots in panel d)).

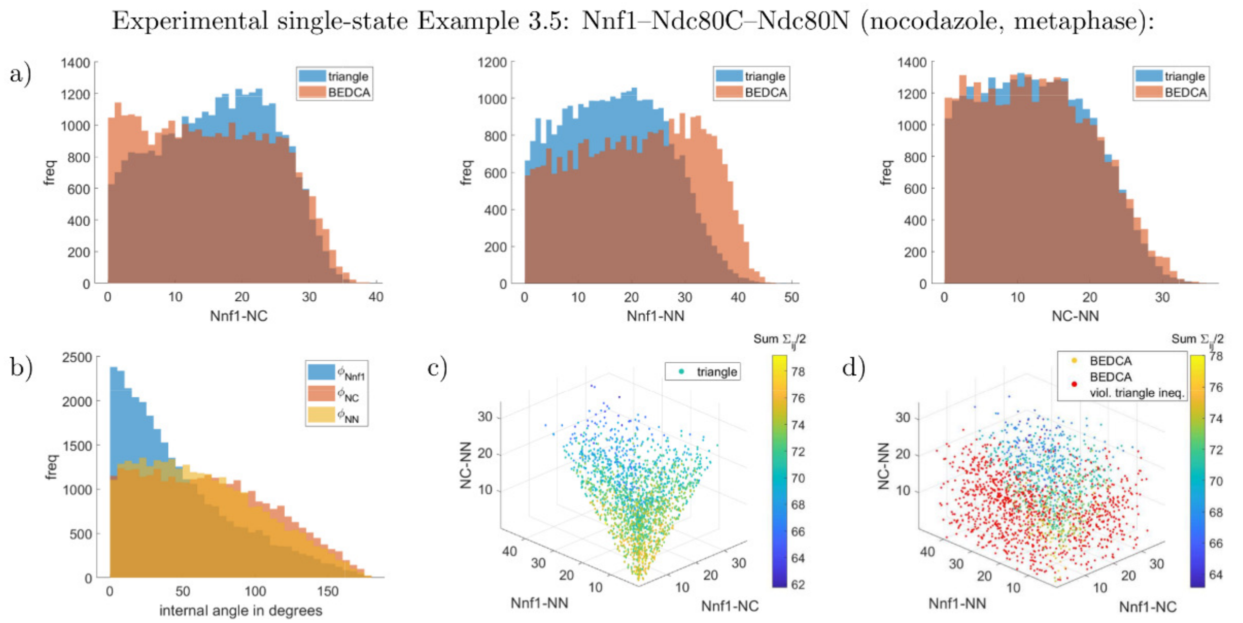

Fig M. Marginal inferred posteriors of the Nnf1–Ndc80C–Ndc80N experiment in nocodazole treatment (Example 3.5). Panels are the same as in Fig 3. All lengths are given in nm and have flat priors. See supplement Uninformative priors in S1 Text for the joint priors. Constructing a joint distribution from the three pair-wisely inferred lengths assuming independence yields 57% violations of the triangle inequality (red dots in panel d)).

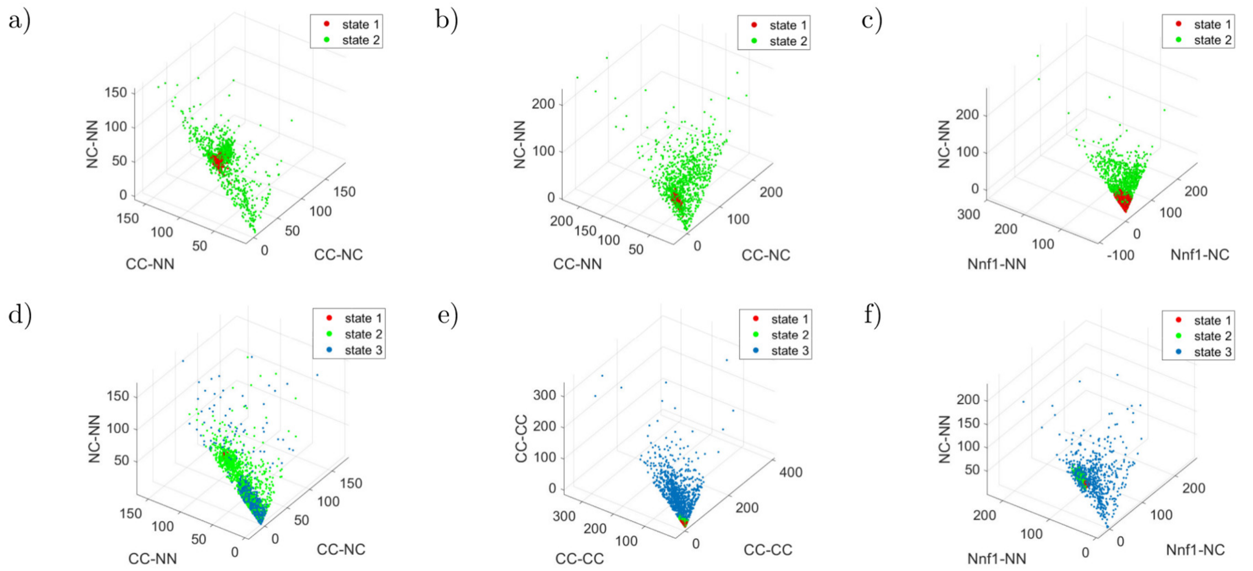

Fig N. Multi-state results of experimental examples. Depicted are the lengths-marginals (in nm) of the multi-state model run on the experimental examples of the main text (subsections Experimental dataset analysis with single-state model algorithm, Experimental dataset analysis with multi-state model algorithm), but containing one more state than the results presented in the main text. Specifically the panels show Example 3.2 (a), Example 3.3 (b), Example 3.5 (c) and Example 4.1 (d), Example 4.2 (e), Example 4.3 (f). State labels are assigned based on prevalence in the datasets, i.e. $p^{(1)} \geq p^{(2)} \geq p^{(3)}$. It can be seen that the additional state is not well-informed and spreads over several hundred nanometres. Note that due to our prior to have at least three measurements in each state, the lengths stay localised to some extent. See supplement Uninformative priors in S1 Text for the joint priors on the lengths.

Additional supplementary tables

Table D. Inferred measurement errors $\sigma_{j;d}$ for single-state simulated Examples 1.1, 1.2 for each fluorophore individually.

(all errors in nm)	$\sigma_{1;xy}$	$\sigma_{1;z}$	$\sigma_{2;xy}$	$\sigma_{2;z}$	$\sigma_{3;xy}$	$\sigma_{3;z}$
Example 1.1 with isotropic measurement error:						
true value	15	15	15	15	25	25
triangle, uninformative prior	15.0 ± 2.7	13.0 ± 4.0	13.2 ± 2.8	18.5 ± 2.5	27.4 ± 2.1	25.9 ± 2.4
Example 1.2 with anisotropic measurement error:						
true value	10	20	10	20	15	30
triangle, uninformative prior	9.8 ± 1.3	18.3 ± 2.4	9.3 ± 1.2	22.7 ± 1.7	15.5 ± 1.1	29.4 ± 1.7

Due to an indistinguishability for the pair-wise methods, these parameters can only be inferred, if at least three fluorophores $J \geq 3$ are used.

Table E. Sizes of experimental datasets.

Single-state experimental examples (see Table 3 for results):				
Example	triple structure	treatment	#kinetochores N	#cells C
3.1	CenpC–CenpC–CenpC		1336	34
3.2	CenpC–Nd80C–Ndc80N	DMSO	72	3
3.3	CenpC–Nd80C–Ndc80N	nocodazole	118	5
3.4	Nnf1–Nd80C–Ndc80N	DMSO	570	18
3.5	Nnf1–Nd80C–Ndc80N	nocodazole	238	8

Two-state experimental examples (see Table 4 for results):							
Example	triple structure	#kinetochores N, metaphase	#cells C, metaphase	#kinetochores N, late prometaphase	#cells C, late prometaphase	#kinetochores N, early prometaphase	#cells C, early prometaphase
4.1	CenpC-Ndc80C-Ndc80N	570	17	134	4	302	10
4.2	CenpC-CenpC-CenpC	1336	34				
4.3	Nnf1-Ndc80C-Ndc80N	570	18				

The number of kinetochores and number of cells are given after image processing and quality control.

Table F. Inferred measurement errors $\sigma_{j;d}$ for single-state experimental Examples 3.1–3.5 for each fluorophore individually.

(all errors in nm)	$\sigma_{\mathrm{CC};xy}$	$\sigma_{\mathrm{CC};z}$	$\sigma_{\mathrm{Nnf1};xy}$	$\sigma_{\mathrm{Nnf1};z}$	$\sigma_{\mathrm{NC};xy}$	$\sigma_{\mathrm{NC};z}$	$\sigma_{\mathrm{NN};xy}$	$\sigma_{\mathrm{NN};z}$
DMSO-treated experimental single-state examples:								
Example 3.1, A488, triangle, uninf. prior	13.1 ± 0.3	25.4 ± 0.9	/	/	/	/	/	/
Example 3.1, A568, triangle, uninf. prior	9.0 ± 0.4	20.3 ± 1.1	/	/	/	/	/	/
Example 3.1, A647, triangle, uninf. prior	16.1 ± 0.4	40.4 ± 1.0	/	/	/	/	/	/
Example 3.2, triangle, uninf. prior	9.4 ± 3.1	16.5 ± 7.7	/	/	12.1 ± 1.8	33.6 ± 4.1	13.0 ± 2.7	28.2 ± 6.2
Example 3.4, triangle, uninf. prior	/	/	10.6 ± 0.8	25.1 ± 2.0	13.1 ± 0.6	30.7 ± 1.5	9.5 ± 0.9	27.6 ± 2.0
Nocodazole-treated experimental single-state examples:								
Example 3.3, triangle, uninf. prior	13.8 ± 3.3	16.3 ± 7.3	/	/	18.2 ± 2.2	35.5 ± 3.9	18.4 ± 2.6	39.1 ± 4.0
Example 3.5, triangle, uninf. prior	/	/	20.0 ± 2.7	32.7 ± 2.7	15.0 ± 2.5	26.6 ± 2.7	23.6 ± 1.8	31.5 ± 2.6

Here we abbreviate CC for CenpC, NC for Ndc80C and NN for Ndc80N. The same fluorophores were used across all examples to mark the various structures of the kinetochore (apart from the triple-CenpC, Example 3.1, where A568 is the secondary antibody of the CenpC in the other examples). The table shows consistency of the inference results of the same fluorophore between different examples. Here, the fluorophore for CenpC exhibits the smallest measurement error. Slight differences can be seen for the same fluorophores between DMSO- and nocodazole-treated cells (Nnf1, Ndc80N). Due to an indistinguishability for the pair-wise methods, these parameters can only be inferred, if at least three fluorophores $J \geq 3$ are used.

Table G. Inferred measurement errors $\sigma_{j;d}$ for two-state experimental Examples 4.1–4.3 for each fluorophore individually.

Example 4.1, CenpC–Ndc80C–Ndc80N (DMSO, prometaphase-metaphase):

	state 1:					
(all errors in nm)	$\sigma^{(1)}_{\mathrm{CC};xy}$	$\sigma^{(1)}_{\mathrm{CC};z}$	$\sigma^{(1)}_{\mathrm{NC};xy}$	$\sigma^{(1)}_{\mathrm{NC};z}$	$\sigma^{(1)}_{\mathrm{NN};xy}$	$\sigma^{(1)}_{\mathrm{NN};z}$
triangle, uninf. prior	15.5 ± 1.1	33.0 ± 2.3	17.1 ± 0.9	36.8 ± 1.7	12.6 ± 1.3	29.2 ± 2.3
	state 2:					
(all errors in nm)	$\sigma^{(2)}_{\mathrm{CC};xy}$	$\sigma^{(2)}_{\mathrm{CC};z}$	$\sigma^{(2)}_{\mathrm{NC};xy}$	$\sigma^{(2)}_{\mathrm{NC};z}$	$\sigma^{(2)}_{\mathrm{NN};xy}$	$\sigma^{(2)}_{\mathrm{NN};z}$
triangle, uninf. prior	25.6 ± 3.5	47.2 ± 3.7	7.4 ± 4.2	42.3 ± 3.3	27.0 ± 3.0	33.6 ± 4.7

Example 4.2, CenpC–CenpC–CenpC (metaphase):

	state 1:					
(all errors in nm)	$\sigma^{(1)}_{\mathrm{CC};xy}$	$\sigma^{(1)}_{\mathrm{CC};z}$	$\sigma^{(1)}_{\mathrm{CC};xy}$	$\sigma^{(1)}_{\mathrm{CC};z}$	$\sigma^{(1)}_{\mathrm{CC};xy}$	$\sigma^{(1)}_{\mathrm{CC};z}$
triangle, uninf. prior	10.3 ± 0.9	19.2 ± 1.7	8.9 ± 0.7	19.7 ± 1.3	14.5 ± 0.7	38.1 ± 1.2
	state 2:					
(all errors in nm)	$\sigma^{(2)}_{\mathrm{CC};xy}$	$\sigma^{(2)}_{\mathrm{CC};z}$	$\sigma^{(2)}_{\mathrm{CC};xy}$	$\sigma^{(2)}_{\mathrm{CC};z}$	$\sigma^{(2)}_{\mathrm{CC};xy}$	$\sigma^{(2)}_{\mathrm{CC};z}$
triangle, uninf. prior	22.3 ± 2.0	46.7 ± 5.0	7.9 ± 3.1	21.4 ± 7.3	22.0 ± 1.9	51.5 ± 4.6

Example 4.3, Nnf1–Ndc80C–Ndc80N (DMSO, metaphase):

	state 1:					
(all errors in nm)	$\sigma^{(1)}_{\mathrm{Nnf1};xy}$	$\sigma^{(1)}_{\mathrm{Nnf1};z}$	$\sigma^{(1)}_{\mathrm{NC};xy}$	$\sigma^{(1)}_{\mathrm{NC};z}$	$\sigma^{(1)}_{\mathrm{NN};xy}$	$\sigma^{(1)}_{\mathrm{NN};z}$
triangle, uninf. prior	7.3 ± 1.4	21.8 ± 3.0	10.8 ± 1.0	29.1 ± 2.3	7.5 ± 1.3	21.9 ± 3.5
	state 2:					
(all errors in nm)	$\sigma^{(2)}_{\mathrm{Nnf1};xy}$	$\sigma^{(2)}_{\mathrm{Nnf1};z}$	$\sigma^{(2)}_{\mathrm{NC};xy}$	$\sigma^{(2)}_{\mathrm{NC};z}$	$\sigma^{(2)}_{\mathrm{NN};xy}$	$\sigma^{(2)}_{\mathrm{NN};z}$
triangle, uninf. prior	11.5 ± 4.9	33.8 ± 8.4	19.5 ± 2.6	35.7 ± 7.1	12.5 ± 4.4	42.0 ± 7.9

Inference of two states in a mixed state population using experimental data. Inferred posterior means and standard deviations of the measurement errors of the two states are shown. Abbreviations are CC for CenpC, NC for Ndc80C and NN for Ndc80N. For Example 4.1 we do not observe a significant difference of the measurement errors between the two states, while for Examples 4.2, 4.3 the two states have significantly different errors, with the minority state 2 typically (in all significant cases) exhibiting a higher measurement error. A comparison between the examples is not clear, because it is not clear how the different states relate to each other.

Table H. Mitotic-phase versus tension parameters for two-state experimental Example 4.1.

Statistic (averages per cell)	metaphase	late prometaphase	early prometaphase
metaphase plate width† (in nm)	984 ± 36	1054 ± 79	/
sister axis angle to metaphase plate† (in deg)	30.6 ± 4.9	57.7 ± 2.2	/
swivel ϑ (in deg)	49.0 ± 2.4	65.6 ± 5.3	81.7 ± 1.3
sister-sister (K-K) distance l_{ss} (in nm)	1322 ± 27	1297 ± 37	1162 ± 59

† The metaphase plate width is defined as the square root of the smallest eigenvalue of the covariance of the CenpC locations. The corresponding eigenvector is used as the normal of the metaphase plate.

Table I. Inferred conformations of CenpC–Ndc80C–Ndc80N in prometaphase-metaphase, single-state results compared with two-state

Example I.1, Attached conformation metaphase. DMSO. $N = 72$, $C = 3$:

(lengths and errors in nm)	CenpC–Ndc80C	CenpC–Ndc80N	Ndc80C–Ndc80N
BEDCA, [4]	45.7 ± 3.2	86.7 ± 2.5	54.2 ± 2.9
triangle, uninformative prior	43.2 ± 2.6	86.5 ± 2.6	49.0 ± 3.2

Example I.2, Jack-knifed conformation metaphase. Nocodazole. $N = 118$, $C = 5$:

(lengths and errors in nm)	CenpC–Ndc80C	CenpC–Ndc80N	Ndc80C–Ndc80N
BEDCA, [4]	53.1 ± 8.1	62.1 ± 3.9	11.6 ± 7.2
triangle, uninformative prior	55.1 ± 3.5	60.9 ± 3.6	13.1 ± 6.7

Example I.3, Prometaphase-metaphase mitotic-phase subsets. DMSO.
$N_{\text{meta}} = 570$, $C_{\text{meta}} = 17$, $N_{\text{late prometa}} = 134$, $C_{\text{late prometa}} = 4$, $N_{\text{early prometa}} = 302$, $C_{\text{early prometa}} = 10$:

(lengths and errors in nm)	CenpC–Ndc80C	CenpC–Ndc80N	Ndc80C–Ndc80N
triangle, uninf.prior, attached state	45.4 ± 1.8	86.7 ± 1.3	45.2 ± 1.4
triangle, uninf.prior, natural unattached state	15.8 ± 9.2	21.9 ± 13.8	13.6 ± 8.2

Example I.4, Metaphase. DMSO. $N = 570$, $C = 17$:

(lengths and errors in nm)	CenpC–Ndc80C	CenpC–Ndc80N	Ndc80C–Ndc80N
BEDCA, [4]	48.1 ± 2.1	86.5 ± 1.2	45.6 ± 2.2
triangle, uninformative prior	44.7 ± 1.9	86.4 ± 1.2	45.1 ± 1.5

Example I.5, Late prometaphase. DMSO. $N = 134$, $C = 4$:

(lengths and errors in nm)	CenpC–Ndc80C	CenpC–Ndc80N	Ndc80C–Ndc80N
BEDCA, [4]	33.5 ± 12.4	79.6 ± 3.2	29.1 ± 13.0
triangle, uninformative prior	42.3 ± 4.1	80.0 ± 3.3	43.0 ± 3.9

Example I.6, Early prometaphase. DMSO. $N = 302$, $C = 10$:

(lengths and errors in nm)	CenpC–Ndc80C	CenpC–Ndc80N	Ndc80C–Ndc80N
BEDCA, [4]	14.6 ± 8.6	40.2 ± 13.9	12.5 ± 7.7
triangle, uninformative prior	15.5 ± 9.2	22.5 ± 13.4	13.5 ± 8.5

Examples I.1–I.3 are duplicates of Examples 3.2, 3.3 and Example 4.1 (omitting state proportions), Examples I.4–I.6 are single-state results on subsets of the prometaphase-metaphase dataset used for Example 4.1, identified by the mitotic phase of the cells. Means and standard deviations of the inferred length posteriors are given, comparing the single-state triangle inference presented in this paper with the single-state pair-wise method of [4].

Table J. Inferred conformations of CenpC–Ndc80C–Ndc80N in prometaphase-metaphase, two-state pair-wise results compared to two-state triangle

Example J.1, Prometaphase-metaphase mitotic-phase subsets. DMSO.
$N_{\text{meta}} = 570$, $C_{\text{meta}} = 17$, $N_{\text{late prometa}} = 134$, $C_{\text{late prometa}} = 4$, $N_{\text{early prometa}} = 302$, $C_{\text{early prometa}} = 10$:

	state 1 (attached):			state 2 (natural unattached):					
(lengths and errors in nm)	CC–NC	CC–NN	NC–NN	CC–NC	CC–NN	NC–NN	$p^{(2)}_{\text{meta}}$	$p^{(2)}_{\text{late prometa}}$	$p^{(2)}_{\text{early prometa}}$
triangle, uninformative prior	45.4 ± 1.8	86.7 ± 1.3	45.2 ± 1.4	15.8 ± 9.2	21.9 ± 13.8	13.6 ± 8.2	$(2.25 \pm 2.12)\,\%$	$(12.5 \pm 8.6)\,\%$	$(88.7 \pm 8.2)\,\%$

Example J.2, Prometaphase-metaphase mitotic-phase subsets. DMSO.
$N_{\text{meta}} = 570$, $C_{\text{meta}} = 17$, $N_{\text{late prometa}} = 134$, $C_{\text{late prometa}} = 4$, $N_{\text{early prometa}} = 302$, $C_{\text{early prometa}} = 10$:

	state 1:			state 2:					
(lengths and errors in nm)	CC–NC	CC–NN	NC–NN	CC–NC	CC–NN	NC–NN	$p^{(2)}_{\text{meta}}$	$p^{(2)}_{\text{late prometa}}$	$p^{(2)}_{\text{early prometa}}$
length, uninformative prior	49.3 ± 2.5			16.4 ± 10.0			$(8.31 \pm 7.00)\,\%$	$(71.8 \pm 16.7)\,\%$	$(89.2 \pm 9.1)\,\%$
length, uninformative prior		86.7 ± 1.2			38.5 ± 14.3		$(1.57 \pm 1.47)\,\%$	$(11.5 \pm 8.6)\,\%$	$(86.7 \pm 9.9)\,\%$
length, uninformative prior			49.1 ± 3.7			13.4 ± 9.3	$(27.5 \pm 18.2)\,\%$	$(50.0 \pm 24.1)\,\%$	$(85.8 \pm 19.0)\,\%$

Model comparison of single-state vs two-state model for Example J.2.

	CenpC–Ndc80C	CenpC–Ndc80N	Ndc80C–Ndc80N
$p_{\text{two-states;meta}}$	$(36.6 \pm 2.1)\,\%$	$(7.95 \pm 0.30)\,\%$	$(36.7 \pm 2.0)\,\%$
$p_{\text{two-states;late prometa}}$	$(46.0 \pm 4.8)\,\%$	$(18.1 \pm 1.7)\,\%$	$(60.8 \pm 2.9)\,\%$
$p_{\text{two-states;early prometa}}$	$(12.8 \pm 1.0)\,\%$	$(18.7 \pm 2.6)\,\%$	$(6.91 \pm 0.26)\,\%$

Example J.1 is a duplicate of Example 4.1, while each row of Example C.2 is based on the same dataset with mitotic-phase subsets as Example 4.1 using two-state pair-wise correction. Inferred posterior means and standard deviations of the triangle lengths of the two states are shown, as well as the state proportions. Abbreviations are CC for CenpC, NC for Ndc80C and NN for Ndc80N. Like for the triangle results on the same dataset, a model comparison (see supplemental section) gives no substantial evidence for two states in any mitotic phase – see bottom table. Constructing a joint distribution from the three pair-wisely inferred lengths assuming independence yields 1% and 78% violations of the triangle inequality for state 1 and state 2, respectively.

Table K. Correlations per kinetochore of mean state affiliation and tension parameters for two-state experimental examples.

	all mitotic phases	metaphase	late prometaphase	early prometaphase
**Example 4.1, CenpC–Ndc80C–Ndc80N (DMSO, prometaphase-metaphase)**				
swivel ϑ	$+0.488$ ($p = 2.8 \times 10^{-61}$)	$+0.278$ ($p = 1.4 \times 10^{-11}$)	$+0.109$ ($p = 2.1 \times 10^{-1}$)	$+0.375$ ($p = 1.7 \times 10^{-11}$)
sister-sister distance l_{ss}	-0.245 ($p = 3.6 \times 10^{-15}$)	-0.023 ($p = 5.9 \times 10^{-1}$)	$+0.046$ ($p = 6.0 \times 10^{-1}$)	-0.190 ($p = 9.1 \times 10^{-4}$)
**Example C.4, CenpC–Ndc80C–Ndc80N (DMSO, prometaphase-metaphase), second experiment**				
swivel ϑ	$+0.300$ ($p = 1.9 \times 10^{-8}$)	$+0.276$ ($p = 5.9 \times 10^{-4}$)	$+0.402$ ($p = 1.3 \times 10^{-8}$)	-0.185 ($p = 1.1 \times 10^{-1}$)
sister-sister distance l_{ss}	-0.319 ($p = 2.0 \times 10^{-9}$)	$+0.145$ ($p = 8.6 \times 10^{-1}$)	-0.544 ($p = 1.0 \times 10^{-15}$)	$+0.087$ ($p = 4.5 \times 10^{-1}$)
**Example C.5, CenpC–Ndc80C–Ndc80N (DMSO, prometaphase-metaphase), cell-based analysis**				
swivel ϑ	$+0.403$ ($p = 1.8 \times 10^{-40}$)	$+0.318$ ($p = 6.7 \times 10^{-15}$)	-0.099 ($p = 2.5 \times 10^{-1}$)	$+0.327$ ($p = 5.9 \times 10^{-9}$)
sister-sister distance l_{ss}	-0.234 ($p = 5.3 \times 10^{-14}$)	$+0.036$ ($p = 3.9 \times 10^{-1}$)	-0.591 ($p = 5.0 \times 10^{-1}$)	-0.230 ($p = 5.3 \times 10^{-5}$)

Provided there is enough data for a significant statement (i.e. $p \leq 5 \times 10^{-2}$), there is a positive correlation between the mean state affiliation $\bar{\zeta}^n$ of a kinetochore and its mean swivel ϑ and a negative correlation between its mean state affiliation and its mean sister-sister distance l_{ss}. This correlation is also present in the cell-based subset analysis when information on the mitotic phase is not provided to the multi-state inference algorithm. This indicates that the attached state conformation is associated with a more stretched mechanical condition.

Table L. Cell-based model preference, subdivided by mitotic phase: two-state experimental Example C.5.

Statistic (averages per cell)	metaphase	late prometaphase	early prometaphase
#cells with subst. evidence for some single-state model	7	1	4
#undecided cells between models	7	1	3
#cells with subst. evidence for two-state model	3	2	3

The two-state and one-state (either state) models are a-priori equiprobable. Substantial evidence was acknowledged, if the estimated probability of the respective model was significantly (in terms of sampling error) above the threshold of 76%. See supplementary subsection Model comparison: Two-state vs single-state model in S1 Text for details on the model comparison.

References

1. Sherlock C, Roberts G. Optimal scaling of the random walk Metropolis on elliptically symmetric unimodal targets. Bernoulli, 2009; 15(3):774–798.

2. Gelman A, Carlin JB, Stern HS, Rubin DB. Bayesian Data Analysis. Chapman&Hall, 2nd edition, 2004.

3. Churchman LS, Flyvbjerg H, Spudich JA. A Non-Gaussian Distribution Quantifies Distances Measured with Fluorescence Localization Techniques. Biophysical Journal, 2006; 90:668–671.

4. Germanova TE, Roscioli E, Harrison JU, McAinsh AD, Burroughs NJ. Subcellular Euclidean distance measurements with multicolor fluorescence localization imaging in cultured cells. STAR Protocols, 2021; 2(4):100774

5. Roscioli E, Germanova TE, Smith CA, Embacher PA, Erent M, Burroughs NJ, et al. Ensemble-level organization of human kinetochores and evidence for distinct tension and attachment sensors. Cell Reports, 2020; 31(4).

6. Niekamp S, Sung J, Huynh W, Bhabha G, Vale RD, Stuurman N. Nanometer-accuracy distance measurements between fluorophores at the single-molecule level. PNAS, 2019; 116(10):4275–4284.

7. Churchman LS, Ökten Z, Rock RS, Dawson JF, Spudich JA. Single molecule high-resolution colocalization of Cy3 and Cy5 attached to macromolecules measures intramolecular distances through time. PNAS, 2005; 102(5):1419–1423.

8. Brémaud P. Probability Theory and Stochastic Processes. Springer Nature Switzerland, Universitext, 2020.

9. Kass RE, Raftery AE. Bayes Factors. Journal of the American Statistical Association, 1995; 90(430):773–795.
